# Structural (XRD) Characterization and an Analysis of H-Bonding Motifs in Some Tetrahydroxidohexaoxidopentaborate(1-) Salts of *N*-Substituted Guanidinium Cations †

**DOI:** 10.3390/molecules28073273

**Published:** 2023-04-06

**Authors:** Michael A. Beckett, Simon J. Coles, Peter N. Horton, Thomas A. Rixon

**Affiliations:** 1 School of Natural Sciences, Bangor University, Bangor, Gwynedd LL57 2UW, UK; 2 Chemistry Department, University of Southampton, Southampton SO17 1BJ, UK

**Keywords:** borate, guanidinium salts, H-bonding, oxidoborate, pentaborate(1-), R_2_^2^(8) motifs, tetrahydroxidohexaoxidopentaborate(1-) salts, XRD

## Abstract

The synthesis and characterization of six new substituted guanidium tetrahydroxidohexaoxidopentaborate(1-) salts are reported: [C(NH_2_)_2_(NHMe)][B_5_O_6_(OH)_4_]·H_2_O (**1**), [C(NH_2_)_2_(NH{NH_2_})][B_5_O_6_(OH)_4_] (**2**), [C(NH_2_)_2_(NMe_2_)][B_5_O_6_(OH)_4_] (**3**), [C(NH_2_)(NMe_2_)_2_][B_5_O_6_(OH)_4_] (**4**), [C(NHMe)(NMe_2_)_2_][B_5_O_6_(OH)_4_]·B(OH)_3_ (**5**), and [TBDH][B_5_O_6_(OH)_4_] (**6**) (TBD = 1,5,7-triazabicyclo [4.4.0]dec-5-ene). Compounds **1**–**6** were prepared as crystalline salts from basic aqueous solution via self-assembly processes from B(OH)_3_ and the appropriate substituted cation. Compounds **1**–**6** were characterized by spectroscopic (NMR and IR) and by single-crystal XRD studies. A thermal (TGA) analysis on compounds **1**–**3** and **6** demonstrated that they thermally decomposed via a multistage process to B_2_O_3_ at >650 °C. The low temperature stage (<250 °C) was endothermic and corresponded to a loss of H_2_O. Reactant stoichiometry, solid-state packing, and H-bonding interactions are all important in assembling these structures. An analysis of H-bonding motifs in known unsubstituted guanidinium salts [C(NH_2_)_3_]_2_[B_4_O_5_(OH)_4_]·2H_2_O, [C(NH_2_)_3_][B_5_O_6_(OH)_4_]·H_2_O, and [C(NH_2_)_3_]_3_[B_9_O_12_(OH)_6_] and in compounds **1**–**6** revealed that two important H-bonding R_2_^2^(8) motifs competed to stabilize the observed structures. The guanidinium cation formed charge-assisted pincer cation–anion H-bonded rings as a major motif in [C(NH_2_)_3_]_2_[B_4_O_5_(OH)_4_]·2H_2_O and [C(NH_2_)_3_]_3_[B_9_O_12_(OH)_6_], whereas the anion–anion ring motif was dominant in [C(NH_2_)_3_][B_5_O_6_(OH)_4_]·H_2_O and in compounds **1**–**6**. This behaviour was consistent with the stoichiometry of the salt and packing effects also strongly influencing their solid-state structures.

## 1. Introduction

Poly(hydroxidooxidoborate) compounds are well-known and are represented by many naturally occurring minerals [1,2,3,4,5,6] and by synthetic analogues [7,8,9,10,11]. These compounds are structurally diverse and are comprised of hydroxidooxidoborate anions paired with various cationic units. Structural features include simple neutral transition-metal complexes [11], insular salts (main group cations, transition-metal complexes and organic cations) [8,9,10,11], and many more highly condensed species comprised of 2-D chains or 3-D networks [1,2,3,4,5,6,7]. Some of these compounds, e.g., Na_2_B_4_O_5_(OH)_4_·8H_2_O (borax) and the zinc borate ZnB_3_O_4_(OH)_3_, are of great importance for many industrial applications [12,13,14]. Other compounds, e.g., β-BaB_2_O_4_ (BBO) have more specialized small-scale applications [4] and the physicochemical properties of others are currently being actively investigated for new potential applications [10,11].

The synthesis of new polyborate [15] salts is easily achieved through simple aqueous synthetic procedures or through solvothermal/hydrothermal methods [10,11]. The nitrogen-rich base guanidine, (H_2_N)_2_C = NH, is a strong base (p*K*_aH_ of its conjugate acid = ca. 13.6 [16]) and organic bases of this strength readily form, when protonated, polyborate salts. Usually, solid-state interactions in polyborate chemistry favour specific cation–anion combinations [10] and therefore it is surprising to find that *three* guanidinium polyborate salts of differing stoichiometries have been structurally characterized by single-crystal X-ray diffraction studies: [C(NH_2_)_3_]_2_[B_4_O_5_(OH)_4_]·2H_2_O [17], [C(NH_2_)_3_][B_5_O_6_(OH)_4_]·H_2_O [18], and [C(NH_2_)_3_]_3_[B_9_O_12_(OH)_6_] [8,19]. The tetraborate(2-) salt, [C(NH_2_)_3_]_2_[B_4_O_5_(OH)_4_]·2H_2_O, was first reported in 1921 [20] and crystallographically characterized in 1985 [17]. Likewise, pentaborate(1-) salts of various degrees of hydration, [C(NH_2_)_3_][B_5_O_6_(OH)_4_]·xH_2_O have been described in early literature [21] but [C(NH_2_)_3_][B_5_O_6_(OH)_4_]·H_2_O was crystallographically characterized in 2020 together with its spectral, optical, thermal, and third-order NLO properties [18]. The large-scale high temperature synthesis of a salt containing the structurally rare nonaborate(3-) anion, [C(NH_2_)_3_]_3_[B_9_O_12_(OH)_6_], was reported in 2000 [19]. [C(NH_2_)_3_]_3_[B_9_O_12_(OH)_6_] has been used as a precursor to BN powders [22].

**Figure 1:**
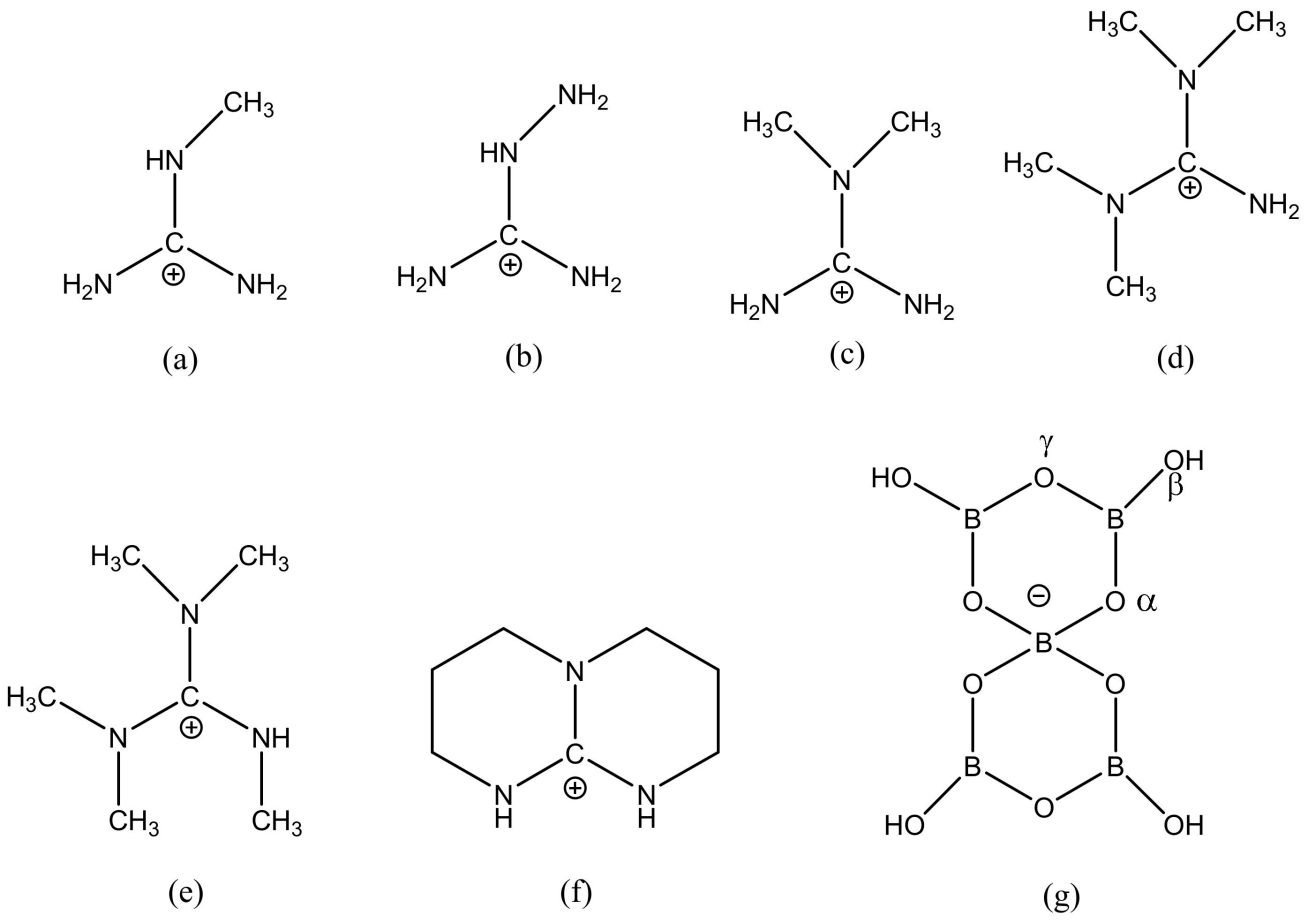
Schematic drawings of the cations and anions found in compounds **1**–**6**: (**a**) *N*-methyl guanidinium(+1) in **1**; (**b**) *N*-aminoguanidinium(+1) in **2**; (**c**) *N*,*N*-dimethylguanidinium(+1) in **3**; (**d**) *N*,*N*,*N*′,*N*′-tetramethylguanidinium(1+) in **4**; (**e**) *N*,*N*,*N*′,*N*′,*N*″-pentamethylguanidinium(1+) in **5**; (**f**) TBDH (TBD = 1,5,7-triazabicyclo[4.4.0]dec-5-ene) in **6**; (**g**) tetrahydroxidohexaoxidopentaborate(1-) found in compounds **1**–**6** with H-bond acceptor sites labelled as α, β, or γ.

The guanidinium cation is therefore particularly well-suited in stabilizing polyborate anions in the solid state, whereas other nonmetal cations are in general less adaptable. The small size, high symmetry (*D*_3*h*_), and high H-bond donor capacity may be contributing factors for this [23,24]. In this manuscript, we investigated the synthesis of new polyborate salts by crystallization from aqueous solutions containing B(OH)_3_ and the substituted guanidinium cations. Six new tetrahydroxidohexaoxidopentaborate(1-) salts were obtained (see Figure 1 for schematic structures) and herein we report their synthesis and their solid-state structures as determined by single-crystal XRD studies. Their solid-state H-bond interactions were analysed together with those found in the polyborate salts of the unsubstituted guanidinium cations as a potential means of accounting for this behaviour [25,26].

## 2. Results and Discussion

### 2.1. Synthesis

The three structurally characterized guanidinium polyborate salts [C(NH_2_)_3_]_2_[B_4_O_5_(OH)_4_]·2H_2_O [17], [C(NH_2_)_3_][B_5_O_6_(OH)_4_]·H_2_O [18], and [C(NH_2_)_3_]_3_[B_9_O_12_(OH)_6_] [19] were all prepared by crystallization from aqueous solutions containing [C(NH_2_)_3_]_2_CO_3_ and B(OH)_3_ at various temperatures and ratios [8]. Two alternative syntheses of [C(NH_2_)_3_]_3_[B_9_O_12_(OH)_6_] have been reported using either (i) [C(NH_2_)_3_]Cl, Na_2_B_4_O_7_·5H_2_O and B(OH)_3_ or (ii) [C(NH_2_)_3_]_2_[B_4_O_5_(OH)_4_]·2H_2_O and B(OH)_3_ in appropriate ratios [19]. It is well-known that B(OH)_3_, and other borate salts, exists in alkaline aqueous solution as equilibrium mixtures of numerous polyborate anions [27,28]. The guanidinium cation that is present templates the crystallization of specific products under the reaction conditions in what can be described as self-assembly processes [29,30].

**Scheme 1:**
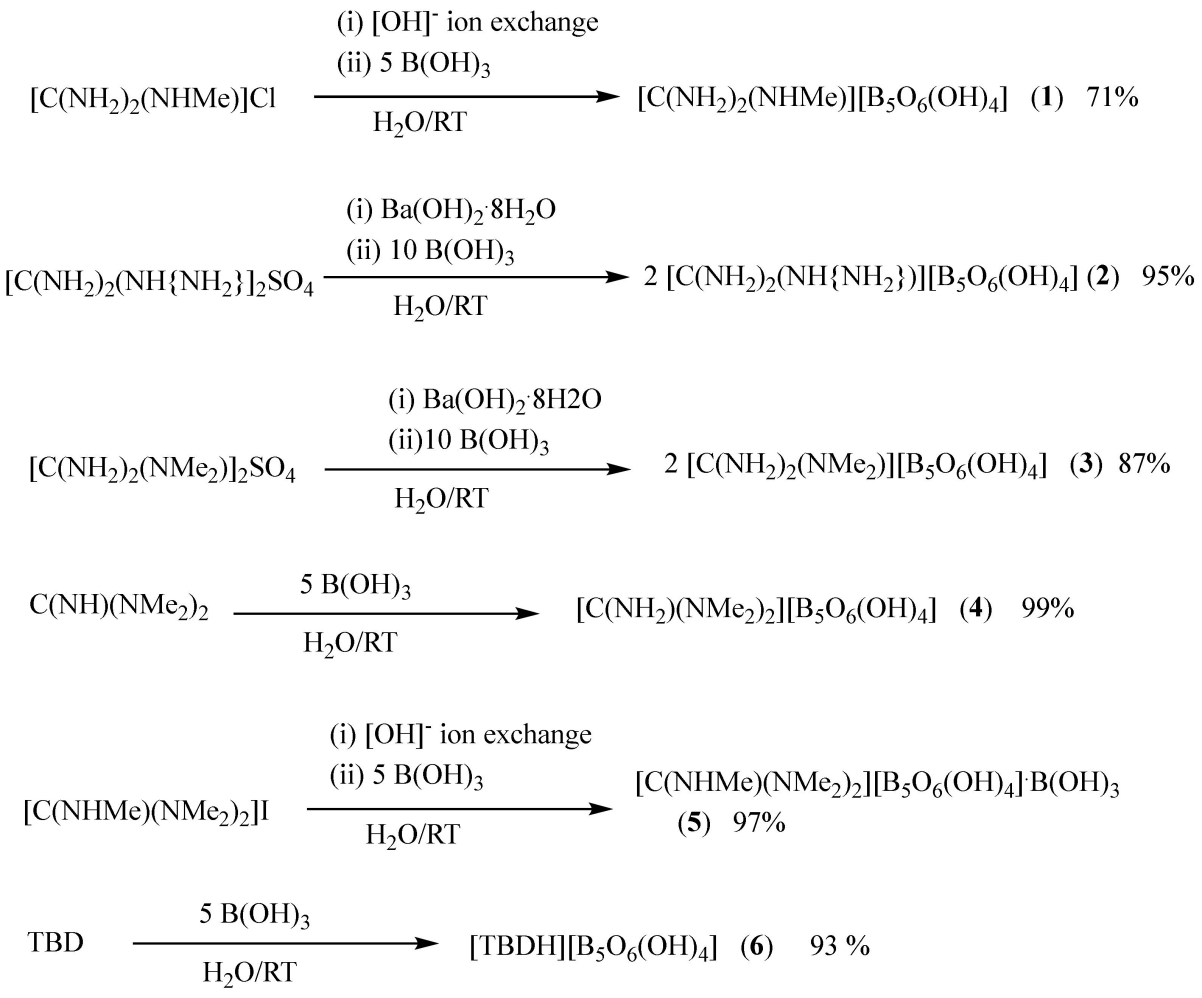
Synthetic procedure for the synthesis of compounds **1**–**6** (TBD = 1,5,7-triazabicyclo[4.4.0]dec-5-ene and the structure of TBDH is illustrated in Figure 1f).

The *N*-substituted guanidinium starting materials used in this study were all commercially available and were non-carbonate species. We have previously synthesized many nonmetal cation polyborate salts by a room temperature crystallization of aqueous solutions originally primed with an organic free base, or its protonated cation (prepared in situ as its [OH]^−^ salt by a metathesis reaction) and B(OH)_3_ [10,11]. Based on this strategy we prepared six new *N*-substituted guanidinium salts as shown in Scheme 1. Crude yields of these compounds ranged from 71% to near quantitative. The recrystallization of samples from H_2_O gave crystals suitable for single-crystal X-ray diffraction studies.

Thermal and spectroscopic data (Section 2.2) and elemental analysis data (Section 3.3, Section 3.4, Section 3.5, Section 3.6, Section 3.7, Section 3.8 and Section 3.9) on the crude products **1**–**6** were consistent with formulating these materials as *N*-substituted guanidinium pentaborate salts and these formulations were confirmed by single-crystal XRD studies (Section 2.3). All compounds were colourless and stable in the solid state, insoluble in organic solvents but soluble in H_2_O with decomposition.

### 2.2. Thermal and Spectroscopic Properties

Thermal gravimetric analysis (TGA) data (in air) for nonmetal cation polyborate salts are often reported with a thermal decomposition leading to B_2_O_3_ via multistage processes involving the loss of interstitial H_2_O (100–200 °C), the condensation of hydroxy groups bound to boron with the loss of a further two H_2_O molecules (250–400 °C), and then the oxidation of organics (400–700 °C) [10,31,32]. A TGA was undertaken on compounds **1**–**3** and **6** as representative examples of the new substituted guanidinium pentaborate(1-) salts. Compounds **2**, **3**, and **6** did not have interstitial H_2_O and their TGA curves showed the expected loss of two H_2_O molecules between 240 and 320 °C. Compound **1** followed this expected behaviour but there was no clear distinction between the two lower temperature processes that occurred (100–275 °C) with the loss of three H_2_O molecules. The glassy solids that remained after heating to 700 °C for compounds **1**–**3** and **6** had residual masses consistent with 2.5 B_2_O_3_.

^1^H and ^13^C NMR were obtained for compounds **1** and **3**–**6** dissolved in D_2_O and these all gave spectra consistent with the appropriate guanidinium cations being present, e.g., the ^1^H spectrum of compound **4** gave a signal for the *N*-Me groups as a singlet at 2.85 ppm with exchangeable H atoms with HOD (4.7 ppm). The ^13^C{^1^H} spectrum of compound **4** gave two signals at 36.98 and 160.2 ppm for the *C*H_3_ and *C*N_3_ carbons, respectively, with the downfield signal being weak. The presence of boron in these compounds was confirmed by ^11^B NMR (in D_2_O) for all compounds. All ^11^B spectra all showed three “signature” signals [31,33], arising through the decomposition of a pentaborate, with relative intensities appropriate for a sample that had attained polyborate/aqueous equilibrium [27]. Thus, compound **4** gave three signals at 1.0, 12.8, and 19.1 ppm with approximate relative intensities of 5%, 10%, and 85%, respectively. These signals have been previously assigned, moving downfield, to [B_5_O_6_(OH)_4_]^−^ (tetrahedral *B*), [B_3_O_4_(OH)_4_]^−^, and B(OH)_3_/[B(OH)_4_]^−^ [27]. Three signals were observed as the sample was relatively concentrated rather than one signal at +16.1, which would have been expected at infinite dilution [33].

All samples were characterized by FTIR spectroscopy. Strong and potentially diagnostic absorptions were to be expected for the cation ν(NH) at ca. 3400 cm^−1^ and ν(CN) at ca. 1650 cm^−1^ [34] and the polyborate anion ν(O-H) at 3500 cm^−1^ and the ν(B-O) stretches grouped between 1450 and 740 cm^−1^ [35]. In particular, a strong adsorption at ca. 925 cm^−1^ in the (B_trig_-O)_sym_ stretching region has previously been described as diagnostic for the [B_5_O_6_(OH)_4_]^−^ anion [36]. This strong absorption, amongst other strong B-O stretches, together with the strong adsorptions associated with the cation (1671–1625 cm^−1^), was present in all samples.

### 2.3. Single-Crystal XRD Studies

**Figure 2:**
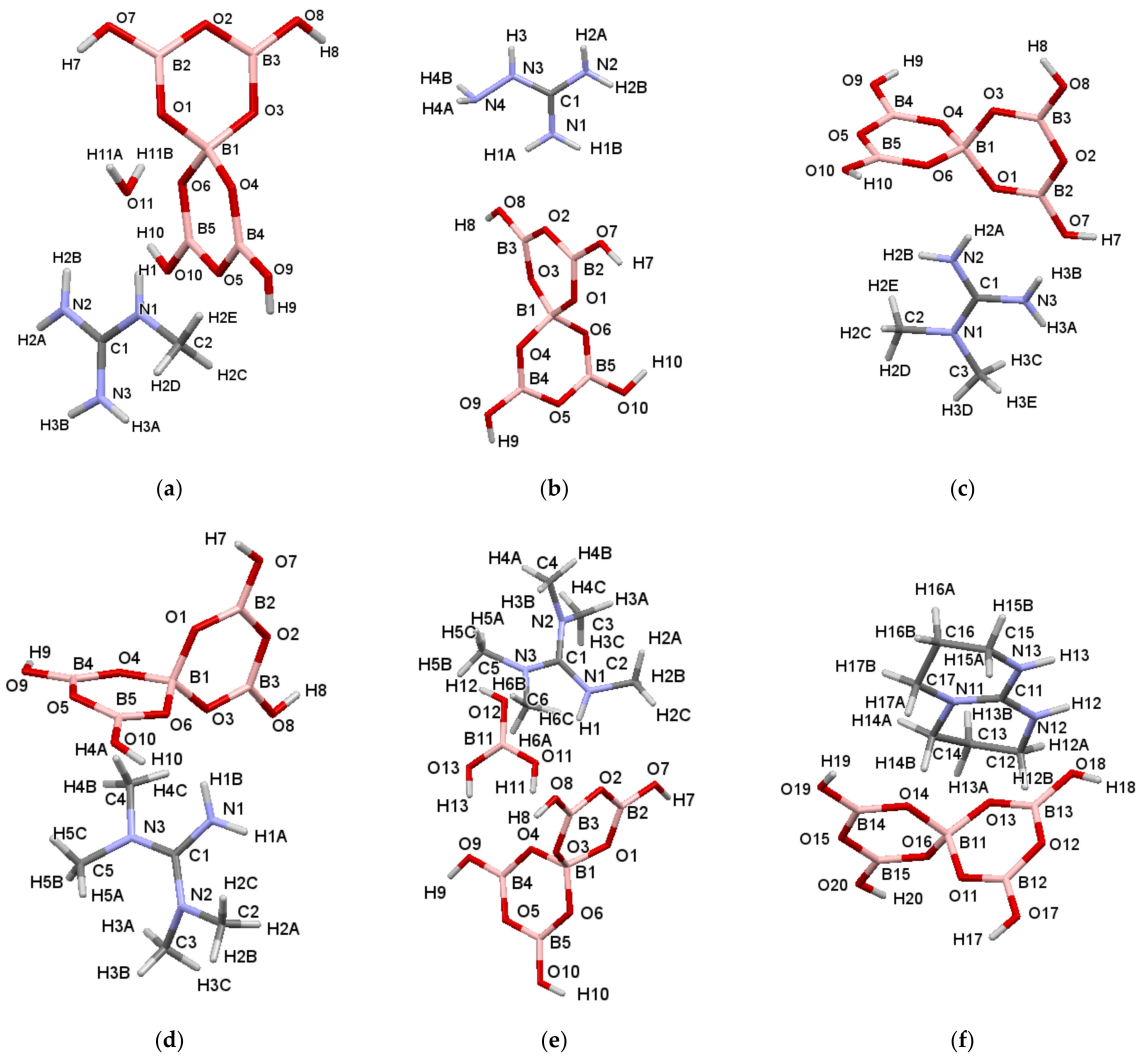
Drawings of the crystals structures of (**a**) [C(NH_2_)_2_(NHMe)][B_5_O_6_(OH)_4_]·H_2_O (**1**), (**b**) [C(NH_2_)_2_(NH{NH_2_})][B_5_O_6_(OH)_4_] (**2**), (**c**) [C(NH_2_)_2_(NMe_2_)][B_5_O_6_(OH)_4_] (**3**), (**d**) [C(NH_2_)(NMe_2_)_2_][B_5_O_6_(OH)_4_] (**4**), (**e**) [C(NHMe)(NMe_2_)_2_][B_5_O_6_(OH)_4_]·B(OH)_3_ (**5**), and (**f**) [TBDH][B_5_O_6_(OH)_4_] (**6**) (TBD = 1,5,7-triazabicyclo[4.4.0]dec-5-ene) showing atomic numbering. Compound **6** is modulated and just one component of the disordered anion and cation is shown in (**f**).

Compounds **1**–**6** were characterized by single-crystal XRD studies. All the compounds contained the expected insular *N*-substituted guanidinium(1+) cations and insular tetrahydroxidohexaoxidopentaborate(1-) anions within the asymmetric unit. Additionally, compound **1** had one H_2_O of crystallization per cation/anion and compound **5** was cocrystallized with a molecule of B(OH)_3_ per cation/anion. Crystallographically, compounds **1**–**5** were free of disorder, whilst compound **6** was modulated, but was only refined using the subcell with all atoms disordered over two positions of equal population. Brief crystallographic details for each compound are given in the experimental section, and atomic numbering schemes for compounds **1**–**6** are shown in Figure 2. The full crystallographic information is available as Supplementary Materials. Generally, the gross structures, bond angles, and bond distances within the guanidinium [17,37] and pentaborate [31,32,33,36] units were within the expected ranges for these ions and need no further comment. The *N*-substituted guanidinium cations had a variable number of potential H-bond donor sites (one to seven in this study) that were dependent on the extent of the substitution. Each pentaborate(1-) unit had four potential H-bonds donor sites and ten potential H-bond acceptor sites in locations described as α, β, or γ, as defined elsewhere [10,32], and they are illustrated in Figure 1. Since solid-state H-bonding is likely to be important in stabilizing these structures [23,24,25,26], the H-bond interactions in compounds **1**–**6** were analysed and are described in detail using an Etter graph set terminology [38].

Compounds **2**, **3**, **4**, and **6** had the extended anion–anion H-bonded lattices that are found in many nonmetal cation pentaborate salts [10], with the cations filling the “voids” and “channels” and forming additional H-bonds to the anions. In these structures, each pentaborate(1-) anion donates four H-bond to four neighbouring pentaborate(1-) anions. The extended lattice structures are 3-D because the central B atom in each pentaborate(1-) is tetrahedral with its two associated boroxole rings perpendicular to each other (*D*_2*d*_ for heavy atoms).

Thus, in compound **4**, the H-bonds originating from each pentaborate were to three α and one β (or α,α,α,β) acceptor sites of its neighbours (see Figure 1 for acceptor site positions). This particular anion–anion giant structure is commonly found in many nonmetal cation pentaborate salts [10] and has be described as a “brick wall” with three R_2_^2^(8) [38] reciprocal-α interactions forming a “layer”, resembling a brick wall, and the fourth interaction, at a β-site, linking these layers by infinite C(8) [38] chains. These R_2_^2^(8) reciprocal interactions are known to be strong and are particularly common in polyborate chemistry [10,31,32,33,36,39]. R_2_^2^(8) interactions are also prevalent elsewhere, e.g., carboxylic acid and boronic acid dimers [26]. In compound **4** these donors were O7-H7, O9-H9, and O10-H10 and the acceptors were O1, O4, and O6, respectively, on three different neighbouring pentaborate anions. The C(8) chain that linked layers arose through O8H8⋯O10′. The [C(NH_2_)(NMe_2_)_2_]^+^ cations in compound **4** were in the channels of the brick-wall structure (when viewed along the *b* axis) and each formed two donor H-bonds from the amino group to the γ (N1H1A⋯O2) and α (N1H1B⋯O3) positions of two neighbouring pentaborate(1-) anions.

**Figure 3:**
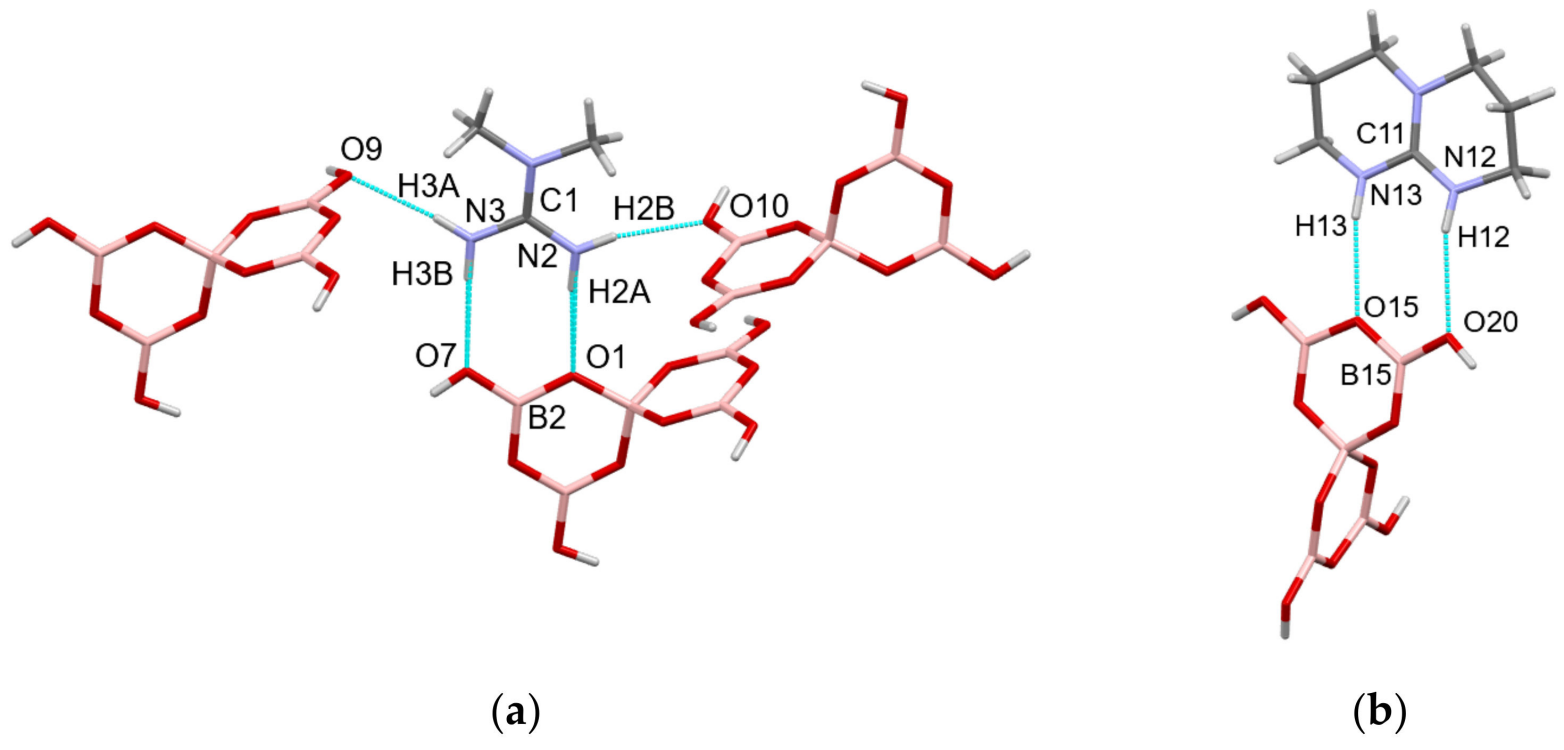
H-bonds originating from the *N*-substituted guanidinium centres present in compounds (**a**) **2**, and (**b**) **6**, illustrating the “pincer”-like R_2_^2^(8) rings.

Compounds **2** and **3** had similar, and previously observed [10], anion–anion H-bond arrangements (α,α,α,γ acceptor sites) as three R_2_^2^(8) reciprocal-α interactions and one R_2_^2^(8) reciprocal-γ interaction. The reciprocal-α H-bond donor interactions originated from O7-H7, O8-H8, and O10-H10 in compound **2** and from O8-H8, O9-H9, and O10-H10 in compound **3**. The reciprocal-γ interactions for compound **2** were -O9-H9⋯O5′-B4′-O9′-H9′⋯O5-B4- and those for compound **3** were -O7-H7⋯O2′-B2′-O7′-H7′⋯O2-B2-. The [C(NH_2_)_2_(NMe_2_)]^+^ cation in compound **3** formed four H-bonds to three pentaborates of the anionic lattice. These interactions are shown in Figure 3a. The two interactions starting at N2-H2B and N3-H3A were both part of a dimeric centrosymmetric R_4_^4^(20) ring comprised of two anions and two cations. The H-bonds originating from N2-H2A and N3-H3B were also of interest since these amino-hydrogens participated in a charge-assisted pincer R_2_^2^(8) ring [23,40], as a double donor to an α-site (O1) and a β-site (O7) of a single pentaborate(1-) anion. Each [C(NH_2_)_2_(NH{NH_2_})]^+^ cation in compound **2** had seven potential H-bond donor sites and all were involved in H-bonds to five neighbouring pentaborate anions in a complex manner. However, the H-bond motifs described for compound **3** were also readily discerned in compound **2**. Thus, “simple” interactions involved N1-H1A and N4-H4A with O8 (β) and O4 (α), respectively. N4-H4B was involved in a bifurcated H-bond bridging two pentaborates at the O5 (γ) sites. The four remaining H-bond donors, N1-H1B, N2-H2B, N2-H2A, and N3-H3, participated in two pincer-type R_2_^2^(8) rings to two neighbouring pentaborates at O9 (β)/O4 (α) and O2 (γ)/O7 (β), respectively.

Compound **6** also had a previously observed anion–anion giant lattice with α,α,α,β acceptor sites (also observed in compound **4**) but now arranged as three reciprocal-α rings and one R_2_^2^(12) reciprocal-β ring [10]. The reciprocal-α H-bonds donor originated from O17-H17, O19-H19, and O20-H20 and the R_2_^2^(12) reciprocal-β ring involved O18-H18⋯O17′ and O18′-H18′⋯O17. The bicyclic [TBDH]^+^ cation in compound **6** had two H-bonds donor sites, N12-H12 and N13-H13. Both were involved in a pincer-type R_2_^2^(8) ring at O20 (β) and O15 (γ) acceptor sites, respectively, of a single pentaborate anion, Figure 3b.

Compounds **1** and **5** had additional cocrystallized molecules with the cation and anion. Cocrystallized molecules in H-bonded anionic polyborate lattices usually function either by (i) helping to fill space within the areas where the cations sit or by (ii) expanding the anionic lattices to help accommodate larger cations [10,41].

Compound **1** had a pentaborate lattice structure that was very similar to compound **3** with α,α,α,γ acceptor sites involved in four R_2_^2^(8) rings. In compound **1**, these donors were O7-H7, O8-H8, O9-H9, and O10-H10, and the acceptors were O3 (α), O1 (α), O5 (γ), and O6 (α) on four different neighbouring pentaborate anions. The structure of compound **1** differed from that of compound **3** in the detail of their H-bonded R_2_^2^(8) rings with H-bonds originating from O10-H10 and O9-H9 reciprocal and those originating from O7-H7 and O8-H8 paired. The [C(NH_2_)_2_(NHMe)]^+^ cation in compound **1** had five H-bond donor sites. N2-H2A and N3-H3B were both part of a pincer-like R_2_^2^(8) ring to O4 (α) and O9 (β) acceptor sites, respectively. N3-H3A and N2-H2B both donated H-bonds to a β-sites (O7 and O8). The H-bond from N2-H2B had a N2-H2B-O8 angle of 141.1(13)° and a H2B⋯O8 distance of 2.322(15) Å. The H-bond arising from N2-H2B may be considered as bifurcated since O11 (H_2_O) had a N2-H2B-O11 angle of 138.8(13)° with a long, potentially H-bonding H2B⋯O11 distance of 2.661(15) Å. This H_2_O molecule was also involved with closer and stronger acceptor interaction from N1-H1 (H1⋯O11, 1.943(15) Å and an angle of N1-H1⋯O11 170.2(14)°. This interaction together with that from N2-H2B formed a pincer R_2_^1^(6) ring. The H_2_O molecule formed two donor bonds to two pentaborates at α,α (O1/O4, bifurcated) and β (O10) sites. The anion–anion lattice in compound **1** was largely unaffected by the presence of the cocrystallized H_2_O molecule. Since the cation in compound **1** was relatively small, the H_2_O positioned itself to fill space within the lattice reserved for the cations and further stabilized the structure by these H-bond interactions.

**Figure 4:**
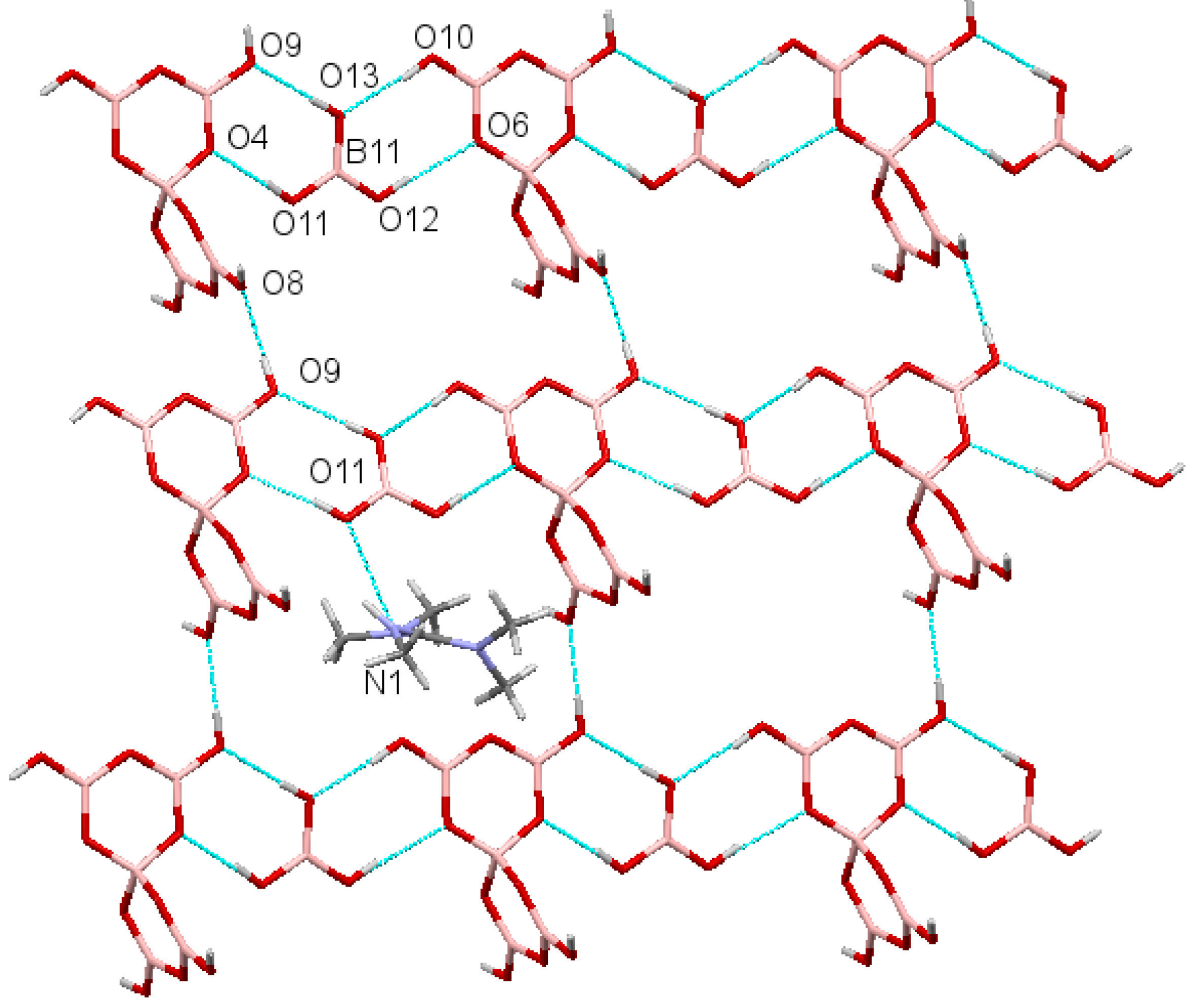
In [C(NHMe)(NMe_2_)_2_][B_5_O_6_(OH)_4_]·B(OH)_3_ (**5**) the “horizontal” chains (three partially shown) of alternating H-bonded R_2_^2^(8) pentaborate(1-)/boric acid units are cross-linked by H-bonded C(8) “vertical” chains (O9H9⋯O8′) into planes. For clarity, H atoms are not labelled but are numbered the same as the oxygen/nitrogen atom to which they are attached. The [C(NHMe)(NMe_2_)_2_]^+^ cations (one shown) fill the “voids” and are H-bonded to the B(OH)_3_ units (N1H1⋯O11).

Compound **5** had B(OH)_3_ cocrystallized with the pentaborate salt and consequentially had a unique anionic pentaborate/boric acid lattice. However, the R_2_^2^(8) building motifs seen in most polyborate structures were also readily identified here. Thus, one “plane” was comprised of chains of pentaborates each linked via two reciprocal-α rings. These reciprocal-α interactions originated from O7-H7 and O8-H8 with O3 and O1 acceptors, respectively. The other two H-bond interactions originating from each pentaborate (O9-H9 and O10-H10) are shown in Figure 4. The interactions shown here were approximately perpendicular to those just described an originating from O7-H7 and O-H8. The “horizontal” chains in Figure 4 were comprised of alternating pentaborate(1-)/boric acid moieties with the H-atom positions of the B(OH)_3_ acid arranged to maximise R_2_^2^(8) interactions. Thus, O10-H10 on the pentaborate and O12-H12 on the B(OH)_3_ were involved in a standard borate–borate interaction and O11-H11 and O13-H13 were involved in a “pincer” double H-bond to the pentaborate at O4 (α) and O9 (β) sites. Each B(OH)_3_ formed three donor H-bonds to two α sites and one β site of neighbouring pentaborates. The fourth and final H-bond originating from each pentaborate is also shown in Figure 4. Here, O9-H9 formed an H-bond to a β-acceptor site (O8) of its neighbour, cross-linking the pentaborate(1-)/boric acid chains into the “plane” shown in Figure 4 by the formation of C(8) chains. The sterically demanding [C(NHMe)(NMe_2_)_2_]^+^ cation in compound **5** had only one amino group (N1-H1) available for H-bonding and this was utilized in the H-bonding to O11 of the B(OH)_3_ (Figure 4). The B(OH)_3_ in this structure served to expand the anionic pentaborate lattice to enable the sterically demanding cation more space. This behaviour has been observed before in several polyborate [36,41,42,43] salts including [N(*n*Pr)_4_][B_5_O_6_(OH)_4_][B(OH)_3_]_2_ and [HPS][B_5_O_6_(OH)_4_]^.^B(OH)_3_ (PS = proton sponge, 1,8-bis(dimethylamino)naphthalene).

### 2.4. Discussion on H-Bonding in Guanidinium Polyborates

The anions in polyborate salts, [B*_a_*O*_b_*(OH)*_c_*]*^n^*^−^, are invariably rich in H-bond donor sites (there are *c* donor BOH groups) and oxygen-atom H-bond acceptor sites with acceptor sites (*b* + *c*) outnumbering the donor sites and enabling the opportunity for H-bond donor cations to contribute to the H-bonding networks. R_2_^2^(8) are important stabilizing interactions [10,33] and the *maximum* number of borate–borate R_2_^2^(8) rings available to a polyborate anion is limited to the lower value of *b* or *c*, since bridging oxygen atoms are acceptors in these R_2_^2^(8) interactions. In actual structures, packing/steric effects may reduce further this maximum number.

The guanidinium (G) cation is well-known as a multi-H-bond donor in solid-state structures and several H-bond motifs are commonly observed [23,24,37,44,45,46,47,48]. These include a donor H-bond to an electronegative atom from a single N-H site (often this simple motif is incorporated into larger more complex rings and/or chains), and two pincer-type doner ring motifs to either a single acceptor site, R_2_^1^(6), or to two acceptor sites, R_2_^2^(8). The structure of GCl has Cl^−^ ions bridging G cations as part of alternating anion–cation chains linked by H-bonded R_2_^1^(6) rings [44]. Oxoanions (e.g., borate esters [24], carboxylate [45,46], sulfonates [47,48], phosphonates [48]), are good R_2_^2^(8) pincer H-bond acceptors and these interactions are strong and have been described as “‘charge-assisted” [45,49]. The structure of G[RSO_3_] is self-assembled and templated into layers with each G cation forming three such pincer R_2_^2^(8) rings with three organosulfonate anions [47]. Cubic hydrogen-bonded borate networks containing four pincer R_2_^2^(8) rings per borate have been observed in G_4_[B(OMe)_4_]_3_Cl·5H_2_O [24].

As noted in the introduction, the G cation was found in three polyborate structures. These “oxoanions” are ideally set up to accept the pincer R_2_^2^(8) rings, and we examined, focussing on the polyborate anion, the extent of such interactions as they are in direct competition (see below) with borate–borate R_2_^2^(8) rings in stabilizing these structures. We also examined how *N*-substitution affected this R_2_^2^(8) balance and how this affected the observed structures.

When considering solid-state H-bonding interactions the generalised guanidinium (either *N*-substituted or unsubstituted) polyborate salt can be represented as (*x*G)*_n_*[B*_a_*O*_b_*(OH)*_c_*] where *x* represents the *maximum* number of potential donor pincer R_2_^2^(8) motifs available to the cation. Each unsubstituted G cation has the potential to form a maximum of three donor pincer R_2_^2^(8) motifs (i.e., *x* = 3), whilst substituted G cations will have fewer opportunities (*x* = 0, 1, or 2) with the value of *x* dependent on the extent and position of the substitution. Within guanidinium polyborate salts, the maximum number of R_2_^2^(8) acceptor interactions (both pincer and normal) is *b*, since bridging oxygen atoms generally can only be used once and are required for both types of these R_2_^2^(8) interactions. Usually, borate–borate interactions are approximately coplanar with boroxole rings. Packing/steric effects may reduce the maximum number for both types of interactions.

This analysis focused on an analysis of two ratios. Firstly, a value of >1 for (*x*G*n* + *c*)/*b* would indicate borate–borate and pincer R_2_^2^(8) motifs were in direct competition for the available borate acceptor sites. Secondly, potential and actual (i.e., observed) (*x*G*n*)/*c* ratios for each compound were also of interest since a difference in these values may indicate a structural preference for one or the other of these H-bonding motifs.

Using this approach, G_2_B_4_O_5_(OH)_4_, GB_5_O_6_(OH)_4_, and G_3_B_9_O_12_(OH)_6_, had (*x*G*n* + *c*)/*b* ratios of 2.0, 1.17, and 1.25, respectively, confirming that pincer/borate donor rings were in competition for the available acceptor sites. The potential donor ratios, (*x*G*n*)/*c*, for the three compounds were 1.5, 0.75, and 1.5.

The examination of the structure of G_2_[B_4_O_5_(OH)_4_]·2H_2_O [17] revealed two independent G cations. Two molecules of H_2_O within this formula unit also affected the structure but did not affect this analysis. All five bridging oxygen atoms of the tetraborate(2-) anion were involved in R_2_^2^(8) rings with four cation–borate pincer interactions and only one borate–borate interaction. Thus, within this structure, 80% of these rings involved G cations and the observed (*x*G*n*)/*c* ratio was 4.0. This value was much higher than would be expected (1.5) based on the number of potential donor sites. The higher-than-expected observed (*x*G*n*)/*c* ratio would indicate that the G cations were either able to replace the strong borate–borate interactions with stronger pincer motifs or that additional factors were also important. Such factors may include the compound’s stoichiometry and that the three-fold symmetry of G cations (*D_3d_*) were a good packing match for the tetraborate(2-) anion (*C_2v_*). H-bonding in tetraborate(2-) structures not containing the G cation have been recently reviewed [50] and their structural architectures are, as expected, dominated by borate–borate interactions with the majority of structures having at least two R_2_^2^(8) rings.

Within the structure of G_3_[B_9_O_12_(OH)_6_] [19], all twelve bridging oxygen acceptor sites were involved in eleven R_2_^2^(8) H-bonded rings with seven pincer charge-assisted interactions involving the three G cations and four borate–borate interactions. Interestingly, the four bridging oxygen atoms joined to the central boron were involved, solely amongst themselves, in three pincer rings; the two oxygens that bridged two four-coordinate borons were both involved in two pincer rings. These rings were not coplanar with boroxole rings. In this salt, 64% of the R_2_^2^(8) rings involved the G cations and 36% were borate–borate. This observed ratio of 1.78 was higher than that expected (1.5) based on total potential donor sites and would again indicate that strong cation–anion pincer interactions were able to replace the strong borate–borate interactions, but again, stoichiometry and packing considerations were also important.

The H-bonded structure of G[B_5_O_6_(OH)_4_]·H_2_O [18] utilizes only four of the six available acceptor bridging oxygen atoms in R_2_^2^(8) interactions. Here, and in contrast to the tetraborate(2-) and nonaborate(3-) structures, 75% of the H-bond ring interactions were borate–borate R_2_^2^(8) rings with the charge-assisted pincer rings only accounting for 25%. The observed (*x*G*n*)/*c* ratio of 0.33 was lower than would be expected (0.75) from the number of potential donor sites and confirmed that the borate–borate interactions strongly influenced that structure and that the templating influence of G was minimal. These structures were strongly stabilized by these anion–anion interactions with any additional cation–anion interactions helping to further stabilize the anionic lattice [10]. Many nonmetal cation pentaborate(1-) salts show three such borate–borate interactions [10]. This was also the likely situation in this salt since the three-fold symmetry of the cation did not pack well with the *D*_2*d*_ symmetry of the insular pentaborate(1-) anion and its perpendicularly H-bonded giant lattice.

Adding substituents to the G cations affects their structure directing potential in three ways: it reduces their ability to form H-bonds, increases their steric bulk, and disrupts their three-fold symmetry. The first factor also correspondingly lowers *x*, reduces the (*x*G*n*)/*c* ratio, and hence encourages more borate–borate H-bonds. The structures of the *N*-substituted guanidinium cation pentaborate(1-) salts **1**–**6** were described in detail in Section 2.2, but further general comments based on the analysis above would now be appropriate. The numbers of potential pincer H-bond donor sites (x) available in compounds **1**–**6** were 2, 2, 1, 0, 0, and 1, and the (*x*G*n*)/*c* ratios were lower than that for G[B_5_O_6_(OH)_4_] (0.75) at 0.5, 0.5, 0.25, 0, 0, and 0.25, respectively. This analysis would indicate borate–borate interactions should dominate the H-bond interactions in compounds **1**–**6**, with additional charge-assisted pincer H-bonds forming wherever possible. Unsurprisingly, three or four borate–borate R_2_^2^(8) rings were observed in compounds **1**–**6** and these interactions were clearly important in stabilizing their solid-state structures. Pincer interactions were unavailable for compounds **4** and **5** and the amino-H atoms formed simple H-bonds to borate/boric acid acceptor sites. Compounds **3** and **6** both had the potential to form one pincer bond to stabilize the lattice and each had one such pincer bond. Compound **2** had the potential to form two pincer bonds, and both were formed. Compound **1** had potentially two pincer bonds to the borate but here, only one was observed. However, this was a direct consequence of the cocrystallized H_2_O since the H_2_O was part of an alternative R_2_^1^(6) pincer ring motif. Compound **5** had a molecule of B(OH)_3_ cocrystallized and no opportunity to form a pincer ring. As noted in Section 2.2, the [C(NHMe)(NMe_2_)_2_]^+^ in compound **5** was relatively bulky and the B(OH)_3_ served to extend the anionic lattice, by positioning itself between two pentaborate(1-) anions. It is interesting to note that the H-atom positions in B(OH)_3_ were asymmetric (see Figure 4) and therefore allowed it to partake in an R_2_^2^(8) donor pincer interaction to one pentaborate(1-) and a standard R_2_^2^(8) borate interaction to the other.

## 3. Materials and Experimental Methods

### 3.1. General

Reagents were all obtained commercially. FTIR spectra were obtained as KBr pellets on a Perkin-Elmer 100FTIR spectrometer (Perkin-Elmer, Seer Green, UK). ^1^H, ^11^B, and ^13^C{^1^H} NMR spectra were obtained on a Bruker Ultrashield Plus 400 spectrometer (Bruker, Coventry, UK) on samples dissolved in D_2_O at 400, 128, and 100 MHz, respectively. Chemical shifts are in ppm with positive values to the high frequency (downfield) of TMS (^1^H, ^13^C) or BF_3_^.^OEt_2_ (^11^B). TGA and DSC were performed on an SDT Q600 V4 instrument (TA Instruments, New Castle, DE, USA) using Al_2_O_3_ crucibles with a temperature ramp-rate of 10 °C per minute (20–700 °C in air). X-ray crystallography was performed at the EPSRC national crystallography service centre at Southampton University. Drawings in this manuscript have been generated using Mercury software (CCDC, Cambridge, UK). CHN analyses were obtained from OEA Laboratories (Callingham, Cornwall, UK).

### 3.2. X-ray Crystallography

Crystallographic data for compounds **1**–**3**, **5**, and **6** were obtained, with crystals kept at *T* = 100(2) K during data collection, on either a Rigaku FRE+ diffractometer equipped with VHF Varimax confocal mirrors and an AFC12 goniometer and Hypix 6000 detector (**1**, **3**) or a Rigaku 007HF equipped with HF Varimax confocal mirrors and an AFC11 goniometer and HyPix 6000 detector (**2**, **5**, **6**) using CrysAlisPro [51]. Structures (**1**–**3**, **5**, **6**) were solved with the ShelXT [52] structure solution programme using the dual-solution structure method (**1**, **5**) or the intrinsic-phasing solution method (**2, 3**, **6**).The crystallographic data collection for compound **4** was obtained at 120(2) K on a Bruker-Nonius FR591 diffractometer with a *Nonius KappaCCD* detector using Collect [53] and Denzo [54] and *SHELXS97* [55] was used for the structure solution. By using Olex2 [56] as the graphical interface, all structure models were refined with ShelXL [57] using a least-squares minimisation. Structure refinement details and ORTEP diagrams with 50% probability displacement ellipsoids for compounds **1**–**6** are given in the Supplementary Materials and selected crystallographic data are given for each compound in Section 3.3, Section 3.4, Section 3.5, Section 3.6, Section 3.7, Section 3.8 and Section 3.9.

### 3.3. Preparation of [C(NH_2_)_2_(NHMe)][B_5_O_6_(OH)_4_]·H_2_O (***1***)

DOWEX 550A (OH)^−^ ion exchange resin (18 g) was added to a solution of *[C(NH_2_)_2_(NHMe)]Cl* (0.5 g, 4.6 mmol) in H_2_O (25 mL) and the mixture was stirred for 18 h. The resin was removed by filtration and B(OH)_3_ (1.4 g, 23 mmol) was added to the filtrate. The solution was stirred for a further 2 h, evaporated under reduced pressure to yield 1.1 g of a white powder as a crude product (71%). Recrystallization of 0.3 g of the product from warm H_2_O afforded a few crystals suitable for XRD studies. TGA: 100–275 °C, condensation of pentaborate with loss of 3 H_2_O 17.4% (17.4% calc.); 275–700 °C, oxidation of organic residue leaving residual 2.5 B_2_O_3_ 55.9% (56.1% calc.). NMR/ppm: δ_H_: 2.69 (s, CH_3_) 4.70 (HOD); δ_B_: 1.1 (3%), 13.2 (24%), 18.1 (73%); δ_C_: 36.98 (*C*H_3_). FTIR (KBr, cm^−1^): 3435(s), 3400(s), 2958(m), 2924(m), 1664(s), 1439(m), 1347(m), 1103(m), 1025(m), 925(s), 782(m), 696(m). XRD crystallographic data: C_2_H_14_B_5_N_3_O_11_, *M_r_* = 310.21, monoclinic, *P*2_1_/*c* (no. 14), *a* = 9.9962(2) Å, *b* = 10.9047(2) Å, *c* = 11.7215(2) Å, *β* = 96.101(2), *α* = *γ* = 90°, *V* = 1270.47(4) Å^3^, *T* = 100(2) K, *Z* = 4, *Z*′ = 1; 17,592 reflections were measured, 2893 unique (*R_int_* = 0.0115), which were used in all calculations. The final *wR_2_* was 0.0743 (all data) and *R_1_* was 0.0267 (I > 2σ(I)).

### 3.4. Preparation of [C(NH_2_)_2_(NH{NH_2_})][B_5_O_6_(OH)_4_] (***2***)

[C(NH_2_)_2_(NH{NH_2_})]_2_SO_4_ (1 g, 4.0 mol) was dissolved in H_2_O (25 mL) and to this was added Ba(OH)_2_^.^8H_2_O (1.3 g, 4.1 mmol) in H_2_O (5 mL). The resulting cloudy solution was stirred for 1 h and the solid barium sulphate was filtered from the solution. B(OH)_3_ (2.3 g, 37 mmol) was added to the clear solution, and stirred with gentle heating for 3 h. The solution was then evaporated under reduced pressure and dried in an oven to afford 2.0 g of a white powder as crude product (95%). A sample (0.3 g) of this solid was redissolved in 10 mL deionised water and left to crystallise over a few days to yield a few crystals suitable for XRD studies. CH_11_B_5_N_4_O_10_. Anal. Calc.: C = 4.1%, H = 3.8%, N = 19.1%. Found: C = 4.2%, H = 3.7%, N = 18.7%. TGA: 240–320 °C, condensation of pentaborate(1-) anions with loss of 2 H_2_O 11.9% (12.3% calc.); 320–700 °C, oxidation of organic residue leaving residual 2.5 B_2_O_3_ 59.3% (59.3% calc.). NMR/ppm: δ_B_: 1.2 (10%), 13.3 (9%), 19.2 (81%). FTIR (KBr, cm^−1^) 3451(s), 3372(s), 3295(s), 3235(m), 1672(s), 1657(s), 1437(s), 1361(s), 1252(m), 1196(m), 1103(s), 1026(s), 925(s), 782(s), 698(s). XRD crystallographic data: CH_11_B_5_N_4_O_10_, *M_r_* = 293.19, triclinic, *P*-1 (no. 2), a = 7.4870(2) Å, b = 8.5076(2) Å, c = 9.6502(2) Å, *α* = 93.906(2)°, *β* = 98.470(2)°, *γ* = 96.457(2)°, *V* = 601.88(3) Å^3^, *T* = 100(2) K, *Z* = 2, *Z*′ = 1; 11,885 reflections were measured, 2142 unique (*R_int_* = 0.0404), which were used in all calculations. The final *wR_2_* was 0.0827 (all data) and *R_1_* was 0.0294 (I > 2σ(I)).

### 3.5. Preparation of [C(NH_2_)_2_(NMe_2_)][B_5_O_6_(OH)_4_] (***3***)

An aqueous solution (5 mL) containing Ba(OH)_2_·8H_2_O (1.2 g, 3.8 mmol) was added to an aqueous solution (25 mL) of [C(NH_2_)_2_(NMe_2_)]_2_SO_4_ (1.0 g, 3.7 mmol). The mixture was stirred for 1 h and the BaSO_4_ was removed from the solution by gravity filtration and washed with H_2_O. B(OH)_3_ (2.2 g, 36 mmol) was added to the filtrate and the solution was stirred under gentle warming for 1 h. The solvent was removed under reduced pressure to yield 2.0 g of a white powder as the crude product (87%). This solid was characterised by FT-IR and NMR (^1^H, ^11^B, ^13^C) studies. A sample (0.3 g) of the crude product was redissolved in warm H_2_O and left to recrystallize. A few white single crystals suitable for XRD studies were obtained. C_3_H_14_B_5_N_3_O_10_. Anal. Calc.: C = 11.8%, H = 4.6%, N = 13.8%. Found: C = 11.9%, H = 4.6%, N = 13.5%. TGA: 240–275 °C, condensation of pentaborate(1-) anions with loss of 2 H_2_O 12.3% (11.8% calc.); 275–700 °C, oxidation of organic residue leaving residual 2.5 B_2_O_3_ 55.3 (56.8% calc.). NMR/ppm δ_H_: 2.89 (s, CH_3_N), 4.70 (HOD); δ_B_: 1.0 (13%), 13.2 (11%), 18.3 (77%); δ_C_: 37.34 (*C*H_3_). FTIR (KBr, cm^−1^): 3370(s), 3249(m), 3200(m), 1688(m), 1671(s), 1634(s), 1435(vs), 1393(s), 136 (s), 1313(s), 1114(s), 1033(s), 926(vs), 780(s), 699(m). XRD crystallographic data: C_3_H_14_B_5_N_3_O_10_, *M*r = 306.22, monoclinic, P2_1/c_, *a* = 9.9747(2) Å, *b* = 11.2563(3) Å, *c* = 11.6174(3) Å, *α* = *γ* = 90°, *β* = 96.084(2)°, *V* = 1297.03(5) Å^3^, *T* = 100(2) K, *Z* = 4, *Z*′ = 1; 18,168 reflections were measured, 2969 unique (*R_int_* = 0.0191), which were used in calculations. The final *ωR_2_* was 0.0811 (all data) and *R_1_* was 0.0280 (I > 2σ(I)).

### 3.6. XRD Data for [C(NH_2_)(NMe_2_)_2_][B_5_O_6_(OH)_4_] (***4***)

B(OH)_3_ (6.2 g, 100 mmol) was dissolved in H_2_O (100 mL) and [C(NH)(NMe_2_)_2_] (1.2 g, 20 mmol) was added with stirring. The solution was stirred at RT for 1 h and evaporated to dryness to yield 6.6 g (99%) of the crude product. NMR/ppm δ_H_: 2.85 (s, CH_3_N), 4.70 (HOD); δ_B_: 1.0 (5%), 12.8 (10%), 19.1 (85%); δ_C_: 38.77 (*C*H_3_), 161.3 (*C*N_3_). FTIR (KBr cm^−1^): 3164(m), 1669(m), 1377(s), 1291(s), 1041(m), 917(s), 772(m), 725(m). A sample (0.3 g) of this product was recrystallized from H_2_O to afford crystals suitable for sc-XRD studies. C_5_H_18_B_5_N_3_O_13_, *M_r_* = 334.27, triclinic, *P*-1 (no.2), *a* = 9.5035(3) Å, *b* = 9.5151(3)Å, *c* = 10.4386(3) Å, *α* = 65.3810(10)°, *β* = 69.049(2), *γ* = 88.603(2), *V* = 792.72(4) Å^3^, *T* = 100(2) K, *Z* = 2; 16,517 reflections were measured, 3638 unique (*R_int_* = 0.0406), which were used in all calculations. The final *wR_2_* was 0.0935 (all data) and *R_1_* was 0.0373 (I > 2σ(I)).

### 3.7. Preparation of [C(NHMe)(NMe_2_)_2_]I

C(NH)(NMe_2_) (2.1 g, 2.3 mL, 18.2 mmol) was added to an Ar charged two-necked flask, and to this, CH_3_CN (10 mL) was added. The sealed vessel was then cooled to 0 °C using an ice bath. MeI (2.9 g, 20.4 mmol) was then added to CH_3_CN (8 mL) and this solution was added dropwise to the cold C(NH)(NMe_2_) solution. The mixture was then stirred for 12 h and left to slowly equilibrate to room temperature. The solvents were then removed via rotary evaporation. The resulting oil was then washed with EtOAc (3 × 25 mL) and dried under vacuum at room temperature to yield a white crystalline product [C(NHMe)(NMe_2_)_2_]I (3.2 g, 63%). NMR/ppm: δ_H_: 2.73 (3H, s, CH_3_N), 2.80 (12H, s, CH_3_N); δ_C_: 38.91 (*C*H_3_).

### 3.8. Preparation of [C(NHMe)(NMe_2_)_2_][B_5_O_6_(OH)_4_]·B(OH)_3_ (***5***)

[C(NHMe)(NMe_2_)_2_]I (1.0 g, 4.0 mmol) was dissolved in H_2_O (25 mL) along with a DOWEX 550A (OH)^−^ ion exchange resin (18 g) and stirred for 18 h. The resin was removed by filtration and the filtrate was added to B(OH)_3_ (1.3 g, 21 mmol). The solution was stirred for 2 h and evaporated under reduced pressure to yield 1.4 g of a white powder as a crude product (97%). A 0.3 g sample of the product was redissolved in H_2_O (15 mL) and white crystals suitable for X-ray diffraction studies were obtained after a few days. C_6_H_23_B_6_N_3_O_13_. Anal. Calc.: C = 17.6%, H = 5.7%, N = 10.2%. Found: C = 17.7%, H = 5.5%, N = 10.1%. NMR/ppm: δ_H_: 2.73 (3H, s, CH_3_N), 2.80 (12H, s, CH_3_N), 2.82 (1H, s), 4.70 (HOD); δ_C_: 38.89 (*C*H_3_). δ_B_: 1.1 (9%), 13.2 (13%), 18.8 (78%); FTIR (KBr, cm^−1^): 3370(s), 3236(s), 1625(s), 1592(m), 1426(s), 1398(m), 1367(s), 1324(m), 1156(m), 1022(m), 922(vs), 779(m), 709(m). XRD crystallographic data: C_6_H_23_B_6_N_3_O_13_, *M_r_* = 410.13, monoclinic, *P*2_1_/*c* (no. 14), *a* = 9.49570(10) Å, *b* = 11.44900(10) Å, *c* = 16.84590(10) Å, *β* = 98.0710(10), *α* = *γ* = 90, *V* = 1813.28(3) Å^3^, *T* = 100(2) K, *Z* = 4, *Z*′ = 1; 20,493 reflections were measured, 3281 unique (*R_int_* = 0.0196), which were used in all calculations. The final *wR_2_* was 0.0674 (all data) and *R_1_* was 0.0263 (I > 2σ(I)).

### 3.9. Preparation of [TBDH][B_5_O_6_(OH)_4_] (***6***)

1,5,7-Triazabicyclo[4.4.0]dec-5-ene (TBD, 1.0 g, 7.2 mmol) was dissolved in H_2_O (20 mL) and to this was added B(OH)_3_ (2.2 g, 36 mmol). The resulting solution was stirred under gentle heating to fully dissolve the B(OH)_3_ and left for 3 h. The solution was then evaporated under reduced pressure to yield a crude white product (2.4 g, 6.7 mmol, 93%).

A small sample of this solid (0.3 g) was redissolved in H_2_O (20 mL) and left to recrystallize over 7 d to afford a small number of white crystals, suitable for X-ray diffraction studies. C_7_H_18_B_5_N_3_O_10_. Anal. Calc.: C = 23.5%, H = 5.1%, N = 11.7%. Found: C = 23.7%, H = 5.0%, N = 11.5%. TGA: 245–295 °C, condensation of pentaborate with the loss of 2 H_2_O, 11.0% (10.0% calc.); 295–700 °C, oxidation of organic residue leaving residual 2.5 B_2_O_3_ 49.0% (48.5% calc.). NMR/ppm: δ_H_: 1.84 (4H, quin, CH_2_), 3.12 (4H, t, CH_2_N), 3.20 (4H, t, CH_2_N), 4.70 (HOD); δ_C_: 20.12 (*C*H_2_), 37.71 (*C*H_2_), 46.38 (*C*H_2_). δ_B_: 0.8 (1%), 13.0 (29%), 17.7 (70%). FTIR (KBr/cm^−1^): 3238(m), 1630(m),1408(m), 1294(s), 1144(m), 1086(m), 1015(m), 910(vs), 816(m), 772(s), 723(m), 706(s). XRD crystallographic data: C_7_H_18_B_5_N_3_O_10_, *M_r_* = 358.29, triclinic, *P*-1 (no. 2), *a* = 9.3096(6) Å, *b* = 9.3175(3) Å, *c* = 9.3733(6) Å, *α* = 76.598(5)°, *β* = 85.611(5)°, γ = 79.947(4)°, *V* = 778.22(8) Å^3^, *T* = 100(2) K, *Z* = 2, *Z*′ = 1; 13,382 reflections were measured, 2836 unique (*R_int_* = 0.0443), which were used in all calculations. The final *wR_2_* was 0.1747 (all data) and *R_1_* was 0.0561 (I > 2σ(I)).

## 4. Conclusions

The synthesis and structural characterization of six *N*-substituted guanidinium pentaborate(1-) salts was accomplished. Reactant stoichiometry, solid-state packing, and H-bonding interactions are all important factors in the self-assembly of these structures. An analysis of the H-bonding motifs in these salts and in related unsubstituted guanidinium polyborates was undertaken to evaluate this contribution. This analysis indicated that strong charge-assisted pincer R_2_^2^(8) motifs originating from the cation were in competition with the strong borate–borate R_2_^2^(8) motifs for the available borate acceptor sites. The ratios of potential cationic pincer-type H-bond donors to borate H-bond donors were determined for all compounds and compared with the ratios observed in their solid-state structures. Differences in these values were indicative of a structural preference for one or the other of these H-bonding motifs. The salts [C(NH_2_)_3_]_2_[B_4_O_5_(OH)_4_]·2H_2_O and [C(NH_2_)_3_]_3_[B_9_O_12_(OH)_6_] had potential ratios of >1 with the observed ratios higher than their potential ratios, indicating that the cation–anion pincer H-bond motifs were favoured. In contrast, [C(NH_2_)_3_][B_5_O_6_(OH)_4_]^.^H_2_O and the six new substituted guanidinium pentaborates described within this manuscript all had potential ratios of <1 and the observed ratios were generally lower than their potential ratios, indicating favourable borate–borate H-bond interactions. However, as both types of H-bond motifs were expected to be equally strong, other factors such as stoichiometry and crystal packing were strongly influential in determining the observed structures.

## Data Availability

Crystallographic available from CCDC, UK, see Supplementary Materials for details. CIF files for published unsubstituted guanidinium polyborate salts were also obtained from CCDC: 146622 (WEHFAQ), 1132636 (CUGNAT), and 1824332 (QUFJAF).

## Supplementary Materials

The following supporting information can be downloaded at: https://www.mdpi.com/article/10.3390/molecules28073273/s1, Crystallographic data for compounds **1**–**6** are available as Supplementary Materials. CCDC 2249242-7 also contain the supplementary crystallographic data for this paper. These CCDC data can be obtained free of charge via http://www.ccdc.cam.ac.uk/conts/retrieving.html (or from CCDC, 12 Union Road, Cambridge, CB2 1EZ. Fax: +44 1223 336033; E-mail: deposit@ccdc.cam.ac.uk).

## Sample Availability

Samples of the compounds are not available from the authors.
