# Supplementary material for: Structural (XRD) Characterization and an Analysis of H-Bonding Motifs in Some Tetrahydroxidohexaoxidopentaborate(1-) Salts of N-Substituted Guanidinium Cations
Source: Molecules. 2023 Apr 6;28(7):3273. doi: 10.3390/molecules28073273 (PMC10096507; doi:10.3390/molecules28073273)

checkCIF/PLATON report

Structure factors have been supplied for datablock(s) MAB3

THIS REPORT IS FOR GUIDANCE ONLY. IF USED AS PART OF A REVIEW PROCEDURE FOR PUBLICATION, IT SHOULD NOT REPLACE THE EXPERTISE OF AN EXPERIENCED CRYSTALLOGRAPHIC REFEREE.

No syntax errors found. CIF dictionary Interpreting this report

Datablock: MAB3

Bond precision:	N- C = 0.0012 A		Wavelength=0.71075
Cell:	a=9.9747(2)	b=11.2563(3)	c=11.6174(3)
	alpha=90	beta=96.084(2)	gamma=90
Temperature:	100 K		

	Calculated	Reported
Volume	1297.03(6)	1297.03(5)
Space group	P 21/c	P 1 21/c 1
Hall group	-P 2ybc	-P 2ybc
Moiety formula	B5 H4 O10, C3 H10 N3	B5 H4 O10, C3 H10 N3
Sum formula	C3 H14 B5 N3 O10	C3 H14 B5 N3 O10
Mr	306.22	306.22
Dx,g cm-3	1.568	1.568
Z	4	4
Mu (mm-1)	0.142	0.142
F000	632.0	632.0
F000’	632.43	
h,k,lmax	12,14,15	12,14,15
Nref	2971	2969
Tmin,Tmax	0.975,0.996	0.643,1.000
Tmin’	0.961	

Correction method= # Reported T Limits: Tmin=0.643 Tmax=1.000
AbsCorr = MULTI-SCAN

Data completeness= 0.999 Theta(max)= 27.483

R(reflections)= 0.0280(2722) wR2(reflections)= 0.0811(2969)

S = 1.035 Npar= 224

The following ALERTS were generated. Each ALERT has the format
test-name_ALERT_alert-type_alert-level.
Click on the hyperlinks for more details of the test.

Alert level C

```
PLAT250_ALERT_2_C Large U3/U1 Ratio for Average U(i,j) Tensor ....        2.3 Note
PLAT911_ALERT_3_C Missing FCF Refl Between Thmin & STh/L=    0.600          2 Report
```

Alert level G

```
PLAT480_ALERT_4_G Long H...A H-Bond Reported H2E      ..O1       .       2.61 Ang.
PLAT480_ALERT_4_G Long H...A H-Bond Reported H2B      ..O5       .       2.85 Ang.
PLAT480_ALERT_4_G Long H...A H-Bond Reported H9       ..O3       .       2.77 Ang.
PLAT480_ALERT_4_G Long H...A H-Bond Reported H8       ..O4       .       2.80 Ang.
PLAT480_ALERT_4_G Long H...A H-Bond Reported H10      ..O3       .       2.79 Ang.
PLAT480_ALERT_4_G Long H...A H-Bond Reported H7       ..O8       .       2.76 Ang.
PLAT910_ALERT_3_G Missing # of FCF Reflection(s) Below Theta(Min).          1 Note
PLAT913_ALERT_3_G Missing # of Very Strong Reflections in FCF ....          2 Note
```

0 **ALERT level A** = Most likely a serious problem - resolve or explain
0 **ALERT level B** = A potentially serious problem, consider carefully
2 **ALERT level C** = Check. Ensure it is not caused by an omission or oversight
8 **ALERT level G** = General information/check it is not something unexpected

0 ALERT type 1 CIF construction/syntax error, inconsistent or missing data
1 ALERT type 2 Indicator that the structure model may be wrong or deficient
3 ALERT type 3 Indicator that the structure quality may be low
6 ALERT type 4 Improvement, methodology, query or suggestion
0 ALERT type 5 Informative message, check

It is advisable to attempt to resolve as many as possible of the alerts in all categories. Often the minor alerts point to easily fixed oversights, errors and omissions in your CIF or refinement strategy, so attention to these fine details can be worthwhile. In order to resolve some of the more serious problems it may be necessary to carry out additional measurements or structure refinements. However, the purpose of your study may justify the reported deviations and the more serious of these should normally be commented upon in the discussion or experimental section of a paper or in the "special_details" fields of the CIF. checkCIF was carefully designed to identify outliers and unusual parameters, but every test has its limitations and alerts that are not important in a particular case may appear. Conversely, the absence of alerts does not guarantee there are no aspects of the results needing attention. It is up to the individual to critically assess their own results and, if necessary, seek expert advice.

Publication of your CIF in IUCr journals

A basic structural check has been run on your CIF. These basic checks will be run on all CIFs submitted for publication in IUCr journals (*Acta Crystallographica*, *Journal of Applied Crystallography*, *Journal of Synchrotron Radiation*); however, if you intend to submit to *Acta Crystallographica Section C* or *E* or *IUCrData*, you should make sure that full publication checks are run on the final version of your CIF prior to submission.

Publication of your CIF in other journals

Please refer to the *Notes for Authors* of the relevant journal for any special instructions relating to CIF submission.

PLATON version of 28/11/2022; check.def file version of 28/11/2022

Datablock MAB3 - ellipsoid plot

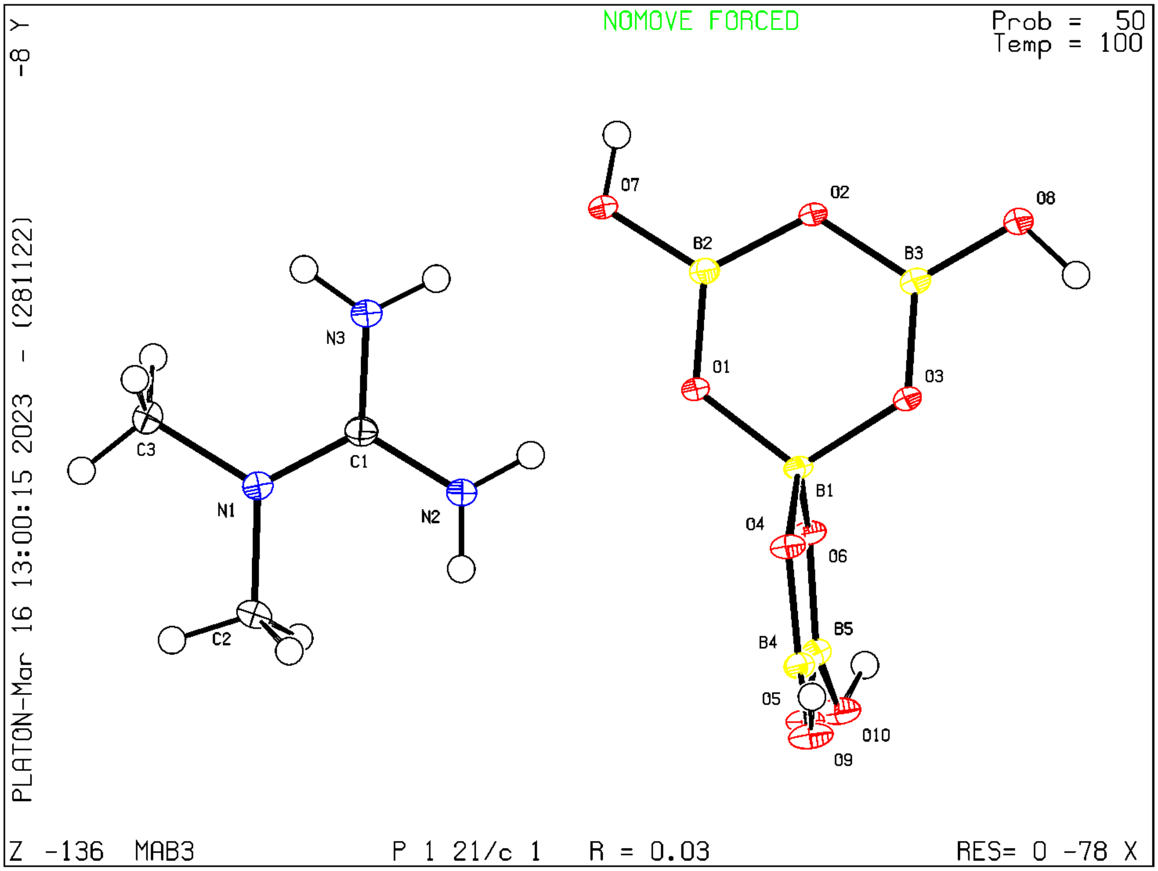

Supplement: Supplementary file 1 [file molecules-28-03273-s001.zip › MAB3_checkcif.pdf]
