# Supplementary material for: Structural (XRD) Characterization and an Analysis of H-Bonding Motifs in Some Tetrahydroxidohexaoxidopentaborate(1-) Salts of N-Substituted Guanidinium Cations
Source: Molecules. 2023 Apr 6;28(7):3273. doi: 10.3390/molecules28073273 (PMC10096507; doi:10.3390/molecules28073273)

checkCIF/PLATON report

Structure factors have been supplied for datablock(s) MAB5

THIS REPORT IS FOR GUIDANCE ONLY. IF USED AS PART OF A REVIEW PROCEDURE FOR PUBLICATION, IT SHOULD NOT REPLACE THE EXPERTISE OF AN EXPERIENCED CRYSTALLOGRAPHIC REFEREE.

No syntax errors found. CIF dictionary Interpreting this report

Datablock: MAB5

Bond precision: N- C = 0.0015 A Wavelength=1.54178

Cell:	a=9.4957(1)	b=11.4490(1)	c=16.8459(1)
	alpha=90	beta=98.071(1)	gamma=90
Temperature:	100 K		

	Calculated	Reported
Volume	1813.28(3)	1813.28(3)
Space group	P 21/c	P 1 21/c 1
Hall group	-P 2ybc	-P 2ybc
Moiety formula	B5 H4 O10, C6 H16 N3, B H3 O3	C6 H16 N3, B5 H4 O10, B H3 O3
Sum formula	C6 H23 B6 N3 O13	C6 H23 B6 N3 O13
Mr	410.13	410.13
Dx,g cm-3	1.502	1.502
Z	4	4
Mu (mm-1)	1.164	1.164
F000	856.0	856.0
F000'	859.43	
h,k,lmax	11,13,20	11,13,20
Nref	3326	3281
Tmin,Tmax	0.778,0.911	0.804,1.000
Tmin'	0.774	

Correction method= # Reported T Limits: Tmin=0.804 Tmax=1.000
AbsCorr = MULTI-SCAN

Data completeness= 0.986 Theta(max)= 68.239

R(reflections)= 0.0263(3208) wR2(reflections)= 0.0674(3281)

S = 1.004 Npar= 290

The following ALERTS were generated. Each ALERT has the format
test-name_ALERT_alert-type_alert-level.
Click on the hyperlinks for more details of the test.

Alert level C

```
PLAT042_ALERT_1_C Calc. and Reported MoietyFormula Strings  Differ     Please Check
PLAT911_ALERT_3_C Missing FCF Refl Between Thmin & STh/L=    0.600         43 Report
PLAT913_ALERT_3_C Missing # of Very Strong Reflections in FCF ....         13 Note
```

Alert level G

```
PLAT142_ALERT_4_G s.u. on b - Axis Small or Missing .............    0.00010 Ang.
PLAT143_ALERT_4_G s.u. on c - Axis Small or Missing .............    0.00010 Ang.
PLAT153_ALERT_1_G The s.u.'s on the Cell Axes   are Equal ..(Note)     0.0001 Ang.
PLAT432_ALERT_2_G Short Inter X...Y Contact  O12      ..C1       .       2.95 Ang.
                                                     x,y,z  =      1_555 Check
PLAT912_ALERT_4_G Missing # of FCF Reflections Above STh/L=  0.600          2 Note
```

0 **ALERT level A** = Most likely a serious problem - resolve or explain
0 **ALERT level B** = A potentially serious problem, consider carefully
3 **ALERT level C** = Check. Ensure it is not caused by an omission or oversight
5 **ALERT level G** = General information/check it is not something unexpected

2 ALERT type 1 CIF construction/syntax error, inconsistent or missing data
1 ALERT type 2 Indicator that the structure model may be wrong or deficient
2 ALERT type 3 Indicator that the structure quality may be low
3 ALERT type 4 Improvement, methodology, query or suggestion
0 ALERT type 5 Informative message, check

It is advisable to attempt to resolve as many as possible of the alerts in all categories. Often the minor alerts point to easily fixed oversights, errors and omissions in your CIF or refinement strategy, so attention to these fine details can be worthwhile. In order to resolve some of the more serious problems it may be necessary to carry out additional measurements or structure refinements. However, the purpose of your study may justify the reported deviations and the more serious of these should normally be commented upon in the discussion or experimental section of a paper or in the "special_details" fields of the CIF. checkCIF was carefully designed to identify outliers and unusual parameters, but every test has its limitations and alerts that are not important in a particular case may appear. Conversely, the absence of alerts does not guarantee there are no aspects of the results needing attention. It is up to the individual to critically assess their own results and, if necessary, seek expert advice.

Publication of your CIF in IUCr journals

A basic structural check has been run on your CIF. These basic checks will be run on all CIFs submitted for publication in IUCr journals (*Acta Crystallographica*, *Journal of Applied Crystallography*, *Journal of Synchrotron Radiation*); however, if you intend to submit to *Acta Crystallographica Section C* or *E* or *IUCrData*, you should make sure that full publication checks are run on the final version of your CIF prior to submission.

Publication of your CIF in other journals

Please refer to the *Notes for Authors* of the relevant journal for any special instructions relating to CIF submission.

PLATON version of 28/11/2022; check.def file version of 28/11/2022

Datablock MAB5 - ellipsoid plot

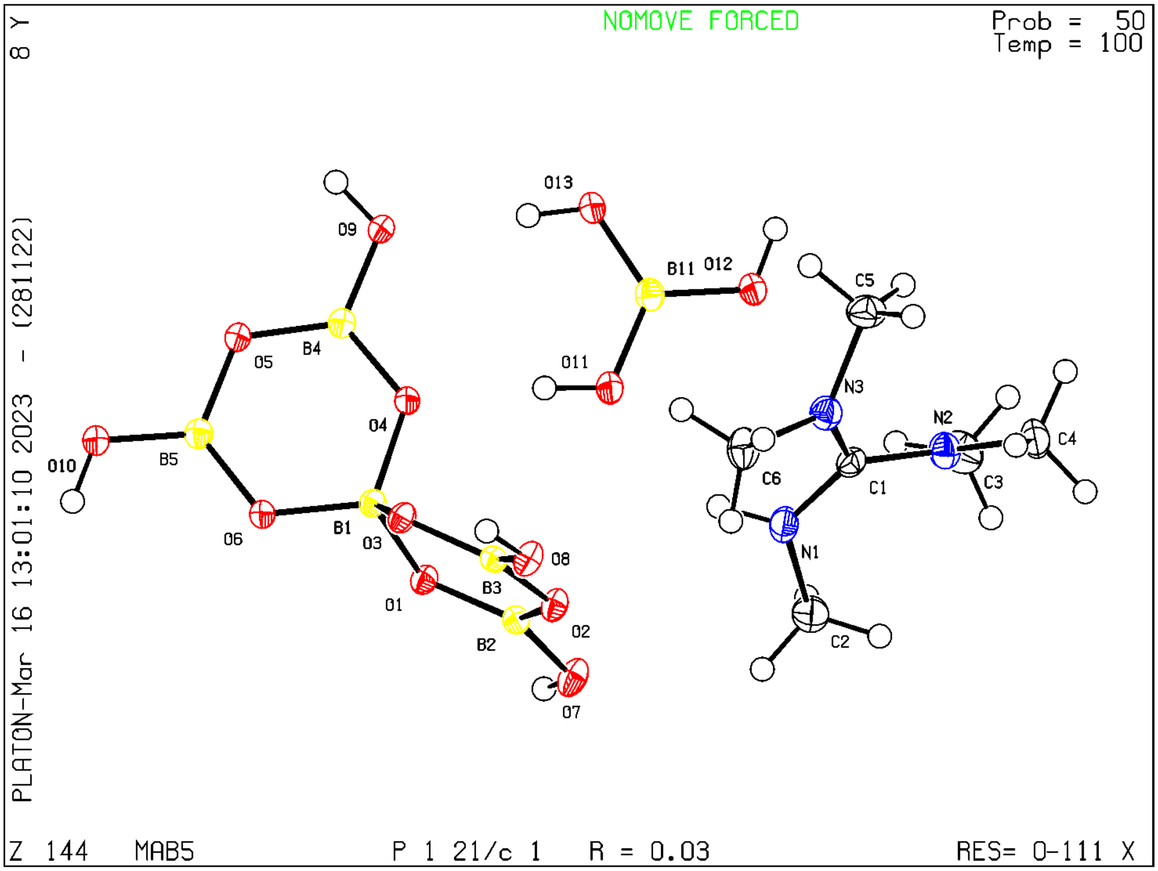

Supplement: Supplementary file 1 [file molecules-28-03273-s001.zip › MAB5_checkcif.pdf]
