# Supplementary material for: Structural (XRD) Characterization and an Analysis of H-Bonding Motifs in Some Tetrahydroxidohexaoxidopentaborate(1-) Salts of N-Substituted Guanidinium Cations
Source: Molecules. 2023 Apr 6;28(7):3273. doi: 10.3390/molecules28073273 (PMC10096507; doi:10.3390/molecules28073273)

checkCIF/PLATON report

Structure factors have been supplied for datablock(s) MAB6

THIS REPORT IS FOR GUIDANCE ONLY. IF USED AS PART OF A REVIEW PROCEDURE FOR PUBLICATION, IT SHOULD NOT REPLACE THE EXPERTISE OF AN EXPERIENCED CRYSTALLOGRAPHIC REFEREE.

No syntax errors found. CIF dictionary Interpreting this report

Datablock: MAB6

Bond precision:	= 0.0000 A		Wavelength=1.54178
Cell:	a=9.3096(6)	b=9.3175(3)	c=9.3733(6)
	alpha=76.598(5)	beta=85.611(5)	gamma=79.947(4)
Temperature:	100 K		

	Calculated	Reported
Volume	778.22(8)	778.22(8)
Space group	P -1	P -1
Hall group	-P 1	-P 1
Moiety formula	0.5(B10 H8 O20), B5 H4 O10, 2(C7 H14 N3)	B5 H4 O10, C7 H14 N3
Sum formula	C14 H36 B10 N6 O20	C7 H18 B5 N3 O10
Mr	716.59	358.29
Dx,g cm-3	1.529	1.529
Z	1	2
Mu (mm-1)	1.133	1.133
F000	372.0	372.0
F000'	373.44	
h,k,lmax	11,11,11	11,11,11
Nref	2858	2836
Tmin,Tmax	0.861,0.978	0.803,1.000
Tmin'	0.788	

Correction method= # Reported T Limits: Tmin=0.803 Tmax=1.000
AbsCorr = MULTI-SCAN

Data completeness= 0.992 Theta(max)= 68.221

R(reflections)= 0.0561(2429) wR2(reflections)= 0.1747(2836)

S = 1.076 Npar= 459

The following ALERTS were generated. Each ALERT has the format
test-name_ALERT_alert-type_alert-level.
Click on the hyperlinks for more details of the test.

Alert level B

```
PLAT088_ALERT_3_B Poor Data / Parameter Ratio ....................       6.18 Note
```

Alert level C

```
PLAT042_ALERT_1_C Calc. and Reported MoietyFormula Strings  Differ     Please Check
PLAT906_ALERT_3_C Large K Value in the Analysis of Variance ......      2.001 Check
PLAT911_ALERT_3_C Missing FCF Refl Between Thmin & STh/L=    0.600        19 Report
PLAT977_ALERT_2_C Check Negative Difference Density on H2A       .      -0.31 eA-3
```

Alert level G

```
PLAT002_ALERT_2_G Number of Distance or Angle Restraints on AtSite        20 Note
PLAT003_ALERT_2_G Number of Uiso or Uij Restrained non-H Atoms ...        50 Report
PLAT007_ALERT_5_G Number of Unrefined Donor-H Atoms .............        12 Report
PLAT045_ALERT_1_G Calculated and Reported Z Differ by a Factor ...     0.500 Check
PLAT072_ALERT_2_G SHELXL First  Parameter in WGHT  Unusually Large      0.12 Report
PLAT172_ALERT_4_G The CIF-Embedded .res File Contains DFIX Records         1 Report
PLAT174_ALERT_4_G The CIF-Embedded .res File Contains FLAT Records         2 Report
PLAT176_ALERT_4_G The CIF-Embedded .res File Contains SADI Records        27 Report
PLAT178_ALERT_4_G The CIF-Embedded .res File Contains SIMU Records         1 Report
PLAT187_ALERT_4_G The CIF-Embedded .res File Contains RIGU Records         1 Report
PLAT191_ALERT_3_G A Non-default SADI Restraint Value has been used    0.0300 Report
PLAT191_ALERT_3_G A Non-default SADI Restraint Value has been used    0.0300 Report
PLAT191_ALERT_3_G A Non-default SADI Restraint Value has been used    0.0300 Report
PLAT191_ALERT_3_G A Non-default SADI Restraint Value has been used    0.0300 Report
PLAT191_ALERT_3_G A Non-default SADI Restraint Value has been used    0.0300 Report
PLAT191_ALERT_3_G A Non-default SADI Restraint Value has been used    0.0300 Report
PLAT191_ALERT_3_G A Non-default SADI Restraint Value has been used    0.0300 Report
PLAT191_ALERT_3_G A Non-default SADI Restraint Value has been used    0.0300 Report
PLAT191_ALERT_3_G A Non-default SADI Restraint Value has been used    0.0300 Report
PLAT191_ALERT_3_G A Non-default SADI Restraint Value has been used    0.0600 Report
PLAT191_ALERT_3_G A Non-default SADI Restraint Value has been used    0.0600 Report
PLAT191_ALERT_3_G A Non-default SADI Restraint Value has been used    0.0600 Report
PLAT191_ALERT_3_G A Non-default SADI Restraint Value has been used    0.0600 Report
PLAT191_ALERT_3_G A Non-default SADI Restraint Value has been used    0.0600 Report
PLAT191_ALERT_3_G A Non-default SADI Restraint Value has been used    0.0600 Report
PLAT191_ALERT_3_G A Non-default SADI Restraint Value has been used    0.0600 Report
PLAT191_ALERT_3_G A Non-default SADI Restraint Value has been used    0.0600 Report
PLAT191_ALERT_3_G A Non-default SADI Restraint Value has been used    0.0600 Report
PLAT191_ALERT_3_G A Non-default SADI Restraint Value has been used    0.0600 Report
PLAT191_ALERT_3_G A Non-default SADI Restraint Value has been used    0.0600 Report
PLAT191_ALERT_3_G A Non-default SADI Restraint Value has been used    0.0600 Report
PLAT191_ALERT_3_G A Non-default SADI Restraint Value has been used    0.0300 Report
PLAT191_ALERT_3_G A Non-default SADI Restraint Value has been used    0.0300 Report
PLAT191_ALERT_3_G A Non-default SADI Restraint Value has been used    0.0600 Report
PLAT191_ALERT_3_G A Non-default SADI Restraint Value has been used    0.0600 Report
PLAT191_ALERT_3_G A Non-default SADI Restraint Value has been used    0.0300 Report
PLAT300_ALERT_4_G Atom Site Occupancy of O1         Constrained at       0.5 Check
PLAT300_ALERT_4_G Atom Site Occupancy of O2         Constrained at       0.5 Check
```

```
PLAT300_ALERT_4_G Atom Site Occupancy of O3         Constrained at        0.5 Check
PLAT300_ALERT_4_G Atom Site Occupancy of O4         Constrained at        0.5 Check
PLAT300_ALERT_4_G Atom Site Occupancy of O5         Constrained at        0.5 Check
PLAT300_ALERT_4_G Atom Site Occupancy of O6         Constrained at        0.5 Check
PLAT300_ALERT_4_G Atom Site Occupancy of O7         Constrained at        0.5 Check
PLAT300_ALERT_4_G Atom Site Occupancy of O8         Constrained at        0.5 Check
PLAT300_ALERT_4_G Atom Site Occupancy of O9         Constrained at        0.5 Check
PLAT300_ALERT_4_G Atom Site Occupancy of O10        Constrained at        0.5 Check
PLAT300_ALERT_4_G Atom Site Occupancy of B1         Constrained at        0.5 Check
PLAT300_ALERT_4_G Atom Site Occupancy of B2         Constrained at        0.5 Check
PLAT300_ALERT_4_G Atom Site Occupancy of B3         Constrained at        0.5 Check
PLAT300_ALERT_4_G Atom Site Occupancy of B4         Constrained at        0.5 Check
PLAT300_ALERT_4_G Atom Site Occupancy of B5         Constrained at        0.5 Check
PLAT300_ALERT_4_G Atom Site Occupancy of H7         Constrained at        0.5 Check
PLAT300_ALERT_4_G Atom Site Occupancy of H8         Constrained at        0.5 Check
PLAT300_ALERT_4_G Atom Site Occupancy of H9         Constrained at        0.5 Check
PLAT300_ALERT_4_G Atom Site Occupancy of H10        Constrained at        0.5 Check
PLAT300_ALERT_4_G Atom Site Occupancy of O11        Constrained at        0.5 Check
PLAT300_ALERT_4_G Atom Site Occupancy of O12        Constrained at        0.5 Check
PLAT300_ALERT_4_G Atom Site Occupancy of O13        Constrained at        0.5 Check
PLAT300_ALERT_4_G Atom Site Occupancy of O14        Constrained at        0.5 Check
PLAT300_ALERT_4_G Atom Site Occupancy of O15        Constrained at        0.5 Check
PLAT300_ALERT_4_G Atom Site Occupancy of O16        Constrained at        0.5 Check
PLAT300_ALERT_4_G Atom Site Occupancy of O17        Constrained at        0.5 Check
PLAT300_ALERT_4_G Atom Site Occupancy of O18        Constrained at        0.5 Check
PLAT300_ALERT_4_G Atom Site Occupancy of O19        Constrained at        0.5 Check
PLAT300_ALERT_4_G Atom Site Occupancy of O20        Constrained at        0.5 Check
PLAT300_ALERT_4_G Atom Site Occupancy of B11        Constrained at        0.5 Check
PLAT300_ALERT_4_G Atom Site Occupancy of B12        Constrained at        0.5 Check
PLAT300_ALERT_4_G Atom Site Occupancy of B13        Constrained at        0.5 Check
PLAT300_ALERT_4_G Atom Site Occupancy of B14        Constrained at        0.5 Check
PLAT300_ALERT_4_G Atom Site Occupancy of B15        Constrained at        0.5 Check
PLAT300_ALERT_4_G Atom Site Occupancy of H17        Constrained at        0.5 Check
PLAT300_ALERT_4_G Atom Site Occupancy of H18        Constrained at        0.5 Check
PLAT300_ALERT_4_G Atom Site Occupancy of H19        Constrained at        0.5 Check
PLAT300_ALERT_4_G Atom Site Occupancy of H20        Constrained at        0.5 Check
PLAT300_ALERT_4_G Atom Site Occupancy of N1         Constrained at        0.5 Check
PLAT300_ALERT_4_G Atom Site Occupancy of N2         Constrained at        0.5 Check
PLAT300_ALERT_4_G Atom Site Occupancy of N3         Constrained at        0.5 Check
PLAT300_ALERT_4_G Atom Site Occupancy of C1         Constrained at        0.5 Check
PLAT300_ALERT_4_G Atom Site Occupancy of C2         Constrained at        0.5 Check
PLAT300_ALERT_4_G Atom Site Occupancy of C3         Constrained at        0.5 Check
PLAT300_ALERT_4_G Atom Site Occupancy of C4         Constrained at        0.5 Check
PLAT300_ALERT_4_G Atom Site Occupancy of C5         Constrained at        0.5 Check
PLAT300_ALERT_4_G Atom Site Occupancy of C6         Constrained at        0.5 Check
PLAT300_ALERT_4_G Atom Site Occupancy of C7         Constrained at        0.5 Check
PLAT300_ALERT_4_G Atom Site Occupancy of H2         Constrained at        0.5 Check
PLAT300_ALERT_4_G Atom Site Occupancy of H2A        Constrained at        0.5 Check
PLAT300_ALERT_4_G Atom Site Occupancy of H2B        Constrained at        0.5 Check
PLAT300_ALERT_4_G Atom Site Occupancy of H3         Constrained at        0.5 Check
PLAT300_ALERT_4_G Atom Site Occupancy of H3A        Constrained at        0.5 Check
PLAT300_ALERT_4_G Atom Site Occupancy of H3B        Constrained at        0.5 Check
PLAT300_ALERT_4_G Atom Site Occupancy of H4A        Constrained at        0.5 Check
PLAT300_ALERT_4_G Atom Site Occupancy of H4B        Constrained at        0.5 Check
PLAT300_ALERT_4_G Atom Site Occupancy of H5A        Constrained at        0.5 Check
PLAT300_ALERT_4_G Atom Site Occupancy of H5B        Constrained at        0.5 Check
PLAT300_ALERT_4_G Atom Site Occupancy of H6A        Constrained at        0.5 Check
```

```
PLAT300_ALERT_4_G Atom Site Occupancy of H6B        Constrained at        0.5 Check
PLAT300_ALERT_4_G Atom Site Occupancy of H7A        Constrained at        0.5 Check
PLAT300_ALERT_4_G Atom Site Occupancy of H7B        Constrained at        0.5 Check
PLAT300_ALERT_4_G Atom Site Occupancy of N11        Constrained at        0.5 Check
PLAT300_ALERT_4_G Atom Site Occupancy of N12        Constrained at        0.5 Check
PLAT300_ALERT_4_G Atom Site Occupancy of N13        Constrained at        0.5 Check
PLAT300_ALERT_4_G Atom Site Occupancy of C11        Constrained at        0.5 Check
PLAT300_ALERT_4_G Atom Site Occupancy of C12        Constrained at        0.5 Check
PLAT300_ALERT_4_G Atom Site Occupancy of C13        Constrained at        0.5 Check
PLAT300_ALERT_4_G Atom Site Occupancy of C14        Constrained at        0.5 Check
PLAT300_ALERT_4_G Atom Site Occupancy of C15        Constrained at        0.5 Check
PLAT300_ALERT_4_G Atom Site Occupancy of C16        Constrained at        0.5 Check
PLAT300_ALERT_4_G Atom Site Occupancy of C17        Constrained at        0.5 Check
PLAT300_ALERT_4_G Atom Site Occupancy of H12        Constrained at        0.5 Check
PLAT300_ALERT_4_G Atom Site Occupancy of H12A       Constrained at        0.5 Check
PLAT300_ALERT_4_G Atom Site Occupancy of H12B       Constrained at        0.5 Check
PLAT300_ALERT_4_G Atom Site Occupancy of H13        Constrained at        0.5 Check
PLAT300_ALERT_4_G Atom Site Occupancy of H13A       Constrained at        0.5 Check
PLAT300_ALERT_4_G Atom Site Occupancy of H13B       Constrained at        0.5 Check
PLAT300_ALERT_4_G Atom Site Occupancy of H14A       Constrained at        0.5 Check
PLAT300_ALERT_4_G Atom Site Occupancy of H14B       Constrained at        0.5 Check
PLAT300_ALERT_4_G Atom Site Occupancy of H15A       Constrained at        0.5 Check
PLAT300_ALERT_4_G Atom Site Occupancy of H15B       Constrained at        0.5 Check
PLAT300_ALERT_4_G Atom Site Occupancy of H16A       Constrained at        0.5 Check
PLAT300_ALERT_4_G Atom Site Occupancy of H16B       Constrained at        0.5 Check
PLAT300_ALERT_4_G Atom Site Occupancy of H17A       Constrained at        0.5 Check
PLAT300_ALERT_4_G Atom Site Occupancy of H17B       Constrained at        0.5 Check
PLAT301_ALERT_3_G Main Residue  Disorder .............(Resd  1  )       100% Note
PLAT302_ALERT_4_G Anion/Solvent/Minor-Residue Disorder (Resd  2  )       100% Note
PLAT302_ALERT_4_G Anion/Solvent/Minor-Residue Disorder (Resd  3  )       100% Note
PLAT302_ALERT_4_G Anion/Solvent/Minor-Residue Disorder (Resd  4  )       100% Note
PLAT304_ALERT_4_G Non-Integer Number of Atoms in ..... (Resd  2  )       9.50 Check
PLAT802_ALERT_4_G CIF Input Record(s) with more than 80 Characters          1 Info
PLAT860_ALERT_3_G Number of Least-Squares Restraints ............        1234 Note
PLAT883_ALERT_1_G No Info/Value for _atom_sites_solution_primary .     Please Do !
PLAT912_ALERT_4_G Missing # of FCF Reflections Above STh/L=  0.600          3 Note
PLAT913_ALERT_3_G Missing # of Very Strong Reflections in FCF ....          3 Note
PLAT941_ALERT_3_G Average HKL Measurement Multiplicity ..........         4.7 Low
```

---

   0 **ALERT level A** = Most likely a serious problem - resolve or explain
   1 **ALERT level B** = A potentially serious problem, consider carefully
   4 **ALERT level C** = Check. Ensure it is not caused by an omission or oversight
 133 **ALERT level G** = General information/check it is not something unexpected

   3 ALERT type 1 CIF construction/syntax error, inconsistent or missing data
   4 ALERT type 2 Indicator that the structure model may be wrong or deficient
  33 ALERT type 3 Indicator that the structure quality may be low
  97 ALERT type 4 Improvement, methodology, query or suggestion
   1 ALERT type 5 Informative message, check

It is advisable to attempt to resolve as many as possible of the alerts in all categories. Often the minor alerts point to easily fixed oversights, errors and omissions in your CIF or refinement strategy, so attention to these fine details can be worthwhile. In order to resolve some of the more serious problems it may be necessary to carry out additional measurements or structure refinements. However, the purpose of your study may justify the reported deviations and the more serious of these should normally be commented upon in the discussion or experimental section of a paper or in the "special_details" fields of the CIF. checkCIF was carefully designed to identify outliers and unusual parameters, but every test has its limitations and alerts that are not important in a particular case may appear. Conversely, the absence of alerts does not guarantee there are no aspects of the results needing attention. It is up to the individual to critically assess their own results and, if necessary, seek expert advice.

Publication of your CIF in IUCr journals

A basic structural check has been run on your CIF. These basic checks will be run on all CIFs submitted for publication in IUCr journals (*Acta Crystallographica*, *Journal of Applied Crystallography*, *Journal of Synchrotron Radiation*); however, if you intend to submit to *Acta Crystallographica Section C* or *E* or *IUCrData*, you should make sure that full publication checks are run on the final version of your CIF prior to submission.

Publication of your CIF in other journals

Please refer to the *Notes for Authors* of the relevant journal for any special instructions relating to CIF submission.

PLATON version of 28/11/2022; check.def file version of 28/11/2022

Datablock MAB6 - ellipsoid plot

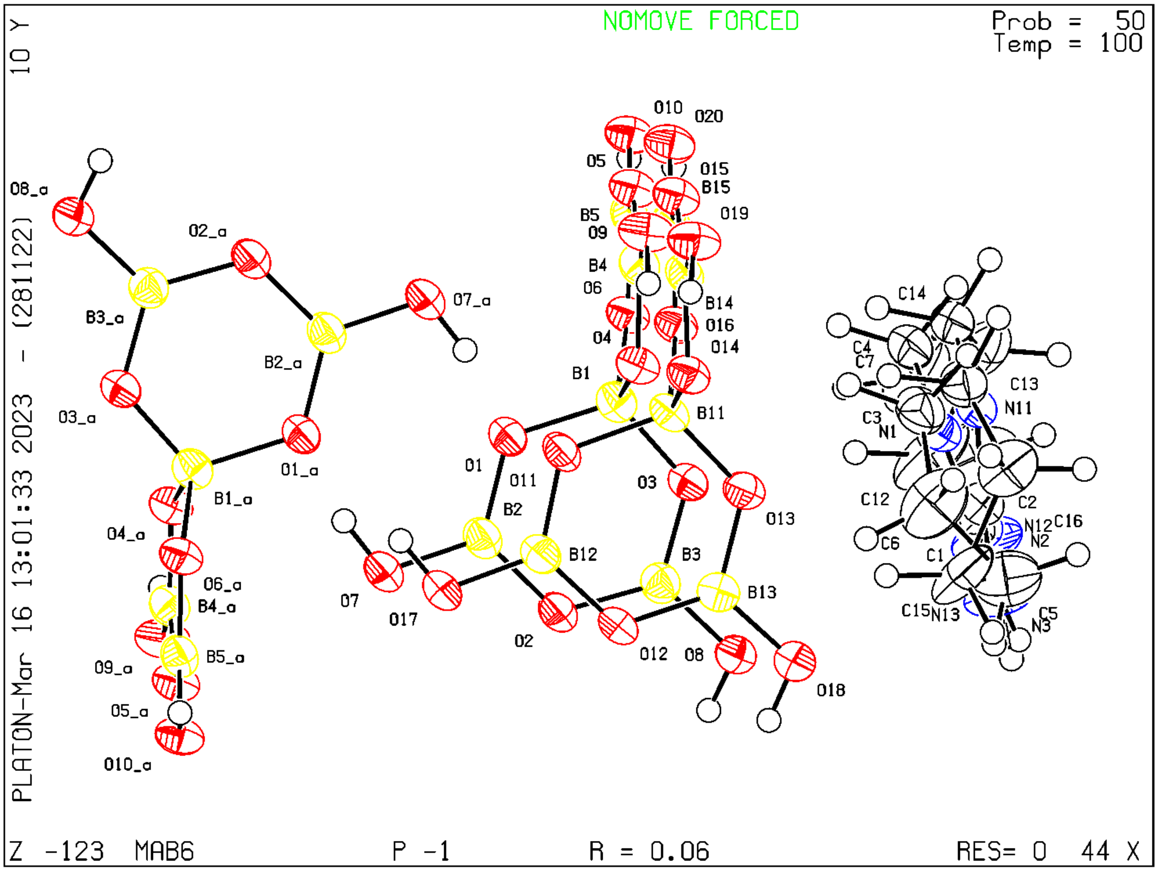

Supplement: Supplementary file 1 [file molecules-28-03273-s001.zip › MAB6_checkcif.pdf]
