# Supplementary material for: Structural (XRD) Characterization and an Analysis of H-Bonding Motifs in Some Tetrahydroxidohexaoxidopentaborate(1-) Salts of N-Substituted Guanidinium Cations
Source: Molecules. 2023 Apr 6;28(7):3273. doi: 10.3390/molecules28073273 (PMC10096507; doi:10.3390/molecules28073273)
Supplement: Supplementary file 1 [file molecules-28-03273-s001.zip › Supplementary X-ray Data Revised proofed.pdf]

Electronic Supplementary Data

X-ray data for 1-6

Structural (XRD) characterization and an analysis of H-bonding motifs in some tetrahydroxidohexaoxidopentaborate(1-) salts of *N*-substituted guanidinium cations

Michael A. Beckett [1*], Simon J. Coles[2], Peter N. Horton[2], and Thomas A. Rixon[1]

1 School of Natural Sciences, Bangor University, Bangor, Gwynedd, LL57 2UW, UK;

2 Chemistry Department, University of Southampton, Southampton, SO17 1BJ, UK;

* Correspondence: m.a.beckett@bangor.ac.uk

$[C(NH_2)_2(NHMe)][B_5O_6(OH)_4]\cdot H_2O$ (**1**)

Submitted by: **Mike A Beckett / Tom Rixon**
Bangor University
Solved by: **Peter N Horton**
Sample ID: **MAB/TR/Me|GaunB5**

R_1=2.67%

Crystal Data and Experimental

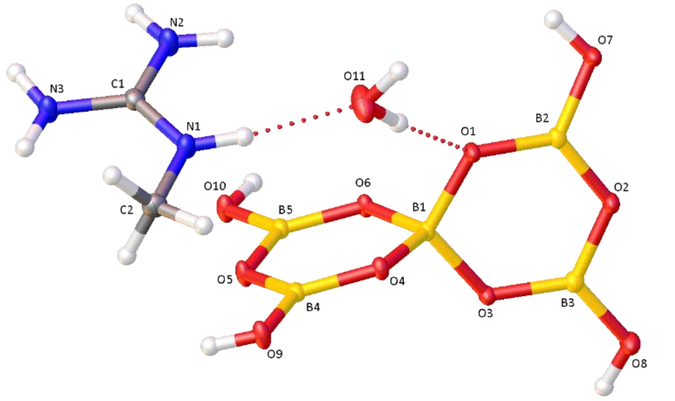

Experimental. Single colourless block crystals of **2019NCSX0350** were recrystallised from water. A suitable crystal with dimensions 0.400 × 0.400 × 0.200 mm^3 was selected and mounted on a MITIGEN holder in perfluoroether oil on a Rigaku FRE+ equipped with VHF Varimax confocal mirrors and an AFC12 goniometer and HyPix 6000 detector diffractometer. The crystal was kept at a steady T = 100(2) K during data collection. The structure was solved with the **ShelXT** 2018/2 (Sheldrick, 2018) solution program using dual methods and by using **Olex2** (Dolomanov et al., 2009) as the graphical interface. The model was refined with **ShelXL** 2018/3 (Sheldrick, 2015) using full matrix least squares minimisation on $\boldsymbol{F^2}$.

Crystal Data. $C_2H_{14}B_5N_3O_{11}$, M_r = 310.21, monoclinic, $P2_1/c$ (No. 14), a = 9.9962(2) Å, b = 10.9047(2) Å, c = 11.7215(2) Å, β = 96.101(2)°, α = γ = 90°, V = 1270.47(4) Å^3, T = 100(2) K, Z = 4, Z' = 1, μ(Mo K_α) = 0.151 mm^{-1}, 17592 reflections measured, 2893 unique (R_{int} = 0.0115) which were used in all calculations. The final wR_2 was 0.0743 (all data) and R_1 was 0.0267 (I > 2(I)).

**Compound**	**2019NCSX0350**
Formula	$C_2H_{14}B_5N_3O_{11}$
$D_{calc.}$/ g cm^{-3}	1.622
μ/mm^{-1}	0.151
Formula Weight	310.21
Colour	colourless
Shape	block
Size/mm^3	0.400×0.400×0.200
T/K	100(2)
Crystal System	monoclinic
Space Group	$P2_1/c$
a/Å	9.9962(2)
b/Å	10.9047(2)
c/Å	11.7215(2)
α/°	90
β/°	96.101(2)
γ/°	90
V/Å^3	1270.47(4)
Z	4
Z'	1
Wavelength/Å	0.71075
Radiation type	Mo K_α
Θ_{min}/°	2.558
Θ_{max}/°	27.485
Measured Refl's.	17592
Ind't Refl's	2893
Refl's with I > 2(I)	2810
R_{int}	0.0115
Parameters	235
Restraints	0
Largest Peak	0.246
Deepest Hole	-0.233
GooF	1.071
wR_2 (all data)	0.0743
wR_2	0.0737
R_1 (all data)	0.0275
R_1	0.0267

Structure Quality Indicators

**Reflections:**	d min (Mo) 0.77	I/σ 141.6	Rint 1.15%	complete 99% (IUCr) 99%
**Refinement:**	Shift 0.000	Max Peak 0.2	Min Peak -0.2	GooF 1.071

A colourless block-shaped crystal with dimensions 0.400 × 0.400 × 0.200 mm^3 was mounted on a MITIGEN holder in perfluoroether oil. Data were collected using a Rigaku FRE+ equipped with VHF Varimax confocal mirrors and an AFC12 goniometer and HyPix 6000 detector diffractometer equipped with an Oxford Cryosystems low-temperature device operating at T = 100(2) K.

Data were measured using profile data from ω-scans 0.5 ° per frame for 0.5 s using MoK_α radiation (Rotating Anode, 45.0 kV, 55.0 mA). The diffraction pattern was indexed and the total number of runs and images was based on the strategy calculation from the program **CrysAlisPro** (Rigaku, V1.171.40.53, 2019). . The maximum resolution that was achieved was Θ = 27.485° (0.77 Å).

The diffraction pattern was indexed and the total number of runs and images was based on the strategy calculation from the program **CrysAlisPro** (Rigaku, V1.171.40.53, 2019). The unit cell was refined using **CrysAlisPro** (Rigaku, V1.171.40.53, 2019) on 14324 reflections, 81% of the observed reflections.

Data reduction, scaling and absorption corrections were performed using **CrysAlisPro** (Rigaku, V1.171.40.53, 2019). The final completeness is 99.20 % out to 27.485° in Θ. A multi-scan absorption correction was performed using CrysAlisPro 1.171.40.53 (Rigaku Oxford Diffraction, 2019) using spherical harmonics as implemented in SCALE3 ABSPACK. The absorption coefficient μ of this material is 0.151 mm^{-1} at this wavelength (λ = 0.71075Å) and the minimum and maximum transmissions are 0.947 and 1.000.

The structure was solved and the space group $P2_1/c$ (# 14) determined by the ShelXT 2018/2 (Sheldrick, 2018) structure solution program using using dual methods and refined by full matrix least squares minimisation on $\boldsymbol{F}^2$ using version 2018/3 of ShelXL 2018/3 (Sheldrick, 2015). All non-hydrogen atoms were refined anisotropically. Most hydrogen atom positions were calculated geometrically and refined using the riding model, but some hydrogen atoms were refined freely.

_exptl_absorpt_process_details: CrysAlisPro 1.171.40.53 (Rigaku Oxford Diffraction, 2019) using spherical harmonics as implemented in SCALE3 ABSPACK.

There is a single molecule in the asymmetric unit, which is represented by the reported sum formula. In other words: Z is 4 and Z' is 1.

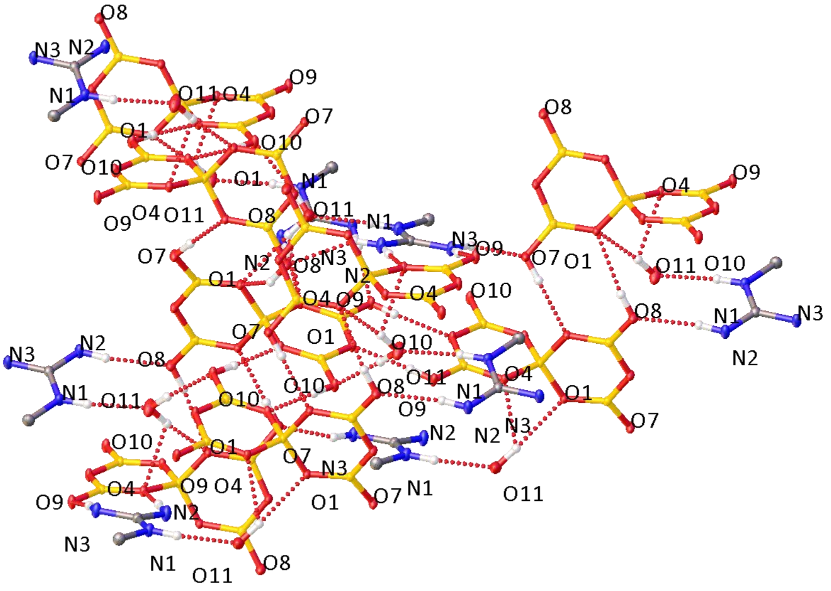

Figure S1: The following hydrogen bonding interactions with a maximum D-D distance of 3.1 Å and a minimum angle of 120 ° are present in **2019NCSX0350**: O10–O6_*1: 2.702 Å, O9–O5*_2: 2.818 Å, O7–O3_*3: 2.767 Å, O8–O1*_5: 3.046 Å, O11–O4: 3.079 Å, O11–O1: 3.043 Å, O11–O10_*4: 2.856 Å, N1–O11: 2.83 Å, N3–O9*_6: 3.013 Å, N3–O7_*7: 2.995 Å, N2–O4*_6: 2.962 Å, N2–O8_*3: 3.038 Å.*

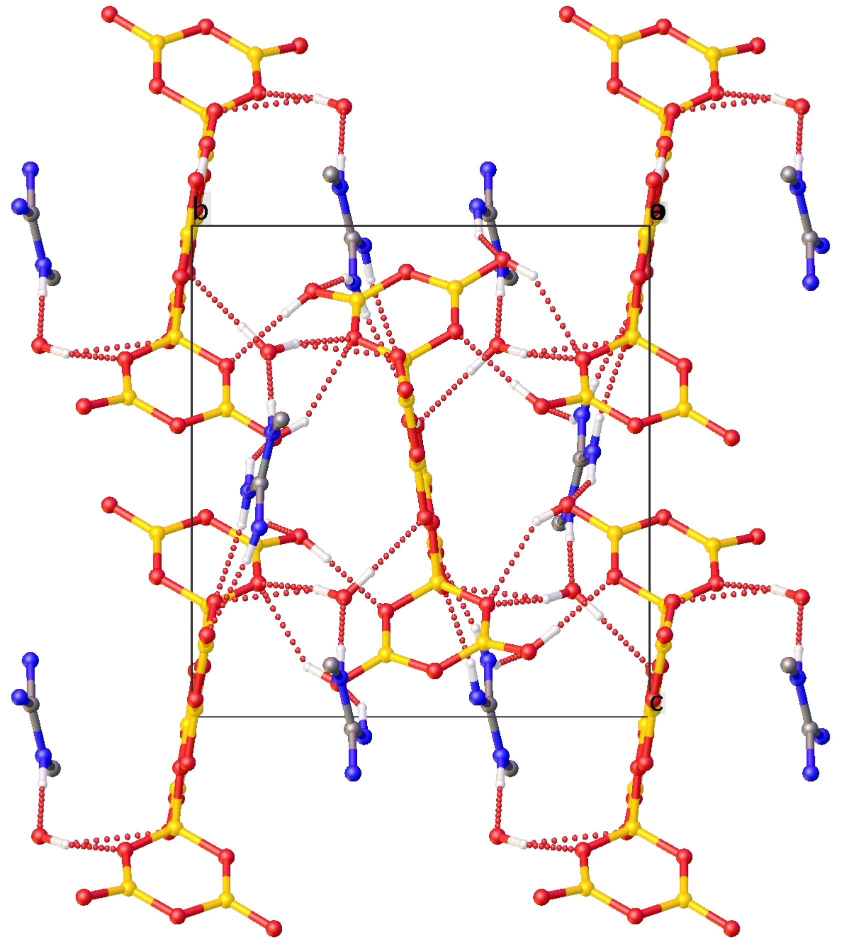

Figure S1: Packing diagram of 2019NCSX0350.

Table S1: Fractional Atomic Coordinates (×10^4) and Equivalent Isotropic Displacement Parameters (Å^2×10^3) for **2019NCSX0350.** U_{eq} is defined as 1/3 of the trace of the orthogonalised U_{ij}.

Atom	x	y	z	U_{eq}
O1	535.3(6)	6452.7(6)	2282.5(5)	10.94(14)
O2	-944.9(6)	5308.1(6)	936.8(5)	12.12(14)
O3	748.8(6)	4242.1(6)	2143.6(5)	10.14(14)
O4	2685.5(6)	5501.8(6)	2657.1(5)	10.37(14)
O5	3313.7(6)	5078.7(6)	4644.2(5)	12.39(14)
O6	983.5(6)	5242.9(6)	3960.2(5)	10.69(14)
O7	-1433.8(6)	7356.8(6)	1324.5(6)	14.04(15)
O8	-675.4(7)	3204.5(6)	683.9(6)	15.97(15)
O9	4958.1(6)	5334.5(6)	3332.8(6)	13.99(15)
O10	1699.2(6)	4907.2(7)	5936.8(5)	13.75(15)
B1	1244.2(9)	5363.8(9)	2740.4(8)	9.19(18)
B2	-582.0(9)	6383.4(9)	1510.8(8)	10.58(19)
B3	-259.3(10)	4243.6(9)	1266.7(8)	11.08(19)
B4	3646.6(10)	5306.3(9)	3537.1(8)	10.17(18)
B5	1965.1(10)	5075.7(9)	4833.9(8)	10.46(19)
O11	2806.5(8)	8321.6(8)	2567.4(7)	26.06(19)
N1	5096.0(8)	8241.1(8)	4207.5(7)	14.88(17)
N2	3506.4(8)	8761.8(9)	5400.5(7)	17.74(18)
N3	5713.6(8)	8524.5(8)	6149.4(7)	15.86(17)
C1	4775.6(9)	8507.1(8)	5250.4(8)	12.74(18)
C2	6473.3(9)	8045.3(9)	3947.2(8)	16.04(19)

Table S2: Anisotropic Displacement Parameters (×10^4) for **2019NCSX0350**. The anisotropic displacement factor exponent takes the form: $-2\pi^2[h^2a^{*2} \times U_{11}+ ... +2hka^* \times b^* \times U_{12}]$$

**Atom**	***U*11**	***U*22**	***U*33**	***U*23**	***U*13**	***U*12**
O1	10.3(3)	9.4(3)	12.2(3)	-0.4(2)	-3.1(2)	0.4(2)
O2	11.8(3)	11.0(3)	12.4(3)	-0.5(2)	-3.9(2)	0.4(2)
O3	9.9(3)	8.9(3)	11.0(3)	0.3(2)	-1.8(2)	-0.1(2)
O4	7.7(3)	14.3(3)	8.9(3)	1.4(2)	-0.1(2)	-0.5(2)
O5	7.0(3)	20.7(3)	9.1(3)	1.9(2)	-0.6(2)	0.4(2)
O6	6.8(3)	16.4(3)	8.7(3)	0.1(2)	0.2(2)	0.2(2)
O7	12.6(3)	11.3(3)	16.9(3)	-2.4(3)	-5.0(2)	2.1(2)
O8	17.8(3)	11.1(3)	17.3(3)	-0.6(3)	-5.6(3)	-0.2(3)
O9	7.7(3)	23.5(4)	10.5(3)	2.7(3)	-0.4(2)	0.0(2)
O10	8.0(3)	23.7(4)	9.4(3)	0.6(2)	0.3(2)	-0.4(2)
B1	7.6(4)	10.8(4)	8.8(4)	0.3(3)	-0.9(3)	-0.2(3)
B2	9.4(4)	12.0(4)	10.1(4)	1.4(3)	0.0(3)	-0.3(3)
B3	10.8(4)	11.9(5)	10.5(4)	0.9(3)	0.9(3)	-1.0(3)
B4	9.4(4)	10.2(4)	10.7(4)	0.1(3)	0.4(3)	-0.2(3)
B5	8.6(4)	11.9(4)	10.7(4)	-0.6(3)	0.0(3)	0.0(3)
O11	25.8(4)	22.6(4)	26.8(4)	9.1(3)	-11.1(3)	-8.3(3)
N1	13.4(4)	19.5(4)	11.2(4)	-1.6(3)	-0.9(3)	2.5(3)
N2	11.2(4)	27.8(5)	13.8(4)	-2.7(3)	-0.9(3)	3.2(3)
N3	10.7(4)	25.2(4)	11.5(4)	-1.5(3)	0.1(3)	2.6(3)
C1	12.9(4)	12.1(4)	13.0(4)	0.8(3)	0.3(3)	0.1(3)
C2	15.7(4)	18.3(4)	14.5(4)	-0.9(3)	3.6(3)	1.4(3)

Table S3: Bond Lengths in Å for **2019NCSX0350**.

**Atom**	**Atom**	**Length/Å**
O1	B1	1.4558(11)
O1	B2	1.3626(11)
O2	B2	1.3811(11)
O2	B3	1.3821(12)
O3	B1	1.4687(11)
O3	B3	1.3608(11)
O4	B1	1.4622(10)
O4	B4	1.3497(11)
O5	B4	1.3958(11)
O5	B5	1.3895(11)
O6	B1	1.4862(11)
O6	B5	1.3532(11)
O7	B2	1.3637(11)
O8	B3	1.3651(12)
O9	B4	1.3578(11)
O10	B5	1.3597(11)
N1	C1	1.3282(12)
N1	C2	1.4572(12)
N2	C1	1.3286(12)
N3	C1	1.3342(12)

Table S4: Bond Angles in ° for **2019NCSX0350**.

Atom	Atom	Atom	Angle/°
B2	O1	B1	122.15(7)
B2	O2	B3	118.68(7)
B3	O3	B1	122.94(7)
B4	O4	B1	124.00(7)
B5	O5	B4	118.72(7)
B5	O6	B1	123.57(7)
O1	B1	O3	112.18(7)
O1	B1	O4	109.63(7)
O1	B1	O6	107.38(7)
O3	B1	O6	107.44(7)
O4	B1	O3	109.80(7)
O4	B1	O6	110.37(7)
O1	B2	O2	121.66(8)
O1	B2	O7	121.13(8)
O7	B2	O2	117.10(8)
O3	B3	O2	121.18(8)
O3	B3	O8	122.60(8)
O8	B3	O2	116.20(8)
O4	B4	O5	121.19(8)
O4	B4	O9	118.94(8)
O9	B4	O5	119.87(8)
O6	B5	O5	121.09(8)
O6	B5	O10	122.58(8)
O10	B5	O5	116.33(8)
C1	N1	C2	123.39(8)
N1	C1	N2	119.65(8)
N1	C1	N3	120.80(8)
N2	C1	N3	119.55(8)

Table S5: Torsion Angles in ° for **2019NCSX0350**.

Atom	Atom	Atom	Atom	Angle/°
B1	O1	B2	O2	-13.84(13)
B1	O1	B2	O7	162.17(8)
B1	O3	B3	O2	-4.55(12)
B1	O3	B3	O8	177.13(8)
B1	O4	B4	O5	-6.99(13)
B1	O4	B4	O9	173.07(8)
B1	O6	B5	O5	3.89(13)
B1	O6	B5	O10	-176.69(8)
B2	O1	B1	O3	9.07(11)
B2	O1	B1	O4	131.33(8)
B2	O1	B1	O6	-108.75(8)
B2	O2	B3	O3	0.46(13)
B2	O2	B3	O8	178.88(7)
B3	O2	B2	O1	8.68(13)
B3	O2	B2	O7	-167.49(8)
B3	O3	B1	O1	-0.07(11)
B3	O3	B1	O4	-122.23(8)
B3	O3	B1	O6	117.72(8)
B4	O4	B1	O1	129.99(8)
B4	O4	B1	O3	-106.35(9)
B4	O4	B1	O6	11.91(11)
B4	O5	B5	O6	2.48(13)
B4	O5	B5	O10	-176.98(8)
B5	O5	B4	O4	-1.03(13)
B5	O5	B4	O9	178.91(8)
B5	O6	B1	O1	-129.81(8)
B5	O6	B1	O3	109.33(9)
B5	O6	B1	O4	-10.36(11)
C2	N1	C1	N2	175.01(9)
C2	N1	C1	N3	-4.83(14)

Table S6: Hydrogen Fractional Atomic Coordinates (×10^4) and Equivalent Isotropic Displacement Parameters ($Å^2$×10^3) for **2019NCSX0350**. U_{eq} is defined as 1/3 of the trace of the orthogonalised U_{ij}.

Atom	x	y	z	U_{eq}
H7	-1194(16)	7943(15)	1771(14)	33(4)
H8	-360(16)	2543(16)	1011(14)	36(4)
H9	5443(17)	5197(15)	3934(15)	38(4)
H10	851(18)	4890(16)	5983(14)	39(4)
H11A	2541(16)	8896(16)	2062(14)	39(4)
H11B	2191(18)	7746(19)	2401(15)	51(5)
H1	4438(15)	8257(14)	3626(13)	28(4)
H2A	3297(14)	8904(13)	6089(12)	24(3)
H2B	2915(15)	8721(14)	4815(13)	29(4)
H3A	6561(15)	8351(14)	6063(12)	27(3)
H3B	5488(13)	8800(13)	6799(12)	23(3)
H2C	6816.45	7276.73	4299.4	24
H2D	7039.08	8728.44	4253.18	24
H2E	6490.49	7998.96	3113.93	24

Table S7: Hydrogen Bond information for **2019NCSX0350**.

D	H	A	d(D-H)/Å	d(H-A)/Å	d(D-A)/Å	D-H-A/deg
O7	H7	O3[1]	0.845(17)	1.924(17)	2.7674(9)	175.8(16)

D	H	A	d(D-H)/Å	d(H-A)/Å	d(D-A)/Å	D-H-A/deg
O8	H8	O1[2]	0.861(17)	2.350(17)	3.0462(9)	138.3(14)
O9	H9	O5[3]	0.825(17)	1.994(18)	2.8177(9)	177.0(17)
O10	H10	O6[4]	0.855(18)	1.848(18)	2.7022(9)	176.4(17)
O11	H11A	O10[5]	0.883(18)	1.979(18)	2.8564(10)	172.3(15)
O11	H11B	O1	0.89(2)	2.17(2)	3.0428(10)	169.2(16)
O11	H11B	O4	0.89(2)	2.51(2)	3.0795(10)	122.8(15)
N1	H1	O11	0.895(15)	1.943(15)	2.8295(11)	170.2(14)
N2	H2A	O4[6]	0.868(14)	2.101(15)	2.9624(10)	171.5(13)
N2	H2B	O8[1]	0.858(15)	2.322(15)	3.0379(11)	141.1(13)
N3	H3A	O7[7]	0.884(15)	2.140(15)	2.9954(10)	162.8(13)
N3	H3B	O9[6]	0.870(14)	2.146(15)	3.0132(10)	174.1(13)

----

[1]- x,1/2+y,1/2-z; [2]-x,-1/2+y,1/2-z; [3]1-x,1-y,1-z; [4]-x,1-y,1-z; [5]+x,3/2-y,-1/2+z; [6]+x,3/2-y,1/2+z; [7]1+x,3/2-y,1/2

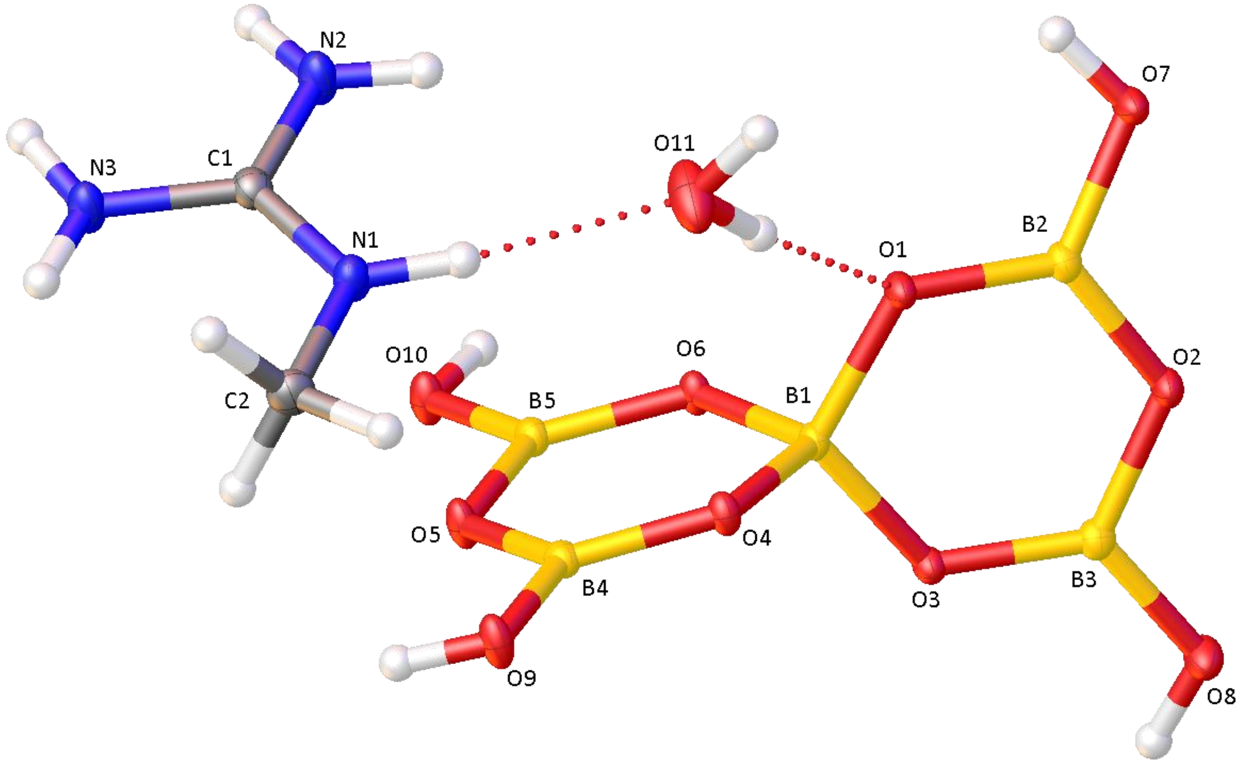

Citations

CrysAlisPro Software System, Rigaku Oxford Diffraction, (2019).

O.V. Dolomanov and L.J. Bourhis and R.J. Gildea and J.A.K. Howard and H. Puschmann, Olex2: A complete structure solution, refinement and analysis program, *J. Appl. Cryst.*, (2009), **42**, 339-341.

Sheldrick, G.M., Crystal structure refinement with ShelXL, *Acta Cryst.*, (2015), **C27**, 3-8.

Sheldrick, G.M., ShelXT-Integrated space-group and crystal-structure determination, *Acta Cryst.*, (2015), **A71**, 3-8.

ncs | UK National Crystallography Service
www.ncs.ac.uk | info@ncs.ac.uk | 023 8059 6722

$[C(NH_2)_2(NH\{NH_2\})][B_5O_6(OH)_4]$ (2)

Submitted by: **Mike A Beckett / Tom Rixon**
Bangor University
Solved by: **Peter N Horton**
Sample ID: **MAB/TR/AmGuan B5**

Crystal Data and Experimental

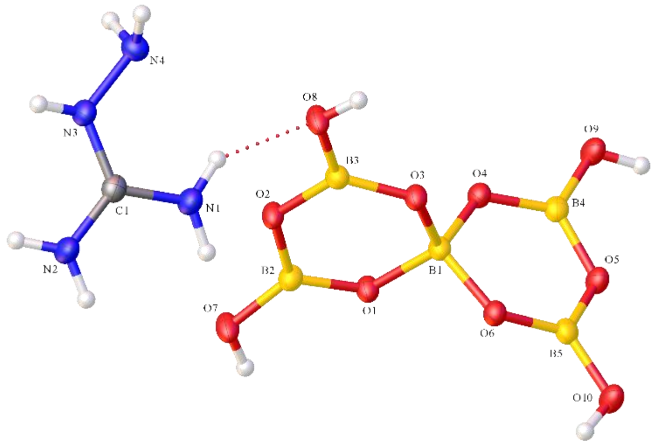

Experimental. Single colourless block-shaped crystals of **2019NCS0257** were recrystallised from water. A suitable crystal 0.250×0.090×0.040 mm³ was selected and mounted on a MITIGEN holder in perfluoroether oil on a Rigaku 007HF equipped with Varimax confocal mirrors and an AFC11 goniometer and HyPix 6000 detector diffractometer. The crystal was kept at a steady T = 100(2) K during data collection. The structure was solved with the **ShelXT** (Sheldrick, 2015) structure solution program using the Intrinsic Phasing solution method and by using **Olex2** (Dolomanov et al., 2009) as the graphical interface. The model was refined with version 2018/3 of **ShelXL** (Sheldrick, 2015) using Least Squares minimisation.

Crystal Data. $CH_{11}B_5N_4O_{10}$, M_r = 293.19, triclinic, P-1 (No. 2), a = 7.4870(2) Å, b = 8.5076(2) Å, c = 9.6502(2) Å, α = 93.906(2)°, β = 98.470(2)°, γ = 96.457(2)°, V = 601.88(3) Å³, T = 100(2) K, Z = 2, Z' = 1, $\mu(CuK_\alpha)$ = 1.341 mm⁻¹, 11885 reflections measured, 2142 unique (R_{int} = 0.0404) which were used in all calculations. The final wR_2 was 0.0827 (all data) and R_1 was 0.0294 (I > 2(I)).

**Compound**	**2019NCS0257**
Formula	$CH_{11}B_5N_4O_{10}$
$D_{calc.}$/ g cm^{-3}	1.618
μ/mm^{-1}	1.341
Formula Weight	293.19
Colour	colourless
Shape	block
Size/mm^3	0.250×0.090×0.040
T/K	100(2)
Crystal System	triclinic
Space Group	P-1
a/Å	7.4870(2)
b/Å	8.5076(2)
c/Å	9.6502(2)
α/°	93.906(2)
β/°	98.470(2)
γ/°	96.457(2)
V/Å^3	601.88(3)
Z	2
Z'	1
Wavelength/Å	1.54178
Radiation type	CuK_α
Θ_{min}/°	4.650
Θ_{max}/°	68.215
Measured Refl.	11885
Independent Refl.	2142
Reflections with I > 2(I)	2060
R_{int}	0.0404
Parameters	225
Restraints	0
Largest Peak	0.201
Deepest Hole	-0.236
GooF	1.040
wR_2 (all data)	0.0827
wR_2	0.0818
R_1 (all data)	0.0302
R_1	0.0294

Structure Quality Indicators

**Reflections:**	d min (Cu) 0.83	I/σ 54.1	Rint 4.04%	complete 98% (IUCr) 98%
**Refinement:**	Shift -0.001	Max Peak 0.2	Min Peak -0.2	GooF 1.040

A colourless block-shaped crystal with dimensions 0.250×0.090×0.040 mm^3 was mounted on a MITIGEN holder in perfluoroether oil. Data were collected using a Rigaku 007HF equipped with Varimax confocal mirrors and an AFC11 goniometer and HyPix 6000 detector diffractometer equipped with an Oxford Cryosystems low-temperature device operating at T = 100(2) K.

Data were measured using profile data from ω-scans 0.5 ° per frame for 0.5 s using CuK_α radiation (Rotating Anode, 40.0 kV, 30.0 mA). The total number of runs and images was based on the strategy calculation from the program **CrysAlisPro** (Rigaku, V1.171.40.45a, 2019). The maximum resolution that was achieved was Θ = 68.215° (0.83 Å).

The diffraction pattern was indexed. The total number of runs and images was based on the strategy calculation from the program **CrysAlisPro** (Rigaku, V1.171.40.45a, 2019) and the unit cell was refined using

CrysAlisPro (Rigaku, V1.171.40.45a, 2019) on 10327 reflections, 87% of the observed reflections.

Data reduction, scaling and absorption corrections were performed using **CrysAlisPro** (Rigaku, V1.171.40.45a, 2019). The final completeness is 98.00 % out to 68.215° in Θ. A multi-scan absorption correction was performed using CrysAlisPro 1.171.40.45a (Rigaku Oxford Diffraction, 2019) using spherical harmonics as implemented in SCALE3 ABSPACK. The absorption coefficient μ of this material is 1.341 mm^{-1} at this wavelength (λ = 1.54178 Å) and the minimum and maximum transmissions are 0.801 and 1.000.

The structure was solved and the space group *P*-1 (# 2) determined by the **ShelXT** (Sheldrick, 2015) structure solution program using Intrinsic Phasing and refined by Least Squares using version 2018/3 of **ShelXL** (Sheldrick, 2015). All non-hydrogen atoms were refined anisotropically.

_exptl_absorpt_process_details: CrysAlisPro 1.171.40.45a (Rigaku Oxford Diffraction, 2019) using spherical harmonics as implemented in SCALE3 ABSPACK.

There is a single molecule in the asymmetric unit, which is represented by the reported sum formula. In other words: Z is 2 and Z' is 1.

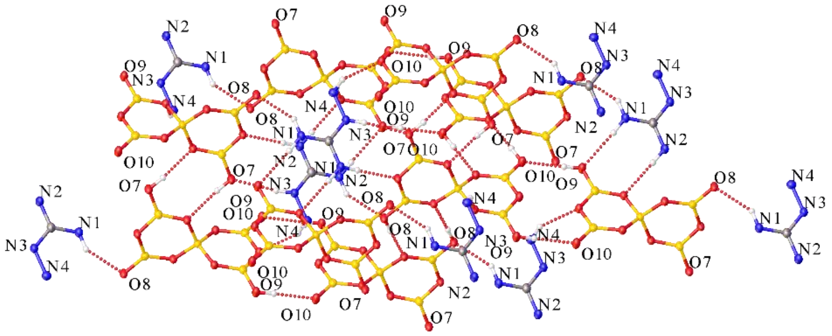

Figure S2: The following hydrogen bonding interactions with a maximum D-D distance of 3.7 Å and a minimum angle of 120 ° are present in **2019NCS0257**: O7–O1_1: 2.776 Å, O10–O6_2: 2.733 Å, O8–O3_3: 2.74 Å, O9–O10_4: 2.829 Å, N3–O7_6: 2.998 Å, N1–O8: 2.898 Å, N1–O9_5: 3.075 Å, N4–O4_8: 3.182 Å, N4–O5_7: 3.168 Å, N2–O4_5: 2.952 Å, N2–O2_6: 2.999 Å, O7–O6_1: 3.141 Å, O8–O6_3: 3.569 Å, O10–O1_2: 3.432 Å, O10–O3_2: 3.651 Å, N1–O4_5: 3.655 Å, N2–O3_5: 3.232 Å, N2–O7_6: 3.53 Å, N3–O2_6: 3.654 Å, N4–O1_8: 3.66 Å, N4–O5_3: 3.226 Å.

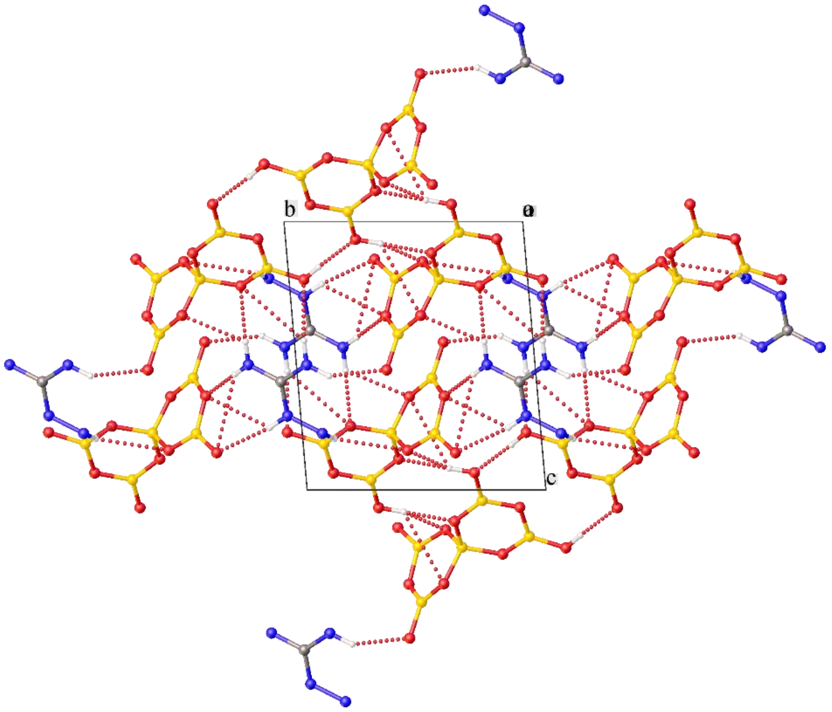

Figure S3: Packing diagram of 2019NCS0257.

Table S8: Fractional Atomic Coordinates (×10^4) and Equivalent Isotropic Displacement Parameters (Å^2×10^3) for **2019NCS0257.** U_{eq} is defined as 1/3 of the trace of the orthogonalised U_{ij}.

Atom	x	y	z	U_{eq}
O(1)	3911.8(10)	5658.2(9)	8518.1(8)	19.3(2)
O(2)	4033.6(11)	3918.3(9)	6492.3(8)	20.5(2)
O(3)	1506.5(10)	5384.6(9)	6512.3(8)	18.4(2)
O(4)	2664.6(10)	7954.6(9)	7617.0(8)	18.6(2)
O(5)	971.4(11)	8839.7(9)	9360.9(8)	20.3(2)
O(6)	922.7(10)	6060.7(9)	8836.9(8)	18.3(2)
O(7)	5900.4(11)	3658.9(10)	8605.8(9)	24.2(2)
O(8)	1835.2(12)	3759.9(10)	4482.1(8)	23.2(2)
O(9)	2179.6(12)	10624.5(10)	7848.4(9)	23.8(2)
O(10)	-582.7(11)	7072.2(10)	10655.0(9)	23.9(2)
B(1)	2255.1(17)	6274.3(14)	7868.9(13)	17.2(3)
B(2)	4621.0(17)	4446.4(15)	7896.7(13)	19.3(3)
B(3)	2429.7(17)	4376.5(14)	5822.2(13)	18.2(3)
B(4)	1931.7(17)	9133.7(15)	8272.1(13)	18.4(3)
B(5)	437.2(17)	7289.3(15)	9611.7(13)	18.9(3)
N(1)	2239.8(14)	428.3(12)	4662.1(11)	23.0(2)
N(2)	3308.3(15)	-2011.8(12)	4673.1(12)	25.4(2)
N(3)	3448.4(14)	-553.7(12)	2772.0(10)	21.6(2)
N(4)	3110.4(15)	836.1(12)	2115.3(10)	22.2(2)
C(1)	2997.9(15)	-703.7(13)	4043.8(12)	19.6(2)

Table S9: Anisotropic Displacement Parameters (×10[4]) **2019NCS0257.** The anisotropic displacement factor exponent takes the form: $-2\pi^2[h^2a^{*2} \times U_{11} + ... +2hka^* \times b^* \times U_{12}]$

Atom	U_{11}	U_{22}	U_{33}	U_{23}	U_{13}	U_{12}
O(1)	19.7(4)	21.6(4)	16.2(4)	-2.4(3)	-0.4(3)	6.9(3)
O(2)	21.7(4)	23.9(4)	16.0(4)	-2.9(3)	0.3(3)	10.0(3)
O(3)	19.0(4)	20.0(4)	15.7(4)	-2.2(3)	-0.2(3)	6.0(3)
O(4)	20.9(4)	18.6(4)	16.9(4)	-0.4(3)	3.8(3)	4.8(3)
O(5)	24.3(4)	17.6(4)	20.2(4)	-1.1(3)	6.8(3)	5.1(3)
O(6)	19.9(4)	17.7(4)	17.5(4)	-0.6(3)	3.2(3)	4.6(3)
O(7)	26.4(5)	28.3(5)	17.3(4)	-5.0(3)	-3.3(3)	13.4(3)
O(8)	25.5(5)	26.0(4)	17.4(4)	-4.7(3)	-3.3(3)	12.6(3)
O(9)	31.9(5)	18.6(4)	23.8(4)	0.4(3)	11.8(4)	6.6(3)
O(10)	30.4(5)	18.2(4)	26.0(4)	1.0(3)	12.4(4)	6.0(3)
B(1)	18.9(6)	17.9(6)	14.5(6)	-1.0(5)	0.7(5)	5.1(5)
B(2)	18.6(6)	21.9(6)	16.6(6)	-1.9(5)	1.4(5)	3.8(5)
B(3)	20.3(6)	17.6(6)	16.5(6)	0.5(5)	1.5(5)	4.3(5)
B(4)	17.5(6)	19.9(6)	16.9(6)	-1.2(5)	0.0(4)	3.2(5)
B(5)	17.9(6)	20.4(6)	18.2(6)	0.5(5)	0.3(5)	5.7(5)
N(1)	28.1(6)	22.9(5)	19.7(5)	0.5(4)	7.0(4)	8.1(4)
N(2)	34.6(6)	24.0(5)	21.5(5)	2.5(4)	11.1(4)	11.4(4)
N(3)	26.7(5)	21.2(5)	18.1(5)	-0.4(4)	4.8(4)	8.0(4)
N(4)	24.7(5)	22.4(5)	19.3(5)	2.0(4)	1.3(4)	4.8(4)
C(1)	16.7(5)	21.4(6)	19.5(5)	-1.7(4)	1.4(4)	2.1(4)

Table S10: Bond Lengths in Å for **2019NCS0257**.

Atom	Atom	Length/Å
O(1)	B(1)	1.4723(14)
O(1)	B(2)	1.3554(15)
O(2)	B(2)	1.3889(14)
O(2)	B(3)	1.3868(15)
O(3)	B(1)	1.4678(14)
O(3)	B(3)	1.3594(15)
O(4)	B(1)	1.4722(14)
O(4)	B(4)	1.3589(15)
O(5)	B(4)	1.3790(15)
O(5)	B(5)	1.3827(15)

Atom	Atom	Length/Å
O(6)	B(1)	1.4689(14)
O(6)	B(5)	1.3574(15)
O(7)	B(2)	1.3596(16)
O(8)	B(3)	1.3532(15)
O(9)	B(4)	1.3602(15)
O(10)	B(5)	1.3605(15)
N(1)	C(1)	1.3229(15)
N(2)	C(1)	1.3304(16)
N(3)	N(4)	1.4092(14)
N(3)	C(1)	1.3304(15)

Table S11: Bond Angles in ° for **2019NCS0257**.

Atom	Atom	Atom	Angle/°
B(2)	O(1)	B(1)	122.82(9)
B(3)	O(2)	B(2)	118.62(9)
B(3)	O(3)	B(1)	123.66(9)
B(4)	O(4)	B(1)	122.43(9)
B(4)	O(5)	B(5)	119.54(9)
B(5)	O(6)	B(1)	122.92(9)
O(3)	B(1)	O(1)	110.87(9)
O(3)	B(1)	O(4)	107.94(9)
O(3)	B(1)	O(6)	109.11(9)
O(4)	B(1)	O(1)	110.42(9)
O(6)	B(1)	O(1)	106.87(9)
O(6)	B(1)	O(4)	111.65(9)
O(1)	B(2)	O(2)	121.08(10)
O(1)	B(2)	O(7)	122.56(10)

Atom	Atom	Atom	Angle/°
O(7)	B(2)	O(2)	116.34(10)
O(3)	B(3)	O(2)	120.33(10)
O(8)	B(3)	O(2)	117.11(10)
O(8)	B(3)	O(3)	122.56(11)
O(4)	B(4)	O(5)	120.97(10)
O(4)	B(4)	O(9)	118.87(10)
O(9)	B(4)	O(5)	120.16(10)
O(6)	B(5)	O(5)	120.49(10)
O(6)	B(5)	O(10)	122.61(11)
O(10)	B(5)	O(5)	116.90(10)
C(1)	N(3)	N(4)	118.42(10)
N(1)	C(1)	N(2)	120.82(11)
N(1)	C(1)	N(3)	120.23(11)
N(3)	C(1)	N(2)	118.95(10)

Table S12: Torsion Angles in ° for **2019NCS0257**.

Atom	Atom	Atom	Atom	Angle/°
B(1)	O(1)	B(2)	O(2)	12.76(17)
B(1)	O(1)	B(2)	O(7)	-165.67(11)
B(1)	O(3)	B(3)	O(2)	11.49(16)
B(1)	O(3)	B(3)	O(8)	-169.51(10)
B(1)	O(4)	B(4)	O(5)	9.53(16)
B(1)	O(4)	B(4)	O(9)	-171.32(10)
B(1)	O(6)	B(5)	O(5)	10.16(16)
B(1)	O(6)	B(5)	O(10)	-169.95(10)
B(2)	O(1)	B(1)	O(3)	1.06(14)
B(2)	O(1)	B(1)	O(4)	-118.52(11)
B(2)	O(1)	B(1)	O(6)	119.86(10)
B(2)	O(2)	B(3)	O(3)	3.18(16)
B(2)	O(2)	B(3)	O(8)	-175.87(10)
B(3)	O(2)	B(2)	O(1)	-15.14(16)
B(3)	O(2)	B(2)	O(7)	163.38(10)
B(3)	O(3)	B(1)	O(1)	-13.17(14)
B(3)	O(3)	B(1)	O(4)	107.89(11)
B(3)	O(3)	B(1)	O(6)	-130.61(10)
B(4)	O(4)	B(1)	O(1)	-115.99(11)
B(4)	O(4)	B(1)	O(3)	122.68(10)
B(4)	O(4)	B(1)	O(6)	2.76(14)
B(4)	O(5)	B(5)	O(6)	3.04(16)
B(4)	O(5)	B(5)	O(10)	-176.86(9)
B(5)	O(5)	B(4)	O(4)	-12.83(16)
B(5)	O(5)	B(4)	O(9)	168.03(10)
B(5)	O(6)	B(1)	O(1)	108.27(11)
B(5)	O(6)	B(1)	O(3)	-131.80(10)
B(5)	O(6)	B(1)	O(4)	-12.58(14)
N(4)	N(3)	C(1)	N(1)	0.47(16)
N(4)	N(3)	C(1)	N(2)	179.91(10)

Table S13: Hydrogen Fractional Atomic Coordinates (×10[4]) and Equivalent Isotropic Displacement Parameters ($Å^2×10^3$) for **2019NCS0257**. U_{eq} is defined as 1/3 of the trace of the orthogonalised U_{ij}.

Atom	x	y	z	U_{eq}
H(7)	6070(30)	3990(20)	9480(20)	55(5)
H(8)	880(30)	4070(20)	4150(20)	48(5)
H(9)	1670(20)	11280(20)	8315(17)	36(4)
H(10)	-780(30)	6030(30)	10810(20)	53(5)
H(3)	3770(20)	-1340(20)	2327(17)	31(4)
H(1A)	2190(20)	1330(20)	4253(17)	32(4)
H(1B)	2120(20)	402(19)	5565(18)	33(4)
H(2A)	3990(20)	-2612(19)	4318(17)	32(4)
H(2B)	3090(20)	-2044(19)	5531(19)	35(4)
H(4A)	4170(20)	1322(18)	1917(16)	29(4)
H(4B)	2350(20)	567(17)	1319(17)	26(4)

Table S14: Hydrogen Bond information for **2019NCS0257**.

D	H	A	d(D-H)/Å	d(H-A)/Å	d(D-A)/Å	D-H-A/deg
O(7)	H(7)	O(1)[1]	0.85(2)	1.94(2)	2.7763(11)	167.7(19)
O(8)	H(8)	O(3)[2]	0.82(2)	1.92(2)	2.7400(12)	173.6(18)
O(9)	H(9)	O(10)[3]	0.851(18)	1.979(18)	2.8294(12)	175.9(16)
O(10)	H(10)	O(6)[4]	0.91(2)	1.83(2)	2.7334(11)	174.2(18)
N(3)	H(3)	O(7)[5]	0.851(17)	2.164(18)	2.9978(13)	166.6(14)
N(1)	H(1A)	O(8)	0.887(17)	2.116(17)	2.8984(14)	146.7(14)
N(1)	H(1B)	O(9)[6]	0.890(17)	2.192(18)	3.0752(13)	171.4(14)
N(2)	H(2A)	O(2)[5]	0.851(17)	2.152(18)	2.9988(13)	173.4(15)
N(2)	H(2B)	O(4)[6]	0.868(18)	2.084(18)	2.9522(13)	177.6(16)
N(4)	H(4A)	O(4)[7]	0.906(17)	2.348(17)	3.1816(13)	153.0(13)
N(4)	H(4B)	O(5)[8]	0.887(16)	2.345(16)	3.1676(13)	154.3(13)

----

[1]1-x,1-y,2-z; [2]-x,1-y,1-z; [3]-x,2-y,2-z; [4]-x,1-y,2-z; [5]1-x,-y,1-z; [6]+x,-1+y,+z; [7]1-x,1-y,1-z; [8]+x,-1+y,-1+z

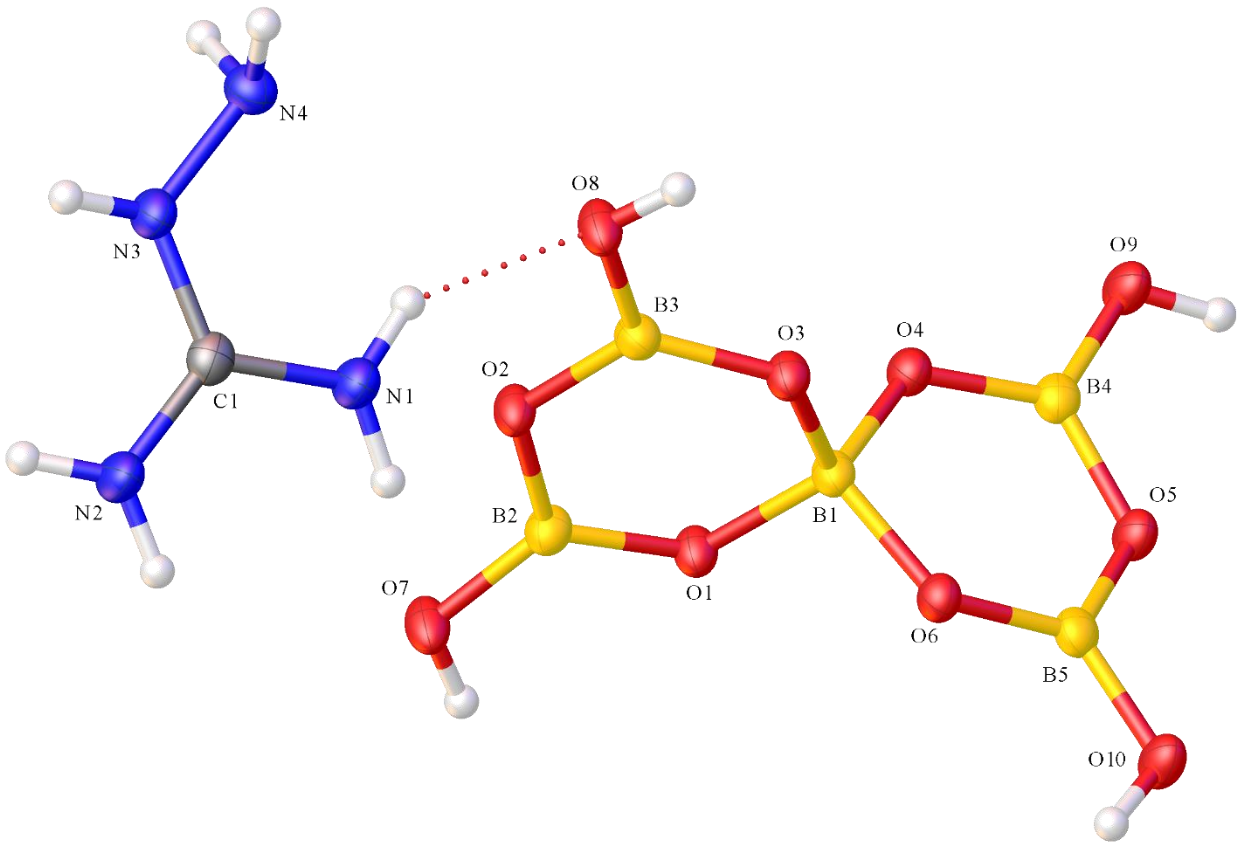

Citations

CrysAlisPro Software System, Rigaku Oxford Diffraction, (2019).

O.V. Dolomanov and L.J. Bourhis and R.J. Gildea and J.A.K. Howard and H. Puschmann, Olex2: A complete structure solution, refinement and analysis program, *J. Appl. Cryst.*, (2009), **42**, 339-341.

Sheldrick, G.M., Crystal structure refinement with ShelXL, *Acta Cryst.*, (2015), **C27**, 3-8.

Sheldrick, G.M., ShelXT-Integrated space-group and crystal-structure determination, *Acta Cryst.*, (2015), **A71**, 3-8.

$[C(NH_2)_2(NMe_2)][B_5O_6(OH)_4]$ (**3**)

Submitted by: **Mike A Beckett / Tom Rixon**
Bangor University
Solved by: **Peter N Horton**
Sample ID: **MAB/TR/Me2Gaun B5**

Crystal Data and Experimental

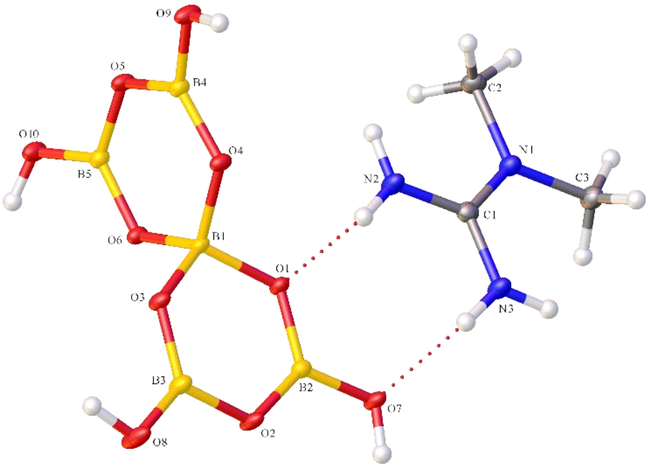

Experimental. Single colourless plate-shaped crystals of **2019ncs0334** were recrystallised. A suitable crystal 0.280 × 0.150 × 0.030 mm³ was selected and mounted on a MITIGEN holder in perfluoroether oil on a Rigaku FRE+ equipped with VHF Varimax confocal mirrors and an AFC12 goniometer and HyPix 6000 detector diffractometer. The crystal was kept at a steady T = 100(2) K during data collection. The structure was solved with the **ShelXT** (Sheldrick, 2015) structure solution program using the Intrinsic Phasing solution method and by using **Olex2** (Dolomanov et al., 2009) as the graphical interface. The model was refined with version 2018/3 of **ShelXL** (Sheldrick, 2015) using Least Squares minimisation.

Crystal Data. $C_3H_{14}B_5N_3O_{10}$, M_r = 306.22, monoclinic, $P2_1/c$ (No. 14), a = 9.9747(2) Å, b = 11.2563(3) Å, c = 11.6174(3) Å, β = 96.084(2)°, α = γ = 90°, V = 1297.03(5) Å³, T = 100(2) K, Z = 4, Z' = 1, $\mu(MoK_\alpha)$ = 0.142 mm⁻¹, 18168 reflections measured, 2969 unique (R_{int} = 0.0191) which were used in all calculations. The final wR_2 was 0.0811 (all data) and R_1 was 0.0280 (I > 2(I)).

**Compound**	**2019ncs0334**
Formula	$C_3H_{14}B_5N_3O_{10}$
$D_{calc.}$/ g cm^{-3}	1.568
μ/mm^{-1}	0.142
Formula Weight	306.22
Colour	colourless
Shape	plate
Size/mm^3	0.280×0.150×0.030
T/K	100(2)
Crystal System	monoclinic
Space Group	$P2_1/c$
a/Å	9.9747(2)
b/Å	11.2563(3)
c/Å	11.6174(3)
$\alpha/^\circ$	90
$\beta/^\circ$	96.084(2)
$\gamma/^\circ$	90
V/Å^3	1297.03(5)
Z	4
Z'	1
Wavelength/Å	0.71075
Radiation type	MoK_α
$\Theta_{min}/^\circ$	2.526
$\Theta_{max}/^\circ$	27.483
Measured Refl.	18168
Independent Refl.	2969
Reflections with I > 2(I)	2722
R_{int}	0.0191
Parameters	224
Restraints	0
Largest Peak	0.268
Deepest Hole	-0.227
GooF	1.035
wR_2 (all data)	0.0811
wR_2	0.0793
R_1 (all data)	0.0306
R_1	0.0280

Structure Quality Indicators

**Reflections:**	d min (Mo) 0.77	I/σ 81.3	Rint 1.91%	complete 100% (IUCr) 100%
**Refinement:**	Shift 0.000	Max Peak 0.3	Min Peak -0.2	GooF 1.035

A colourless plate-shaped crystal with dimensions 0.280×0.150×0.030 mm^3 was mounted on a MITIGEN holder in perfluoroether oil. Data were collected using a Rigaku FRE+ equipped with VHF Varimax confocal mirrors and an AFC12 goniometer and HyPix 6000 detector diffractometer equipped with an Oxford Cryosystems low-temperature device operating at T = 100(2) K.

Data were measured using profile data from ω-scans 0.5 ° per frame for 2.0 s using MoK_α radiation (Rotating Anode, 45.0 kV, 55.0 mA). The total number of runs and images was based on the strategy calculation from the program **CrysAlisPro** (Rigaku, V1.171.40.45a, 2019). The maximum resolution that was achieved was Θ = 27.483° (0.77 Å).

The diffraction pattern was indexed. The total number of runs and images was based on the strategy calculation from the program **CrysAlisPro** (Rigaku, V1.171.40.45a, 2019) and the unit cell was refined using **CrysAlisPro** (Rigaku, V1.171.40.45a, 2019) on 10774 reflections, 59% of the observed reflections.

Data reduction, scaling and absorption corrections were performed using **CrysAlisPro** (Rigaku, V1.171.40.45a, 2019). The final completeness is 99.90 % out to 27.483° in Θ. A multi-scan absorption correction was performed using CrysAlisPro 1.171.40.45a (Rigaku Oxford Diffraction, 2019) using spherical harmonics as implemented in SCALE3 ABSPACK. The absorption coefficient μ of this material is 0.142 mm^{-1} at this wavelength (λ = 0.71075 Å) and the minimum and maximum transmissions are 0.643 and 1.000.

The structure was solved and the space group $P2_1/c$ (# 14) determined by the **ShelXT** (Sheldrick, 2015) structure solution program using Intrinsic Phasing and refined by Least Squares using version 2018/3 of **ShelXL** (Sheldrick, 2015). All non-hydrogen atoms were refined anisotropically. Most hydrogen atom positions were calculated geometrically and refined using the riding model, but some hydrogen atoms were refined freely.

_exptl_absorpt_process_details: CrysAlisPro 1.171.40.45a (Rigaku Oxford Diffraction, 2019) using spherical harmonics as implemented in SCALE3 ABSPACK.

There is a single molecule in the asymmetric unit, which is represented by the reported sum formula. In other words: Z is 4 and Z' is 1.

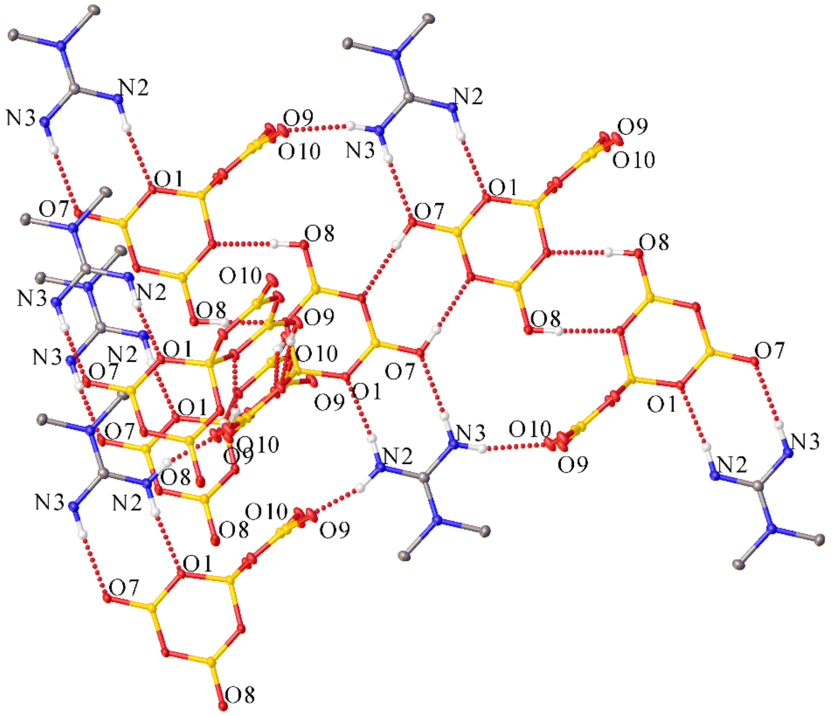

Figure S4: The following hydrogen bonding interactions with a maximum D-D distance of 3.04 Å and a minimum angle of 120 ° are present in **2019ncs0334**: O7–O2_*1: 2.759 Å, O8–O3*_2: 2.735 Å, O9–O6_*3: 2.791 Å, O10–O4*_4: 2.857 Å, N2–O1: 2.898 Å, N2–O10_*5: 2.919 Å, N3–O7: 2.97 Å, N3–O9*_6: 3.017 Å.

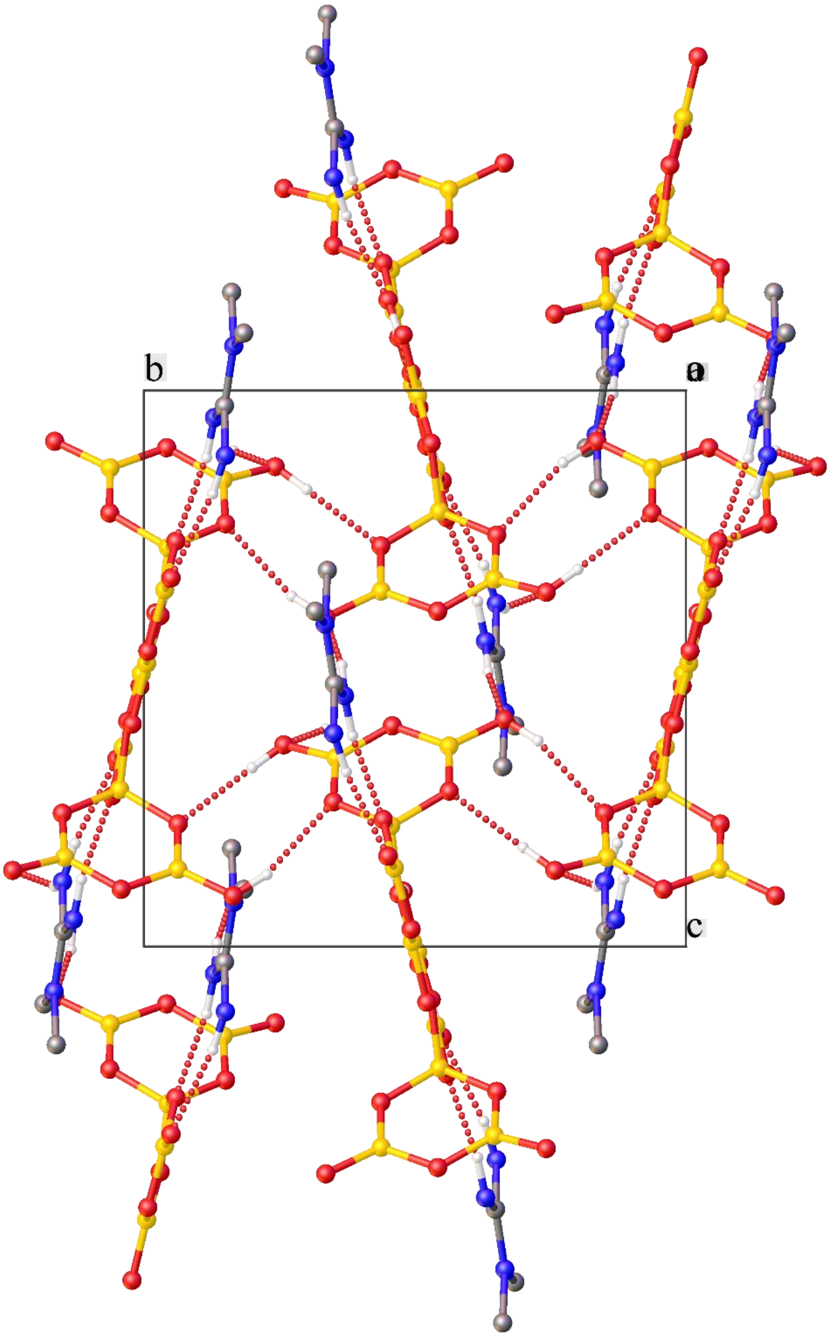

Figure S5: Packing diagram of 2019ncs0334.

Table S15: Fractional Atomic Coordinates (×10[4]) and Equivalent Isotropic Displacement Parameters (Å^2×10^3) for **2019ncs0334**. U_{eq} is defined as 1/3 of the trace of the orthogonalised U_{ij}.

Atom	x	y	z	U_{eq}
O(1)	2668.8(6)	5588.7(6)	7711.1(5)	10.55(14)
O(2)	3336.6(6)	5072.4(7)	9685.8(5)	15.08(16)
O(3)	1003.4(6)	5298.6(6)	9048.9(5)	12.43(15)
O(4)	492.2(6)	6506.3(6)	7398.1(5)	11.63(14)
O(5)	-996.5(6)	5419.6(6)	6030.2(6)	13.16(15)
O(6)	726.3(6)	4369.3(6)	7204.6(5)	11.60(15)
O(7)	4948.5(7)	5500.9(6)	8372.8(6)	13.91(15)
O(8)	1742.0(7)	4781.8(8)	10997.5(6)	22.00(18)
O(9)	-1427.0(7)	7420.9(6)	6371.5(6)	15.74(16)
O(10)	-904.5(7)	3353.8(6)	5915.8(6)	15.68(16)
B(1)	1227.3(10)	5447.2(9)	7825.0(8)	10.11(19)
B(2)	3639.9(10)	5381.7(9)	8585.6(9)	10.81(19)
B(3)	1983.9(10)	5050.2(10)	9906.1(9)	13.8(2)
B(4)	-609.6(10)	6462.1(9)	6604.5(9)	11.9(2)
B(5)	-356.4(10)	4374.3(9)	6392.5(9)	11.6(2)
N(1)	5049.5(8)	6656.9(7)	4200.9(7)	13.68(17)
N(2)	3479.1(8)	6301.3(8)	5489.9(7)	15.67(18)
N(3)	5716.3(8)	6501.8(8)	6169.9(7)	15.72(18)
C(1)	4746.8(9)	6485.7(8)	5276.9(8)	12.45(18)
C(2)	3979.2(9)	6617.5(9)	3236.5(8)	15.07(19)
C(3)	6452.1(9)	6841.5(9)	3970.0(8)	15.89(19)

Table S16: Anisotropic Displacement Parameters (×10[4]) **2019ncs0334**. The anisotropic displacement factor exponent takes the form: $-2\pi^2[h^2a^{*2} \times U_{11}+ ... +2hka^* \times b^* \times U_{12}]$

Atom	U_{11}	U_{22}	U_{33}	U_{23}	U_{13}	U_{12}
O(1)	7.5(3)	14.4(3)	9.3(3)	1.0(2)	-0.8(2)	-0.6(2)
O(2)	7.2(3)	27.2(4)	10.4(3)	4.2(3)	-0.8(2)	0.8(3)
O(3)	7.2(3)	19.6(3)	10.3(3)	1.6(2)	-0.2(2)	0.7(2)
O(4)	9.9(3)	10.8(3)	13.2(3)	-0.2(2)	-3.2(2)	0.4(2)
O(5)	11.9(3)	12.2(3)	13.9(3)	0.1(2)	-5.3(2)	0.1(2)
O(6)	9.7(3)	10.5(3)	13.6(3)	0.5(2)	-3.2(2)	-0.3(2)
O(7)	7.7(3)	22.8(4)	10.8(3)	3.5(3)	-1.1(2)	0.1(2)
O(8)	9.2(3)	44.6(5)	12.0(3)	7.0(3)	0.2(2)	1.0(3)
O(9)	13.3(3)	12.4(3)	19.6(3)	-1.4(3)	-6.9(3)	1.7(2)
O(10)	14.4(3)	11.9(3)	19.0(3)	0.6(3)	-6.4(3)	-1.0(2)
B(1)	7.5(4)	12.2(5)	10.1(4)	0.7(3)	-1.4(3)	-0.4(3)
B(2)	9.6(4)	11.4(4)	11.0(4)	0.3(3)	-0.5(3)	0.0(3)
B(3)	8.9(4)	19.9(5)	12.2(4)	1.3(4)	-0.3(3)	0.7(4)
B(4)	9.7(4)	13.6(5)	11.9(4)	1.4(4)	-0.8(3)	-0.6(4)
B(5)	9.4(4)	13.5(5)	11.6(4)	0.7(4)	-0.1(3)	-0.9(3)
N(1)	10.8(4)	18.0(4)	12.0(4)	1.6(3)	0.1(3)	-0.6(3)
N(2)	10.9(4)	24.6(4)	11.1(4)	2.6(3)	-0.6(3)	-2.0(3)
N(3)	10.8(4)	24.0(4)	11.9(4)	2.5(3)	-0.7(3)	-1.3(3)
C(1)	13.0(4)	11.2(4)	12.9(4)	0.1(3)	0.0(3)	0.6(3)
C(2)	15.0(4)	18.4(5)	11.2(4)	1.3(3)	-1.5(3)	0.4(3)
C(3)	11.9(4)	21.1(5)	14.9(4)	2.1(4)	2.6(3)	-0.1(3)

Table S17: Bond Lengths in Å for **2019ncs0334**.

Atom	Atom	Length/Å
O(1)	B(1)	1.4665(11)
O(1)	B(2)	1.3476(12)
O(2)	B(2)	1.3887(12)
O(2)	B(3)	1.4000(12)
O(3)	B(1)	1.4721(11)
O(3)	B(3)	1.3490(12)
O(4)	B(1)	1.4586(11)
O(4)	B(4)	1.3586(11)
O(5)	B(4)	1.3843(12)
O(5)	B(5)	1.3828(12)
O(6)	B(1)	1.4715(11)
O(6)	B(5)	1.3566(12)
O(7)	B(2)	1.3606(12)
O(8)	B(3)	1.3498(12)
O(9)	B(4)	1.3623(12)
O(10)	B(5)	1.3642(12)
N(1)	C(1)	1.3307(12)
N(1)	C(2)	1.4641(11)
N(1)	C(3)	1.4670(11)
N(2)	C(1)	1.3303(12)
N(3)	C(1)	1.3413(12)

Table S18: Bond Angles in ° for **2019ncs0334**.

Atom	Atom	Atom	Angle/°
B(2)	O(1)	B(1)	123.13(7)
B(2)	O(2)	B(3)	118.75(7)
B(3)	O(3)	B(1)	124.35(7)
B(4)	O(4)	B(1)	122.83(7)
B(5)	O(5)	B(4)	118.58(7)
B(5)	O(6)	B(1)	122.76(7)
O(1)	B(1)	O(3)	110.50(7)
O(1)	B(1)	O(6)	109.31(7)
O(4)	B(1)	O(1)	110.06(7)
O(4)	B(1)	O(3)	107.40(7)
O(4)	B(1)	O(6)	112.02(7)
O(6)	B(1)	O(3)	107.51(7)
O(1)	B(2)	O(2)	121.86(8)
O(1)	B(2)	O(7)	118.21(8)
O(7)	B(2)	O(2)	119.92(8)
O(3)	B(3)	O(2)	119.91(8)
O(3)	B(3)	O(8)	123.51(9)
O(8)	B(3)	O(2)	116.58(8)
O(4)	B(4)	O(5)	121.48(8)
O(4)	B(4)	O(9)	122.00(9)
O(9)	B(4)	O(5)	116.45(8)
O(6)	B(5)	O(5)	121.54(8)
O(6)	B(5)	O(10)	122.28(8)
O(10)	B(5)	O(5)	116.14(8)
C(1)	N(1)	C(2)	119.66(8)
C(1)	N(1)	C(3)	120.54(8)
C(2)	N(1)	C(3)	119.77(7)
N(1)	C(1)	N(3)	120.52(8)
N(2)	C(1)	N(1)	120.81(8)
N(2)	C(1)	N(3)	118.67(8)

Table S19: Torsion Angles in ° for **2019ncs0334**.

Atom	Atom	Atom	Atom	Angle/°
B(1)	O(1)	B(2)	O(2)	-3.25(13)
B(1)	O(1)	B(2)	O(7)	177.74(8)
B(1)	O(3)	B(3)	O(2)	8.45(14)
B(1)	O(3)	B(3)	O(8)	-172.06(9)
B(1)	O(4)	B(4)	O(5)	-9.06(13)
B(1)	O(4)	B(4)	O(9)	167.75(8)
B(1)	O(6)	B(5)	O(5)	5.20(13)
B(1)	O(6)	B(5)	O(10)	-172.52(8)
B(2)	O(1)	B(1)	O(3)	11.53(12)
B(2)	O(1)	B(1)	O(4)	129.98(8)
B(2)	O(1)	B(1)	O(6)	-106.59(9)
B(2)	O(2)	B(3)	O(3)	1.60(14)
B(2)	O(2)	B(3)	O(8)	-177.94(9)
B(3)	O(2)	B(2)	O(1)	-4.13(14)
B(3)	O(2)	B(2)	O(7)	174.85(9)

Atom	Atom	Atom	Atom	Angle/°
B(3)	O(3)	B(1)	O(1)	-14.28(12)
B(3)	O(3)	B(1)	O(4)	-134.34(9)
B(3)	O(3)	B(1)	O(6)	104.94(9)
B(4)	O(4)	B(1)	O(1)	128.12(8)
B(4)	O(4)	B(1)	O(3)	-111.55(9)
B(4)	O(4)	B(1)	O(6)	6.29(11)
B(4)	O(5)	B(5)	O(6)	-7.18(13)
B(4)	O(5)	B(5)	O(10)	170.67(8)
B(5)	O(5)	B(4)	O(4)	9.08(13)
B(5)	O(5)	B(4)	O(9)	-167.89(8)
B(5)	O(6)	B(1)	O(1)	-126.65(8)
B(5)	O(6)	B(1)	O(3)	113.38(9)
B(5)	O(6)	B(1)	O(4)	-4.39(11)
C(2)	N(1)	C(1)	N(2)	-1.28(13)
C(2)	N(1)	C(1)	N(3)	179.06(9)
C(3)	N(1)	C(1)	N(2)	-179.06(9)
C(3)	N(1)	C(1)	N(3)	1.28(14)

Table S20: Hydrogen Fractional Atomic Coordinates (×10^4) and Equivalent Isotropic Displacement Parameters (Å^2×10^3) for **2019ncs0334**. U_{eq} is defined as 1/3 of the trace of the orthogonalised U_{ij}.

Atom	x	y	z	U_{eq}
H(2C)	3537.54	5839.08	3218.18	23
H(2D)	4368.9	6747.54	2507.08	23
H(2E)	3314.8	7239.67	3338.68	23
H(3C)	6781.43	7595.92	4313.32	24
H(3D)	6500.38	6862.83	3132.11	24
H(3E)	7011.52	6188.94	4310.36	24
H(2A)	3291(13)	6153(12)	6208(12)	21(3)
H(2B)	2820(15)	6349(13)	4943(13)	27(3)
H(9)	-1174(16)	8004(15)	6783(14)	37(4)
H(3A)	6556(16)	6645(14)	6087(12)	31(4)
H(8)	842(19)	4790(15)	11009(14)	43(4)
H(3B)	5512(13)	6270(13)	6857(12)	22(3)
H(10)	-624(16)	2731(15)	6298(14)	39(4)
H(7)	5490(18)	5311(14)	8952(15)	40(4)

Table S21: Hydrogen Bond information for **2019ncs0334**.

D	H	A	d(D-H)/Å	d(H-A)/Å	d(D-A)/Å	D-H-A/deg
N(2)	H(2A)	O(1)	0.890(14)	2.017(14)	2.8978(10)	170.2(13)
N(2)	H(2B)	O(10)[1]	0.866(15)	2.087(15)	2.9195(10)	161.1(13)
O(9)	H(9)	O(6)[2]	0.835(17)	1.958(17)	2.7911(10)	175.3(16)
N(3)	H(3A)	O(9)[3]	0.869(16)	2.186(16)	3.0171(11)	160.1(13)
O(8)	H(8)	O(3)[4]	0.899(19)	1.837(19)	2.7347(10)	175.3(16)
N(3)	H(3B)	O(7)	0.884(14)	2.091(14)	2.9700(10)	172.2(13)
O(10)	H(10)	O(4)[5]	0.861(17)	2.042(17)	2.8566(9)	157.5(15)
O(7)	H(7)	O(2)[6]	0.845(18)	1.915(18)	2.7589(9)	177.1(17)

----

[1]-x,1-y,1-z; [2]-x,1/2+y,3/2-z; [3]1+x,+y,+z; [4]-x,1-y,2-z; [5]-x,-1/2+y,3/2-z; [6]1-x,1-y,2-z

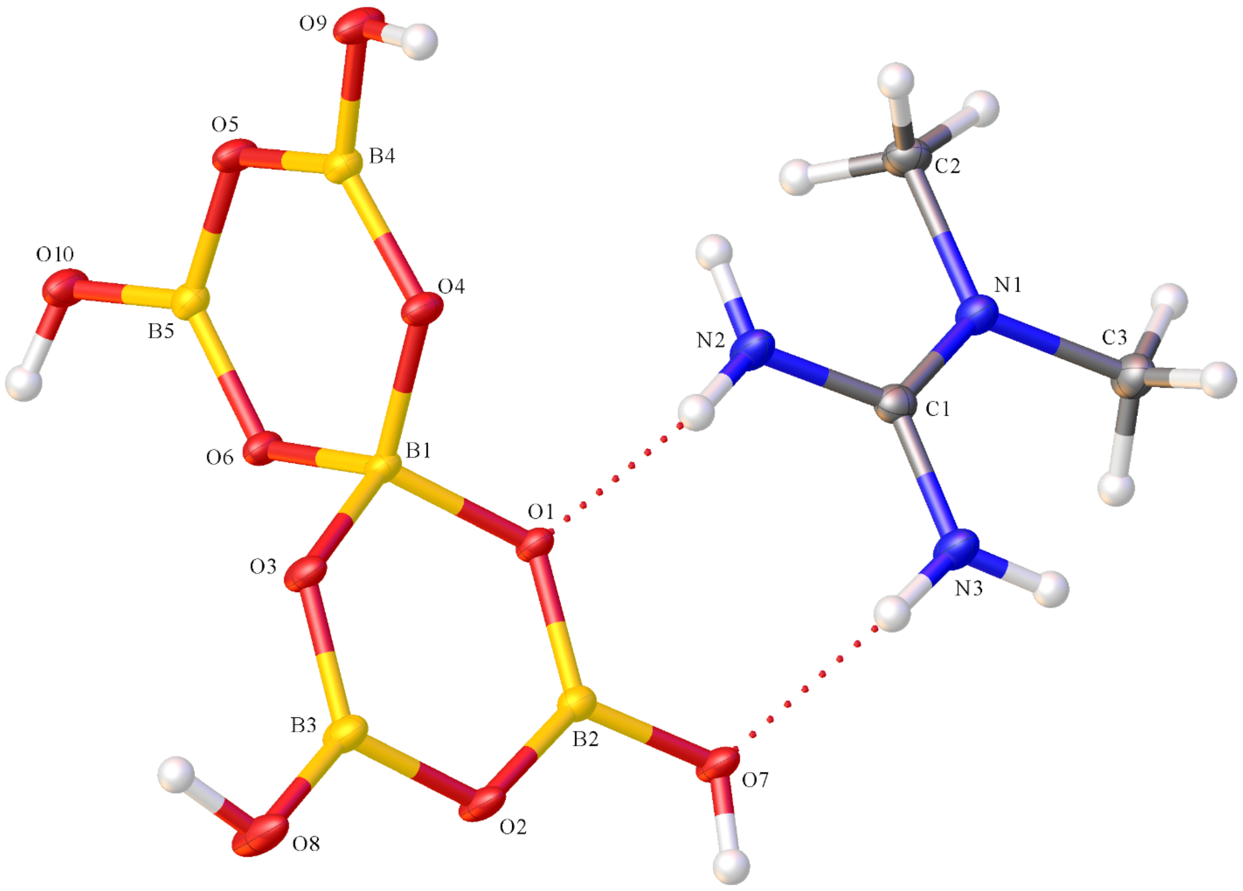

Citations

CrysAlisPro Software System, Rigaku Oxford Diffraction, (2019).

O.V. Dolomanov and L.J. Bourhis and R.J. Gildea and J.A.K. Howard and H. Puschmann, Olex2: A complete structure solution, refinement and analysis program, *J. Appl. Cryst.*, (2009), **42**, 339-341.

Sheldrick, G.M., Crystal structure refinement with ShelXL, *Acta Cryst.*, (2015), **C27**, 3-8.

Sheldrick, G.M., ShelXT-Integrated space-group and crystal-structure determination, *Acta Cryst.*, (2015), **A71**, 3-8.

2011src0322

ncs | UK National Crystallography Service
www.ncs.ac.uk | info@ncs.ac.uk | 023 8059 6722

Submitted by: **Mike A Beckett**
Bangor University
Solved by: **Peter N Horton**
Sample ID: **MAB/JM/1**

R_1=3.73%

$[C(NH_2)(NMe_2)_2][B_5O_6(OH)_4]$ (**4**)

Crystal Data and Experimental

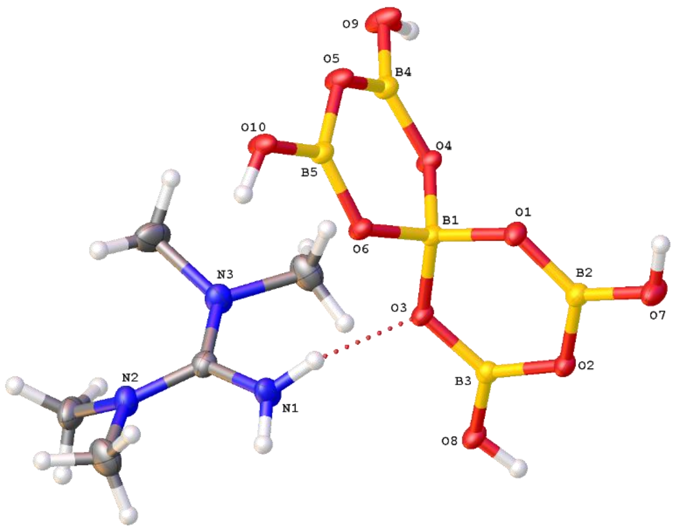

Compound	2011src0322
Formula	$C_5H_{18}B_5N_3O_{10}$
$D_{calc.}$/ g cm^{-3}	1.400
μ/mm^{-1}	0.123
Formula Weight	334.27
Colour	colourless
Shape	block-shaped
Size/mm^3	0.36×0.20×0.14
T/K	120(2)
Crystal System	triclinic
Space Group	P-1
a/Å	9.5035(3)
b/Å	9.5151(3)
c/Å	10.4386(3)
α/°	65.3810(10)
β/°	69.049(2)
γ/°	88.603(2)
V/Å^3	792.72(4)
Z	2
Z'	1
Wavelength/Å	0.71073
Radiation type	MoK_α
Θ_{min}/°	3.071
Θ_{max}/°	27.575
Measured Refl's.	16517
Indep't Refl's	3638
Refl's I≥2 σ(I)	2972
R_{int}	0.0406
Parameters	231
Restraints	0
Largest Peak	0.197
Deepest Hole	-0.238
GooF	1.057
wR_2 (all data)	0.0935
wR_2	0.0873
R_1 (all data)	0.0493
R_1	0.0373

Experimental. Single colourless block-shaped crystals of **2011src0322** were used as supplied. A suitable crystal with dimensions 0.36 × 0.20 × 0.14 mm^3 was selected and mounted on a Bruker-Nonius Roper CCD camera on κ-goniostat diffractometer. The crystal was kept at a steady T = 120(2) K during data collection. The structure was solved with the **ShelXS-97** (Sheldrick, 2008) solution program using direct methods and by using **Olex2** 1.5 (Dolomanov et al., 2009) as the graphical interface. The model was refined with **ShelXL** 2018/3 (Sheldrick, 2015) using full matrix least squares minimisation on $\boldsymbol{F}^2$.

Crystal Data. $C_5H_{18}B_5N_3O_{10}$, M_r = 334.27, triclinic, P-1 (No. 2), a = 9.5035(3) Å, b = 9.5151(3) Å, c = 10.4386(3) Å, α = 65.3810(10)°, β = 69.049(2)°, γ = 88.603(2)°, V = 792.72(4) Å^3, T = 120(2) K, Z = 2, Z' = 1, μ(MoK_α) = 0.123 mm^{-1}, 16517 reflections measured, 3638 unique (R_{int} = 0.0406) which were used in all calculations. The final wR_2 was 0.0935 (all data) and R_1 was 0.0373 (I≥2 σ(I)).

Structure Quality Indicators

**Reflections:**	d min (Mo) 2Θ=55.1° 0.77	I/σ(I) 26.5	Rint m=4.52 4.06%	Full 50.5° 99% to 55.1° 99.7
**Refinement:**	Shift 0.000	Max Peak 0.2	Min Peak -0.2	GooF 1.057

A colourless block-shaped-shaped crystal with dimensions 0.36 × 0.20 × 0.14 mm^3 was mounted. Data were collected using a Bruker-Nonius Roper CCD camera on κ-goniostat diffractometer operating at T = 120(2) K.

Data were measured using ϕ & ω scans with MoK_α radiation. The diffraction pattern was indexed and the total number of runs and images was based on the strategy calculation from the program COLLECT (Hooft, R.W.W., 1998). The maximum resolution that was achieved was Θ = 27.575° (0.77 Å).

The unit cell was refined using DENZO (Otwinowski & Minor, 1997) & COLLECT (Hooft, R.W.W., 1998) on 3558 reflections, 22% of the observed reflections.

Data reduction, scaling and absorption corrections were performed using DENZO (Otwinowski & Minor, 1997) & COLLECT (Hooft, R.W.W., 1998). The final completeness is 99.70 % out to 27.575° in Θ. SADABS 2007/2 (Sheldrick, G.M., 2007). The absorption coefficient μ of this material is 0.123 mm^{-1} at this wavelength (λ = 0.71073Å) and the minimum and maximum transmissions are 0.957 and 0.983.

The structure was solved and the space group P-1 (# 2) determined by the ShelXS-97 (Sheldrick, 2008) structure solution program using direct methods and refined by full matrix least squares minimisation on $\boldsymbol{F^2}$ using version 2018/3 of **ShelXL** (Sheldrick, 2015). All non-hydrogen atoms were refined anisotropically. Most hydrogen atom positions were calculated geometrically and refined using the riding model, but some hydrogen atoms were refined freely.

_exptl_absorpt_process_details: SADABS 2007/2 (Sheldrick, G.M., 2007)

There is a single molecule in the asymmetric unit, which is represented by the reported sum formula. In other words: Z is 2 and Z' is 1.

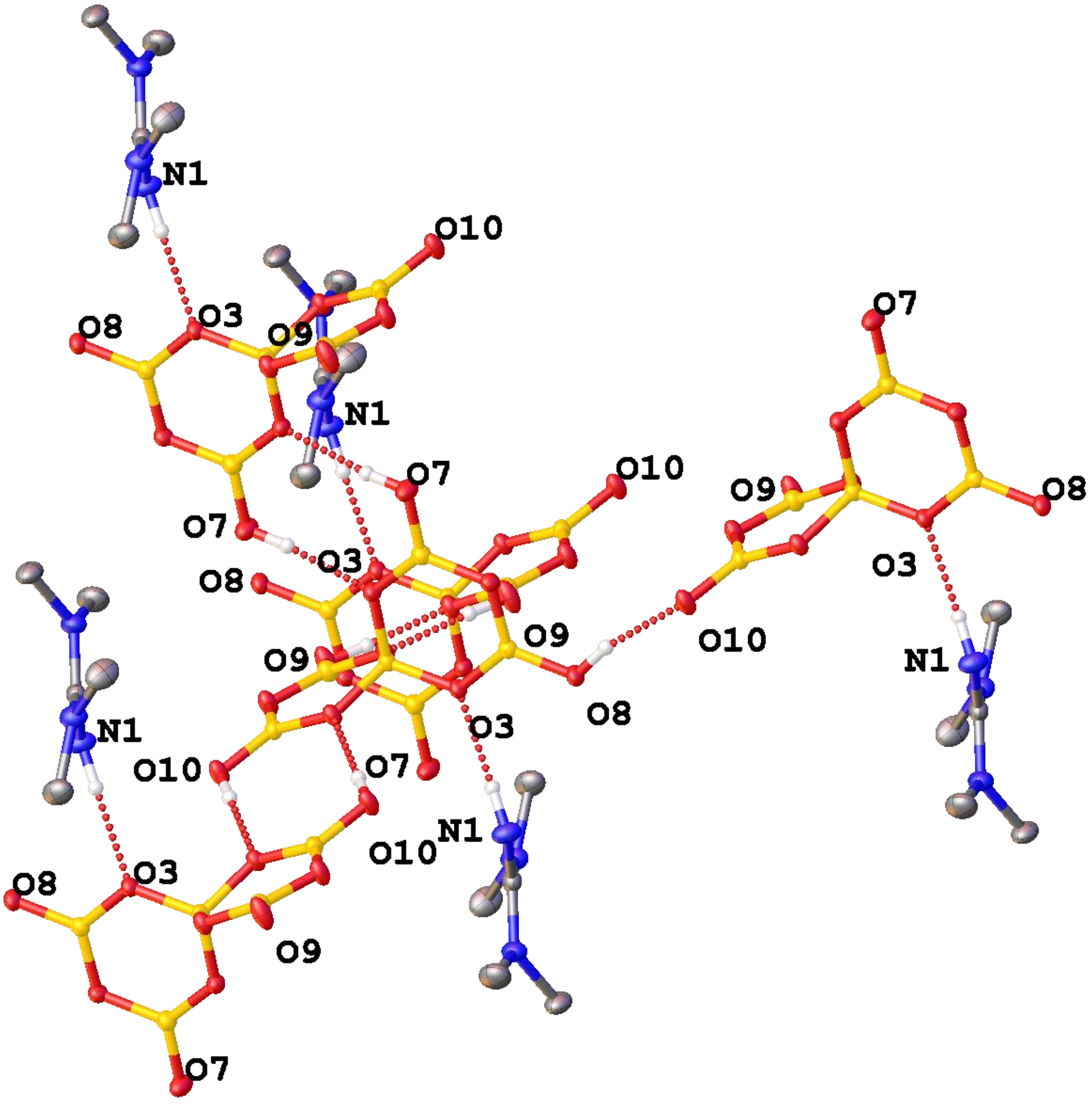

Figure S6: The following hydrogen bonding interactions with a maximum D-D distance of 2.9 Å and a minimum angle of 120 ° are present in **2011src0322**: O7–O1_*1: 2.765 Å, O8–O10*_2: 2.735 Å, O9–O4_*3: 2.706 Å, O10–O6*_4: 2.695 Å, N1–O2_*5: 3.062 Å, N1–O3: 2.827 Å.*

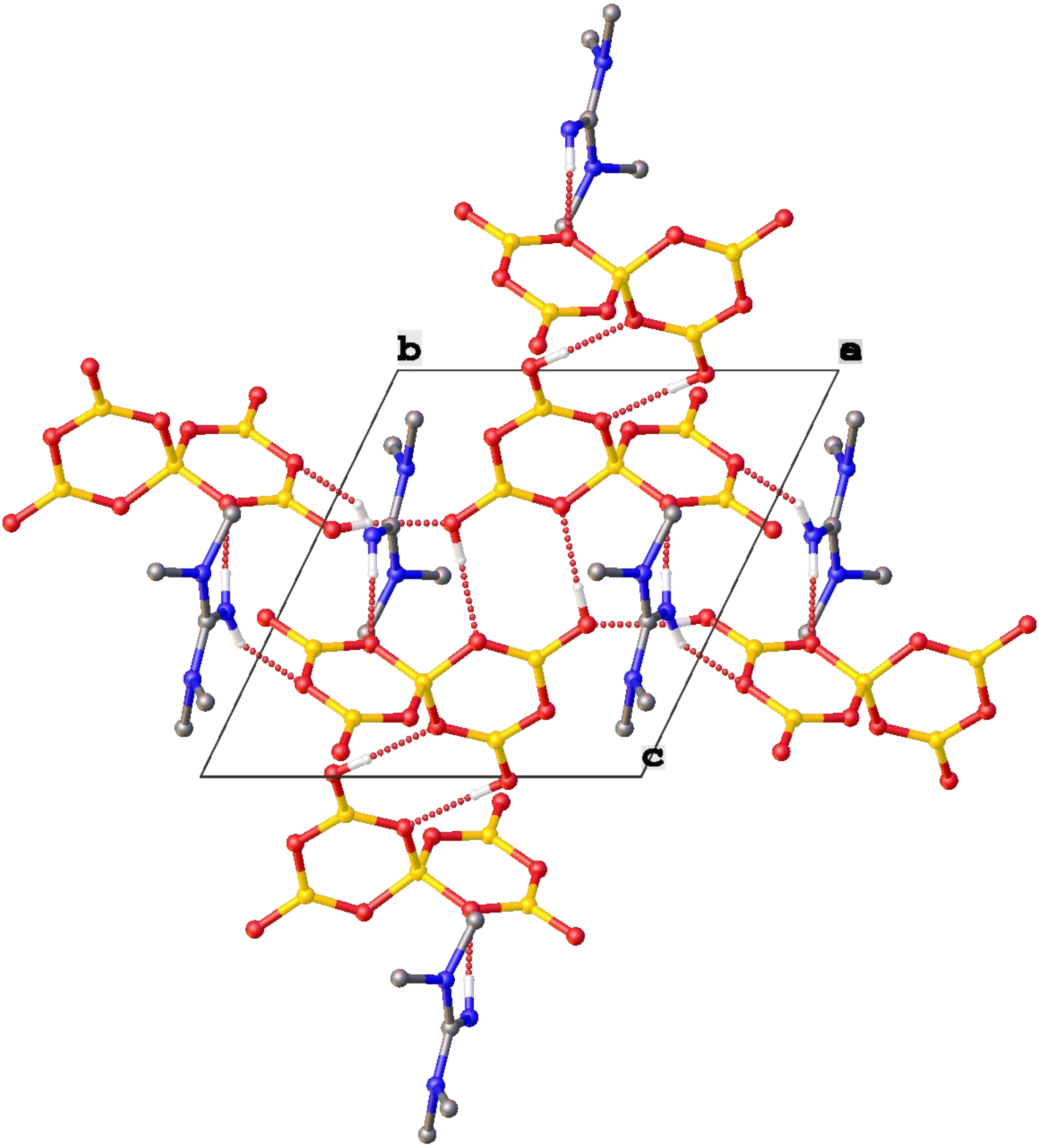

Figure S7: Packing diagram of 2011src0322.

Table S22: Fractional Atomic Coordinates (×10^4) and Equivalent Isotropic Displacement Parameters (Å^2×10^3) for **2011src0322.** U_{eq} is defined as 1/3 of the trace of the orthogonalised U_{ij}.

Atom	x	y	z	U_{eq}
B1	3360.3(16)	5984.1(15)	7668.0(16)	13.6(3)
B2	4894.0(16)	7187.8(16)	8561.2(16)	16.0(3)
B3	3785.7(15)	8851.5(16)	6839.0(16)	13.7(3)
B4	1294.0(16)	3700.8(16)	9092.9(17)	16.9(3)
B5	3507.2(16)	3689.1(16)	7091.4(16)	14.8(3)
O1	4313.3(10)	5910.5(9)	8536.2(10)	16.4(2)
O2	4636.2(10)	8675.7(10)	7724.6(10)	16.3(2)
O3	3270.4(10)	7600.5(9)	6729.6(10)	14.7(2)
O4	1810.4(10)	5184.5(10)	8753.4(10)	15.9(2)
O5	2213.9(10)	2882.7(10)	8369.5(10)	18.7(2)
O6	4014.0(9)	5201.2(9)	6668.9(10)	15.2(2)
O7	5748.5(11)	7096.5(11)	9384.6(12)	24.4(2)
O8	3428.9(10)	10266.4(10)	6079.8(10)	17.4(2)
O9	-130.4(11)	2976.3(11)	10087.3(11)	25.4(2)
O10	4212.4(11)	2912.3(10)	6248.9(11)	19.3(2)
C1	1392.4(14)	8431.0(14)	3827.0(15)	17.5(3)
C2	3052.1(18)	9084(2)	1203.1(17)	32.6(4)
C3	320.6(17)	9288.4(17)	1841.3(17)	27.4(3)
C4	-222.0(17)	7816.1(19)	6469.7(17)	29.9(3)
C5	-1077.9(18)	6736.0(18)	5048.2(19)	34.0(4)
N1	2598.5(13)	8753.3(15)	4074.0(15)	23.0(3)
N2	1537.2(13)	8738.5(13)	2408.1(13)	21.4(3)
N3	48.6(13)	7794.6(14)	5005.7(13)	22.5(3)

Table S23: Anisotropic Displacement Parameters (×10^4) for **2011src0322.** The anisotropic displacement factor exponent takes the form: $-2\pi^2[h^2a^{*2} \times U_{11} + ... + 2hka^* \times b^* \times U_{12}]$$

Atom	U_{11}	U_{22}	U_{33}	U_{23}	U_{13}	U_{12}
B1	15.1(7)	11.3(6)	13.9(7)	-5.6(5)	-4.9(5)	1.0(5)
B2	16.8(7)	15.1(7)	16.9(7)	-7.4(6)	-6.6(6)	1.3(5)
B3	12.5(6)	14.3(6)	14.1(7)	-6.9(5)	-3.9(5)	1.2(5)
B4	18.2(7)	14.5(7)	16.7(7)	-7.2(6)	-4.9(6)	0.6(5)
B5	16.5(7)	12.0(6)	16.7(7)	-6.4(5)	-7.1(6)	2.0(5)
O1	21.2(5)	12.5(4)	18.8(5)	-7.1(4)	-10.9(4)	2.9(3)
O2	20.8(5)	13.5(4)	18.0(5)	-7.3(4)	-10.4(4)	1.4(3)
O3	17.8(4)	11.4(4)	17.3(5)	-6.8(4)	-8.6(4)	1.5(3)
O4	15.7(4)	13.0(4)	17.6(5)	-8.5(4)	-2.7(4)	-0.1(3)
O5	18.7(5)	13.3(4)	20.1(5)	-9.1(4)	-0.6(4)	-1.3(4)
O6	16.6(4)	12.4(4)	15.4(4)	-7.4(4)	-2.9(4)	-0.5(3)
O7	34.3(6)	15.6(5)	29.8(6)	-7.2(4)	-22.7(5)	2.2(4)
O8	21.7(5)	12.4(4)	22.5(5)	-8.8(4)	-12.0(4)	2.9(4)
O9	20.5(5)	18.2(5)	29.6(6)	-14.7(4)	4.6(4)	-3.7(4)
O10	21.4(5)	13.2(4)	17.8(5)	-8.2(4)	0.3(4)	-1.6(4)
C1	18.1(6)	16.4(6)	20.7(7)	-9.2(5)	-9.2(5)	4.6(5)
C2	32.1(8)	42.6(9)	22.8(8)	-16.5(7)	-7.6(7)	7.2(7)
C3	33.3(8)	26.4(7)	30.8(8)	-11.4(6)	-22.6(7)	4.2(6)
C4	25.9(8)	39.5(9)	22.0(7)	-13.7(7)	-6.5(6)	7.6(6)
C5	26.3(8)	32.0(8)	37.8(9)	-6.5(7)	-15.5(7)	-7.3(6)
N1	19.4(6)	28.8(6)	19.2(6)	-6.7(5)	-9.8(5)	-1.9(5)
N2	22.9(6)	25.4(6)	19.9(6)	-11.3(5)	-10.9(5)	3.6(5)
N3	17.5(6)	28.2(6)	21.5(6)	-10.0(5)	-8.1(5)	0.7(5)

Table S24: Bond Lengths in Å for **2011src0322**.

Atom	Atom	Length/Å
B1	O1	1.4757(16)
B1	O3	1.4596(15)
B1	O4	1.4748(16)
B1	O6	1.4799(15)
B2	O1	1.3608(16)
B2	O2	1.3904(17)
B2	O7	1.3562(17)
B3	O2	1.3910(16)
B3	O3	1.3607(15)
B3	O8	1.3552(16)
B4	O4	1.3609(16)
B4	O5	1.3875(16)
B4	O9	1.3526(17)
B5	O5	1.3783(16)
B5	O6	1.3600(15)
B5	O10	1.3656(16)
C1	N1	1.3299(17)
C1	N2	1.3392(17)
C1	N3	1.3434(17)
C2	N2	1.4651(19)
C3	N2	1.4706(17)
C4	N3	1.4652(18)
C5	N3	1.4642(18)

Table S25: Bond Angles in ° for **2011src0322**.

Atom	Atom	Atom	Angle/°
O1	B1	O6	109.67(10)
O3	B1	O1	111.07(9)
O3	B1	O4	108.70(10)
O3	B1	O6	108.97(10)
O4	B1	O1	108.73(10)
O4	B1	O6	109.67(9)
O1	B2	O2	120.95(11)
O7	B2	O1	122.79(12)
O7	B2	O2	116.27(11)
O3	B3	O2	120.80(11)
O8	B3	O2	121.02(11)
O8	B3	O3	118.17(11)
O4	B4	O5	120.35(11)
O9	B4	O4	122.69(12)
O9	B4	O5	116.91(11)
O6	B5	O5	121.24(11)
O6	B5	O10	122.08(12)
O10	B5	O5	116.63(11)
B2	O1	B1	123.55(10)
B2	O2	B3	119.10(10)
B3	O3	B1	123.82(10)
B4	O4	B1	123.03(10)
B5	O5	B4	118.76(10)
B5	O6	B1	121.96(10)
N1	C1	N2	119.51(12)
N1	C1	N3	119.51(12)
N2	C1	N3	120.98(11)
C1	N2	C2	120.24(11)
C1	N2	C3	122.29(12)
C2	N2	C3	114.07(12)
C1	N3	C4	120.99(11)
C1	N3	C5	122.61(12)
C5	N3	C4	114.85(12)

Table S26: Torsion Angles in ° for **2011src0322**.

Atom	Atom	Atom	Atom	Angle/°
O1	B1	O3	B3	-10.07(16)
O1	B1	O4	B4	-100.51(13)
O1	B1	O6	B5	96.39(12)
O1	B2	O2	B3	-0.74(18)
O2	B2	O1	B1	-1.20(19)
O2	B3	O3	B1	9.13(18)
O3	B1	O1	B2	6.11(16)
O3	B1	O4	B4	138.46(11)
O3	B1	O6	B5	-141.83(11)
O3	B3	O2	B2	-3.10(18)
O4	B1	O1	B2	-113.46(12)
O4	B1	O3	B3	109.51(12)
O4	B1	O6	B5	-22.96(15)
O4	B4	O5	B5	-13.65(18)
O5	B4	O4	B1	-2.37(19)
O5	B5	O6	B1	9.66(18)
O6	B1	O1	B2	126.62(12)
O6	B1	O3	B3	-131.00(11)
O6	B1	O4	B4	19.41(16)
O6	B5	O5	B4	10.02(18)
O7	B2	O1	B1	178.94(12)
O7	B2	O2	B3	179.12(11)
O8	B3	O2	B2	175.95(11)
O8	B3	O3	B1	-169.94(11)
O9	B4	O4	B1	-179.85(12)
O9	B4	O5	B5	163.97(12)
O10	B5	O5	B4	-167.61(11)
O10	B5	O6	B1	-172.84(11)
N1	C1	N2	C2	13.89(19)
N1	C1	N2	C3	-144.09(13)
N1	C1	N3	C4	15.85(19)
N1	C1	N3	C5	-149.25(13)
N2	C1	N3	C4	-164.49(13)
N2	C1	N3	C5	30.42(19)
N3	C1	N2	C2	-165.77(12)
N3	C1	N2	C3	36.25(18)

Table S27: Hydrogen Fractional Atomic Coordinates (×10[4]) and Equivalent Isotropic Displacement Parameters (Å[2]×10[3]) for **2011src0322**. U_{eq} is defined as 1/3 of the trace of the orthogonalised U_{ij}.

**Atom**	**x**	**y**	**z**	U_{eq}
H7	5770(20)	6150(20)	9960(20)	37
H8	3703(18)	11002(19)	6244(18)	26
H9	-670(20)	3580(20)	10440(20)	38
H10	4865(19)	3520(20)	5340(20)	29
H2A	3462.83	10184.86	797.74	49
H2B	2976.39	8881.23	381.81	49
H2C	3730.5	8418.65	1623.54	49
H3A	-578.01	9305.43	2669.84	41
H3B	54.03	8581.73	1471.08	41
H3C	674.93	10344.37	1005.19	41
H4A	90.96	6883.36	7112.91	45
H4B	-1308.23	7825.86	6981.42	45
H4C	370.34	8753.5	6293.96	45
H5A	-667.37	6552.88	4135.02	51
H5B	-2011.17	7206.68	5079.05	51
H5C	-1309.26	5740.02	5959.05	51
H1A	3382(19)	9425(19)	3290(20)	28
H1B	2615(18)	8322(19)	5010(20)	28

Table S28: Hydrogen Bond information for **2011src0322**.

D	H	A	d(D-H)/Å	d(H-A)/Å	d(D-A)/Å	D-H-A/deg
O7	H7	O1[1]	0.851(19)	1.92(2)	2.7647(13)	173.5(18)
O8	H8	O10[2]	0.854(17)	1.895(17)	2.7350(12)	167.8(15)
O9	H9	O4[3]	0.86(2)	1.84(2)	2.7060(13)	177.9(18)
O10	H10	O6[4]	0.873(18)	1.828(18)	2.6946(13)	171.1(15)
N1	H1A	O2[5]	0.884(17)	2.253(17)	3.0618(15)	152.0(14)
N1	H1B	O3	0.894(18)	1.959(18)	2.8274(15)	163.7(15)

----

[1]1-x,1-y,2-z; [2]+x,1+y,+z; [3]-x,1-y,2-z; [4]1-x,1-y,1-z; [5]1-x,2-y,1-z

Citations

COLLECT (Hooft, R.W.W., 1998)

O.V. Dolomanov and L.J. Bourhis and R.J. Gildea and J.A.K. Howard and H. Puschmann, Olex2: A complete structure solution, refinement and analysis program, *J. Appl. Cryst.*, (2009), **42**, 339-341.

Sheldrick, G.M., A short history of ShelX, *Acta Cryst.*, (2008), **A64**, 339-341.

Sheldrick, G.M., Crystal structure refinement with ShelXL, *Acta Cryst.*, (2015), **C71**, 3-8.

Z. Otwinowski and W. Minor, Processing of X-ray Diffraction Data Collected in Oscillation Mode, *Methods in Enzymology*, (1997), **276**, 307-326.

UK National Crystallography Service
www.ncs.ac.uk | info@ncs.ac.uk | 023 8059 6722

Submitted by: **Mike A Beckett / Tom Rixon**
Bangor University
Solved by: **Peter N Horton**
Sample ID: **MAB/TR/Me6Gaun B5**

$[C(NHMe)(NMe_2)_2][B_5O_6(OH)_4]\cdot B(OH)_3$ (**5**)

Crystal Data and Experimental

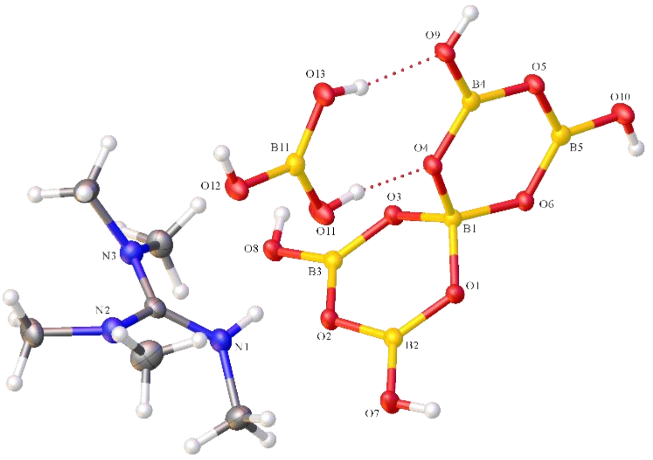

Experimental. Single colourless block-shaped crystals of **2019NCS0283** were crystallised. A suitable crystal 0.220×0.180×0.080 mm^3 was selected and mounted on a MITIGEN holder in perfluoroether oil on a Rigaku 007HF equipped with Varimax confocal mirrors and an AFC11 goniometer and HyPix 6000 detector diffractometer. The crystal was kept at a steady T = 100(2) K during data collection. The structure was solved with the **ShelXT** (Sheldrick, 2015) structure solution program using the dual solution method and by using **Olex2** (Dolomanov et al., 2009) as the graphical interface. The model was refined with version 2018/3 of **ShelXL** (Sheldrick, 2015) using Least Squares minimisation.

Crystal Data. $C_6H_{23}B_6N_3O_{13}$, M_r = 410.13, monoclinic, $P2_1/c$ (No. 14), a = 9.49570(10) Å, b = 11.44900(10) Å, c = 16.84590(10) Å, β = 98.0710(10)°, $\alpha = \gamma$ = 90°, V = 1813.28(3) Å^3, T = 100(2) K, Z = 4, Z' = 1, μ(CuK$_\alpha$) = 1.164 mm^{-1}, 20493 reflections measured, 3281 unique (R_{int} = 0.0196) which were used in all calculations. The final wR_2 was 0.0674 (all data) and R_1 was 0.0263 (I > 2(I)).

**Compound**	**2019NCS0283**
Formula	$C_6H_{23}B_6N_3O_{13}$
$D_{calc.}$/ g cm^{-3}	1.502
μ/mm^{-1}	1.164
Formula Weight	410.13
Colour	colourless
Shape	block
Size/mm^3	0.220×0.180×0.080
T/K	100(2)
Crystal System	monoclinic
Space Group	$P2_1/c$
a/Å	9.49570(10)
b/Å	11.44900(10)
c/Å	16.84590(10)
α/°	90
β/°	98.0710(10)
γ/°	90
V/Å^3	1813.28(3)
Z	4
Z'	1
Wavelength/Å	1.54178
Radiation type	CuK$_\alpha$
Θ_{min}/°	4.685
Θ_{max}/°	68.239
Measured Refl.	20493
Independent Refl.	3281
Reflections with I > 2(I)	3208
R_{int}	0.0196
Parameters	290
Restraints	0
Largest Peak	0.308
Deepest Hole	-0.168
GooF	1.004
wR_2 (all data)	0.0674
wR_2	0.0670
R_1 (all data)	0.0268
R_1	0.0263

Structure Quality Indicators

**Reflections:**	d min (Cu) 0.83	I/σ 93.0	Rint 1.96%	complete 99% (IUCr) 99%
**Refinement:**	Shift -0.001	Max Peak 0.3	Min Peak -0.2	GooF 1.004

A colourless block-shaped crystal with dimensions 0.220×0.180×0.080 mm³ was mounted on a MITIGEN holder in perfluoroether oil. Data were collected using a Rigaku 007HF equipped with Varimax confocal mirrors and an AFC11 goniometer and HyPix 6000 detector diffractometer equipped with an Oxford Cryosystems low-temperature device operating at T = 100(2) K.

Data were measured using profile data from ω-scans 0.5 ° per frame for 0.5 s using CuK_α radiation (Rotating Anode, 40.0 kV, 30.0 mA). The total number of runs and images was based on the strategy calculation from the program **CrysAlisPro** (Rigaku, V1.171.40.45a, 2019). The maximum resolution that was achieved was Θ = 68.239° (0.83 Å).

The diffraction pattern was indexed. The total number of runs and images was based on the strategy calculation from the program **CrysAlisPro** (Rigaku, V1.171.40.45a, 2019) and the unit cell was refined using **CrysAlisPro** (Rigaku, V1.171.40.45a, 2019) on 17068 reflections, 83% of the observed reflections.

Data reduction, scaling and absorption corrections were performed using **CrysAlisPro** (Rigaku, V1.171.40.45a, 2019). The final completeness is 98.70 % out to 68.239° in Θ. A multi-scan absorption correction was performed using CrysAlisPro 1.171.40.45a (Rigaku Oxford Diffraction, 2019) using spherical harmonics as implemented in SCALE3 ABSPACK. The absorption coefficient μ of this material is 1.164 mm^{-1} at this wavelength (λ = 1.54178 Å) and the minimum and maximum transmissions are 0.804 and 1.000.

The structure was solved and the space group $P2_1/c$ (# 14) determined by the **ShelXT** (Sheldrick, 2015) structure solution program using dual and refined by Least Squares using version 2018/3 of **ShelXL** (Sheldrick, 2015). All non-hydrogen atoms were refined anisotropically. Most hydrogen atom positions were calculated geometrically and refined using the riding model, but some hydrogen atoms were refined freely.

_exptl_absorpt_process_details: CrysAlisPro 1.171.40.45a (Rigaku Oxford Diffraction, 2019) using spherical harmonics as implemented in SCALE3 ABSPACK.

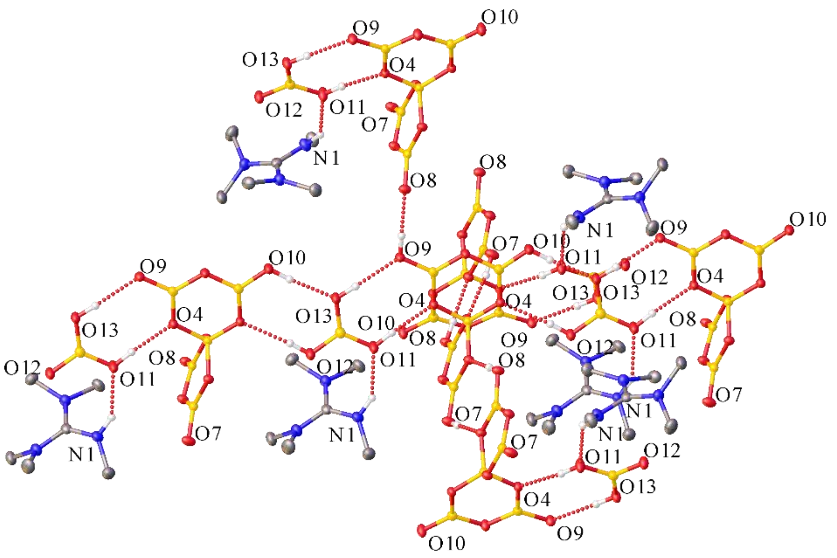

Figure S8: The following hydrogen bonding interactions with a maximum D-D distance of 3.22 Å and a minimum angle of 120 ° are present in **2019NCS0283**: N1–O11: 2.928 Å, O7–O3_*4: 2.711 Å, O8–O1*_1: 2.687 Å, O9–O8_*2: 2.742 Å, O10–O13*_3: 2.725 Å, O11–O4: 2.696 Å, O12–O6_*5: 2.828 Å, O13–O9: 2.799 Å.*

There is a single molecule in the asymmetric unit, which is represented by the reported sum formula. In other words: Z is 4 and Z' is 1.

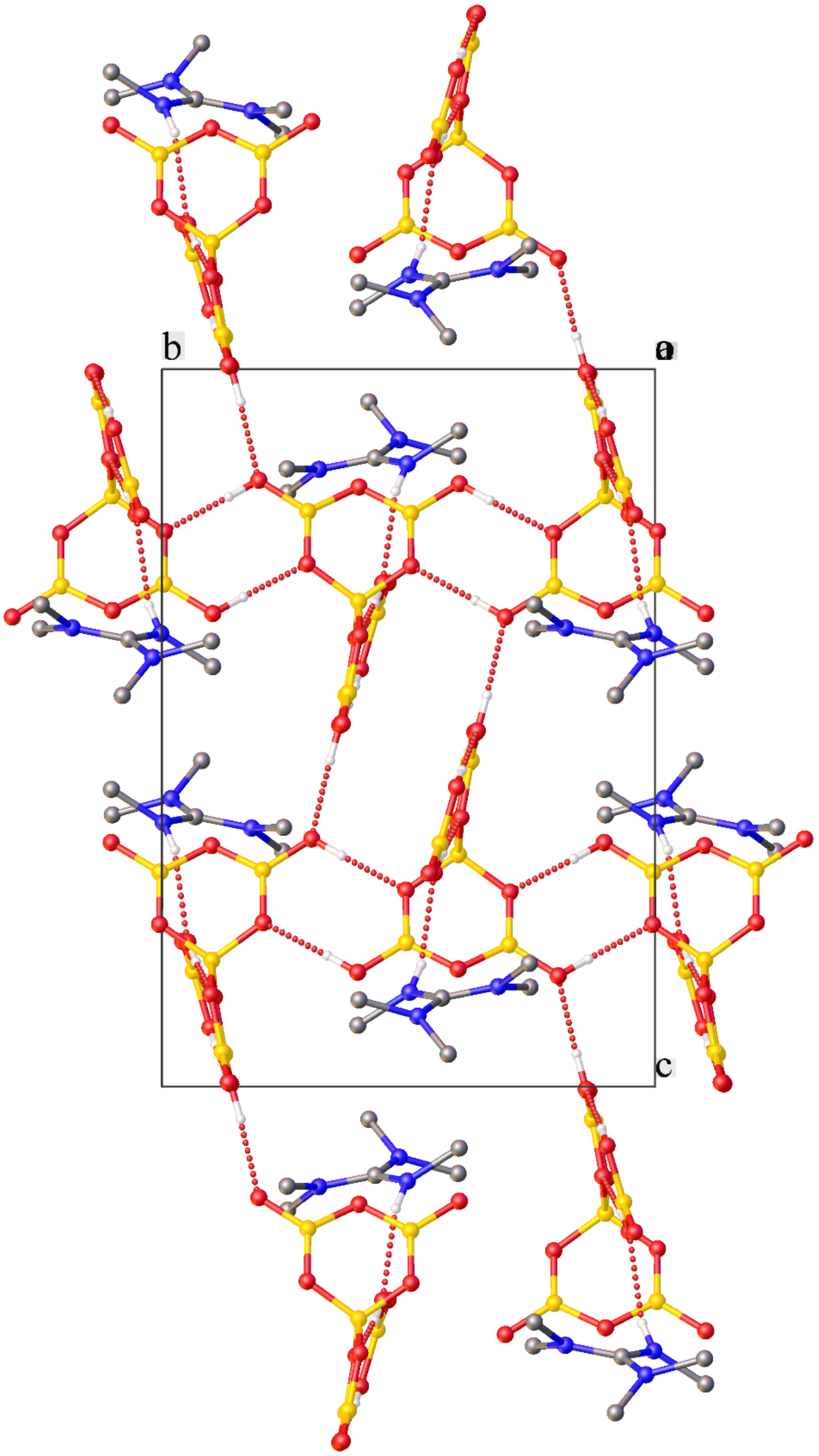

Figure S9: Packing diagram of 2019NCS0283.

Table S29: Fractional Atomic Coordinates (×10^4) and Equivalent Isotropic Displacement Parameters (Å^2×10^3) for **2019NCS0283**. U_{eq} is defined as 1/3 of the trace of the orthogonalised U_{ij}.

Atom	x	y	z	U_{eq}
N(1)	5176.8(10)	5025.2(8)	1316.7(6)	21.4(2)
N(2)	2728.7(10)	5187.1(8)	983.8(5)	20.5(2)
N(3)	4090.2(9)	6818.4(8)	1395.1(5)	19.9(2)
C(1)	4000.1(11)	5675.7(9)	1236.7(6)	16.8(2)
C(2)	5370.0(13)	3973.2(10)	855.4(7)	26.9(3)
C(3)	2400.6(14)	3995.2(10)	1209.5(8)	30.4(3)
C(4)	1625.3(12)	5804.9(11)	453.8(7)	25.1(3)
C(5)	2957.3(12)	7449.3(10)	1719.9(7)	24.1(2)
C(6)	5416.5(12)	7464.6(10)	1386.6(7)	24.8(3)
O(1)	9907.4(7)	4950.1(6)	2739.2(4)	14.19(16)
O(2)	8635.7(8)	6004.7(6)	1639.9(4)	15.71(16)
O(3)	9982.2(7)	7054.5(6)	2712.1(4)	14.23(16)
O(4)	8974.2(7)	6062.1(6)	3750.1(4)	14.17(16)
O(5)	10606.6(7)	6369.6(6)	4939.6(4)	16.54(16)
O(6)	11521.4(7)	5986.8(6)	3710.4(4)	13.74(16)
O(7)	8688.8(8)	3969.8(7)	1595.8(4)	19.66(18)
O(8)	8864.1(8)	8035.3(6)	1546.2(4)	16.50(16)
O(9)	8107.6(7)	6395.4(7)	4962.4(4)	16.50(16)
O(10)	13050.0(8)	6343.7(7)	4941.3(4)	19.88(17)
B(1)	10112.4(12)	6009.5(9)	3235.0(7)	13.0(2)
B(2)	9083.0(12)	4957.3(10)	2007.9(7)	14.3(2)
B(3)	9169.6(12)	7041.9(10)	1979.8(7)	13.5(2)
B(4)	9238.6(12)	6268.8(10)	4548.2(7)	13.6(2)
B(5)	11745.6(12)	6220.3(10)	4513.0(7)	14.8(2)
O(11)	6372.6(8)	5522.8(8)	2980.0(5)	23.99(19)
O(12)	3931.3(8)	5417.3(7)	2974.5(4)	21.08(18)
O(13)	5356.8(8)	5975.7(7)	4176.6(4)	19.80(17)
B(11)	5238.3(12)	5638.2(10)	3388.9(7)	16.8(2)

Table S30: Anisotropic Displacement Parameters (×10[4]) **2019NCS0283**. The anisotropic displacement factor exponent takes the form: $-2\pi^2[h^2a^{*2} \times U_{11} + ... + 2hka^* \times b^* \times U_{12}]$$

Atom	U_{11}	U_{22}	U_{33}	U_{23}	U_{13}	U_{12}
N(1)	18.3(5)	23.6(5)	20.8(5)	-3.5(4)	-2.8(4)	1.1(4)
N(2)	17.6(5)	21.3(5)	22.2(5)	0.4(4)	1.4(4)	-3.2(4)
N(3)	18.8(5)	20.0(5)	20.6(4)	-1.0(4)	1.8(3)	-1.1(4)
C(1)	17.6(5)	20.9(5)	11.6(5)	0.5(4)	1.5(4)	-1.1(4)
C(2)	24.0(6)	26.7(6)	29.3(6)	-6.4(5)	1.7(5)	3.5(5)
C(3)	28.0(6)	23.6(6)	41.2(7)	-0.7(5)	10.0(5)	-7.2(5)
C(4)	15.4(5)	35.3(6)	23.5(6)	1.0(5)	-1.1(4)	-1.1(5)
C(5)	26.9(6)	23.4(6)	22.7(6)	-0.8(4)	5.8(5)	5.2(5)
C(6)	21.4(6)	22.9(6)	28.6(6)	-1.7(5)	-1.5(5)	-5.5(4)
O(1)	15.8(4)	12.8(4)	13.2(3)	-0.5(3)	-0.2(3)	1.0(3)
O(2)	17.5(4)	13.6(4)	14.6(3)	0.0(3)	-2.6(3)	0.4(3)
O(3)	16.5(3)	12.9(4)	12.7(3)	0.7(3)	0.0(3)	-0.9(3)
O(4)	11.6(3)	18.1(4)	12.5(3)	-0.3(3)	0.6(3)	-0.2(3)
O(5)	13.0(4)	23.1(4)	13.1(3)	-2.7(3)	0.3(3)	-0.5(3)
O(6)	12.1(3)	15.8(4)	13.0(3)	-0.1(3)	0.5(3)	0.5(3)
O(7)	25.5(4)	13.3(4)	17.7(4)	-0.7(3)	-5.3(3)	1.1(3)
O(8)	20.8(4)	13.1(4)	14.3(3)	0.7(3)	-2.1(3)	-1.8(3)
O(9)	13.9(4)	23.1(4)	12.1(3)	-2.4(3)	0.5(3)	0.0(3)
O(10)	12.9(4)	30.4(4)	16.0(4)	-4.7(3)	0.5(3)	-0.5(3)
B(1)	13.7(5)	12.6(5)	12.6(5)	0.2(4)	1.0(4)	0.1(4)
B(2)	12.6(5)	16.0(6)	14.3(5)	0.1(4)	1.7(4)	0.2(4)
B(3)	11.8(5)	15.9(5)	13.1(5)	0.0(4)	2.6(4)	0.2(4)
B(4)	14.2(5)	11.6(5)	14.4(5)	0.6(4)	0.4(4)	0.1(4)
B(5)	14.9(5)	13.5(5)	15.6(5)	-0.2(4)	0.9(4)	-0.1(4)
O(11)	13.2(4)	41.7(5)	16.7(4)	-4.6(3)	0.7(3)	-2.3(3)
O(12)	12.9(4)	32.8(4)	17.3(4)	-2.3(3)	1.3(3)	-1.2(3)
O(13)	12.2(4)	30.0(4)	16.6(4)	-2.5(3)	-0.1(3)	1.0(3)
B(11)	15.8(6)	17.2(6)	16.8(5)	1.7(4)	0.4(4)	0.4(4)

Table S31: Bond Lengths in Å for **2019NCS0283**.

Atom	Atom	Length/Å
N(1)	C(1)	1.3338(14)
N(1)	C(2)	1.4589(15)
N(2)	C(1)	1.3444(14)
N(2)	C(3)	1.4619(15)
N(2)	C(4)	1.4603(15)
N(3)	C(1)	1.3355(14)
N(3)	C(5)	1.4642(14)
N(3)	C(6)	1.4625(14)
O(1)	B(1)	1.4700(13)
O(1)	B(2)	1.3638(14)
O(2)	B(2)	1.3889(13)
O(2)	B(3)	1.3833(13)
O(3)	B(1)	1.4805(13)
O(3)	B(3)	1.3602(13)
O(4)	B(1)	1.4792(13)
O(4)	B(4)	1.3535(13)
O(5)	B(4)	1.3765(13)
O(5)	B(5)	1.3906(13)
O(6)	B(1)	1.4599(13)
O(6)	B(5)	1.3652(13)

Atom	Atom	Length/Å
O(7)	B(2)	1.3518(14)
O(8)	B(3)	1.3608(13)
O(9)	B(4)	1.3680(13)
O(10)	B(5)	1.3505(14)
O(11)	B(11)	1.3640(14)
O(12)	B(11)	1.3595(14)
O(13)	B(11)	1.3714(14)

Table S32: Bond Angles in ° for **2019NCS0283**.

**Atom**	**Atom**	**Atom**	**Angle/°**
C(1)	N(1)	C(2)	124.98(10)
C(1)	N(2)	C(3)	121.38(10)
C(1)	N(2)	C(4)	121.84(9)
C(4)	N(2)	C(3)	116.76(9)
C(1)	N(3)	C(5)	121.88(9)
C(1)	N(3)	C(6)	121.65(9)
C(6)	N(3)	C(5)	115.65(9)
N(1)	C(1)	N(2)	120.13(10)
N(1)	C(1)	N(3)	119.83(10)
N(3)	C(1)	N(2)	120.02(10)
B(2)	O(1)	B(1)	121.45(8)
B(3)	O(2)	B(2)	119.02(8)
B(3)	O(3)	B(1)	121.36(8)
B(4)	O(4)	B(1)	122.75(8)
B(4)	O(5)	B(5)	119.50(8)
B(5)	O(6)	B(1)	122.90(8)
O(1)	B(1)	O(3)	109.59(8)
O(1)	B(1)	O(4)	108.51(8)
O(4)	B(1)	O(3)	108.02(8)
O(6)	B(1)	O(1)	109.57(8)
O(6)	B(1)	O(3)	109.53(8)
O(6)	B(1)	O(4)	111.58(8)
O(1)	B(2)	O(2)	120.61(9)
O(7)	B(2)	O(1)	122.78(9)
O(7)	B(2)	O(2)	116.59(9)
O(3)	B(3)	O(2)	120.85(9)
O(3)	B(3)	O(8)	121.94(9)
O(8)	B(3)	O(2)	117.20(9)
O(4)	B(4)	O(5)	121.42(9)
O(4)	B(4)	O(9)	118.41(9)
O(9)	B(4)	O(5)	120.16(9)
O(6)	B(5)	O(5)	120.76(9)
O(10)	B(5)	O(5)	115.63(9)
O(10)	B(5)	O(6)	123.60(10)
O(11)	B(11)	O(13)	123.54(10)
O(12)	B(11)	O(11)	116.83(10)
O(12)	B(11)	O(13)	119.62(10)

Table S33: Torsion Angles in ° for **2019NCS0283**.

Atom	Atom	Atom	Atom	Angle/°
C(2)	N(1)	C(1)	N(2)	28.01(16)
C(2)	N(1)	C(1)	N(3)	-150.84(11)
C(3)	N(2)	C(1)	N(1)	35.28(15)
C(3)	N(2)	C(1)	N(3)	-145.87(11)
C(4)	N(2)	C(1)	N(1)	-142.95(11)
C(4)	N(2)	C(1)	N(3)	35.90(15)
C(5)	N(3)	C(1)	N(1)	-147.90(10)
C(5)	N(3)	C(1)	N(2)	33.25(15)
C(6)	N(3)	C(1)	N(1)	21.33(15)
C(6)	N(3)	C(1)	N(2)	-157.52(10)
B(1)	O(1)	B(2)	O(2)	12.54(14)
B(1)	O(1)	B(2)	O(7)	-169.14(9)
B(1)	O(3)	B(3)	O(2)	-11.22(14)
B(1)	O(3)	B(3)	O(8)	169.78(9)
B(1)	O(4)	B(4)	O(5)	-4.23(15)
B(1)	O(4)	B(4)	O(9)	174.76(8)
B(1)	O(6)	B(5)	O(5)	7.12(15)
B(1)	O(6)	B(5)	O(10)	-171.59(9)
B(2)	O(1)	B(1)	O(3)	-28.44(12)
B(2)	O(1)	B(1)	O(4)	89.30(10)
B(2)	O(1)	B(1)	O(6)	-148.65(9)
B(2)	O(2)	B(3)	O(3)	-7.99(14)
B(2)	O(2)	B(3)	O(8)	171.06(9)
B(3)	O(2)	B(2)	O(1)	7.39(14)
B(3)	O(2)	B(2)	O(7)	-171.03(9)
B(3)	O(3)	B(1)	O(1)	27.81(12)
B(3)	O(3)	B(1)	O(4)	-90.23(10)
B(3)	O(3)	B(1)	O(6)	148.05(8)
B(4)	O(4)	B(1)	O(1)	131.47(9)
B(4)	O(4)	B(1)	O(3)	-109.79(10)
B(4)	O(4)	B(1)	O(6)	10.66(13)
B(4)	O(5)	B(5)	O(6)	0.65(15)
B(4)	O(5)	B(5)	O(10)	179.46(9)
B(5)	O(5)	B(4)	O(4)	-2.04(15)
B(5)	O(5)	B(4)	O(9)	178.98(9)
B(5)	O(6)	B(1)	O(1)	-132.26(9)
B(5)	O(6)	B(1)	O(3)	107.49(10)
B(5)	O(6)	B(1)	O(4)	-12.07(13)

Table S34: Hydrogen Fractional Atomic Coordinates (×10[4]) and Equivalent Isotropic Displacement Parameters (Å[2]×10[3]) for **2019NCS0283**. U_{eq} is defined as 1/3 of the trace of the orthogonalised U_{ij}.

Atom	x	y	z	U_{eq}
H(1)	5865(17)	5261(14)	1701(10)	38(4)
H(2A)	4688.7	3975.06	361.2	40
H(2B)	5210.29	3282.39	1174.56	40
H(2C)	6340.66	3955.34	720.6	40
H(3A)	2445.76	3471.22	753.76	46
H(3B)	1442.42	3971.93	1362.18	46
H(3C)	3093.56	3743.53	1663.81	46
H(4A)	798	5930.35	731.71	38
H(4B)	1343.1	5338.04	-30.06	38
H(4C)	1994.48	6560.76	303.9	38
H(5A)	2491.45	7988.8	1313.68	36
H(5B)	3365.15	7891.08	2195.42	36
H(5C)	2257.03	6890.42	1867.93	36
H(6A)	5900.01	7554.04	1936.6	37
H(6B)	5204.98	8237.44	1149.28	37
H(6C)	6033.2	7035.51	1068.07	37
H(7)	9046(17)	3354(15)	1826(10)	39(4)
H(8)	9244(17)	8646(15)	1779(10)	37(4)
H(9)	8345(17)	6594(14)	5454(10)	41(4)
H(10)	13751(19)	6226(15)	4673(10)	45(5)
H(11)	7180(20)	5688(16)	3258(11)	49(5)
H(12)	3248(19)	5568(15)	3235(10)	41(4)
H(13)	6200(20)	6085(15)	4412(11)	46(5)

Table S35: Hydrogen Bond information for **2019NCS0283**.

D	H	A	d(D-H)/Å	d(H-A)/Å	d(D-A)/Å	D-H-A/deg
N(1)	H(1)	O(11)	0.894(17)	2.162(16)	2.9277(12)	143.2(13)
O(7)	H(7)	O(3)[1]	0.852(18)	1.862(18)	2.7106(10)	173.5(15)
O(8)	H(8)	O(1)[2]	0.857(18)	1.831(18)	2.6873(10)	178.6(16)
O(9)	H(9)	O(8)[3]	0.858(17)	1.885(17)	2.7425(10)	177.7(16)
O(10)	H(10)	O(13)[4]	0.867(18)	1.861(19)	2.7254(11)	175.2(17)
O(11)	H(11)	O(4)	0.860(19)	1.839(19)	2.6958(10)	173.9(17)
O(12)	H(12)	O(6)[5]	0.850(18)	1.982(18)	2.8276(11)	172.7(16)
O(13)	H(13)	O(9)	0.847(19)	1.952(19)	2.7992(10)	177.8(17)

----

[1]2-x,-1/2+y,1/2-z; [2]2-x,1/2+y,1/2-z; [3]+x,3/2-y,1/2+z; [4]1+x,+y,+z; [5]-1+x,+y,+z

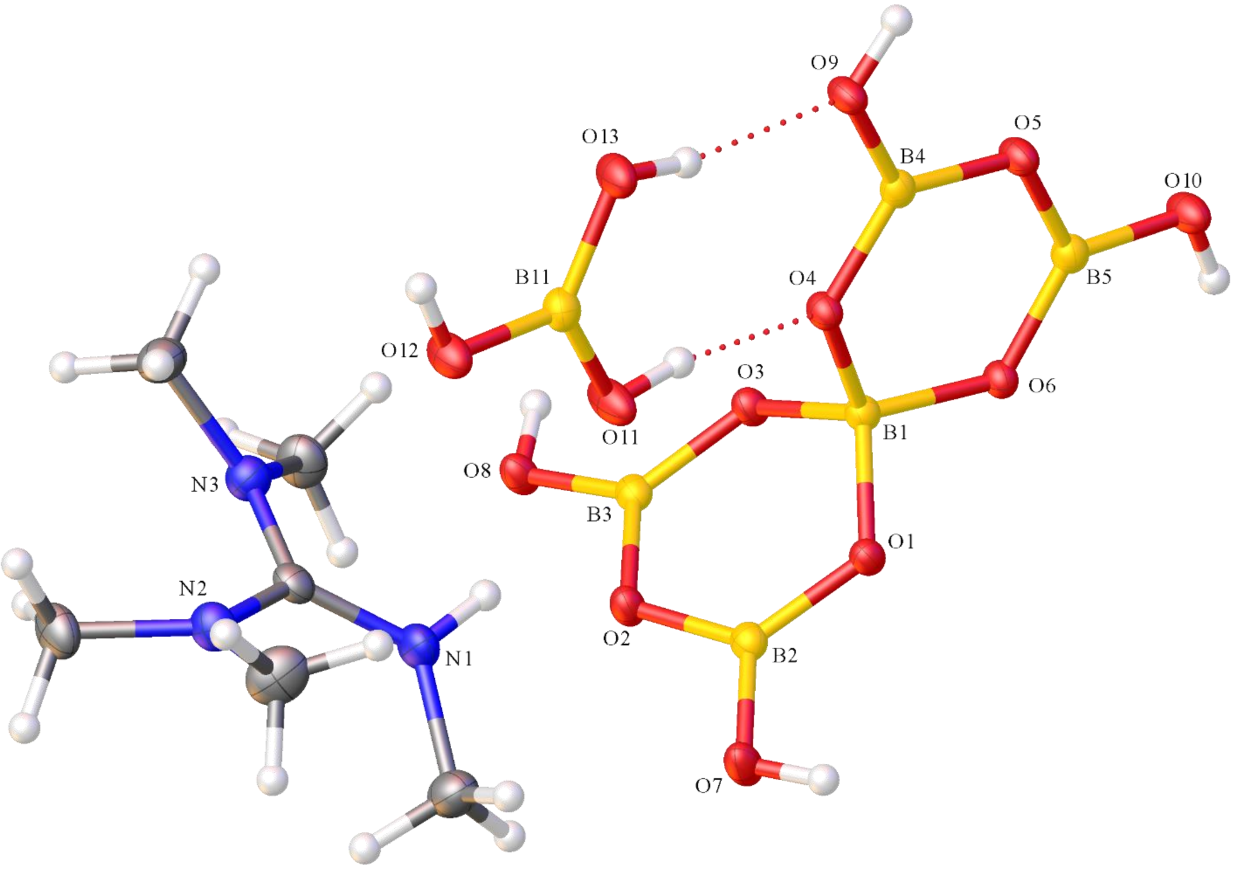

Citations

CrysAlisPro Software System, Rigaku Oxford Diffraction, (2019).

O.V. Dolomanov and L.J. Bourhis and R.J. Gildea and J.A.K. Howard and H. Puschmann, Olex2: A complete structure solution, refinement and analysis program, *J. Appl. Cryst.*, (2009), **42**, 339-341.

Sheldrick, G.M., Crystal structure refinement with ShelXL, *Acta Cryst.*, (2015), **C27**, 3-8.

Sheldrick, G.M., ShelXT-Integrated space-group and crystal-structure determination, *Acta Cryst.*, (2015), **A71**, 3-8.

UK National Crystallography Service
www.ncs.ac.uk | info@ncs.ac.uk | 023 8059 6722

R_1=5.61%

Submitted by: **Mike A Beckett**
Bangor University

Solved by: **Peter N Horton**

Sample ID: **MAB/TR/TABCDEB5**

[TBDH][$B_5O_6(OH)_4$]· (**6**)

Crystal Data and Experimental

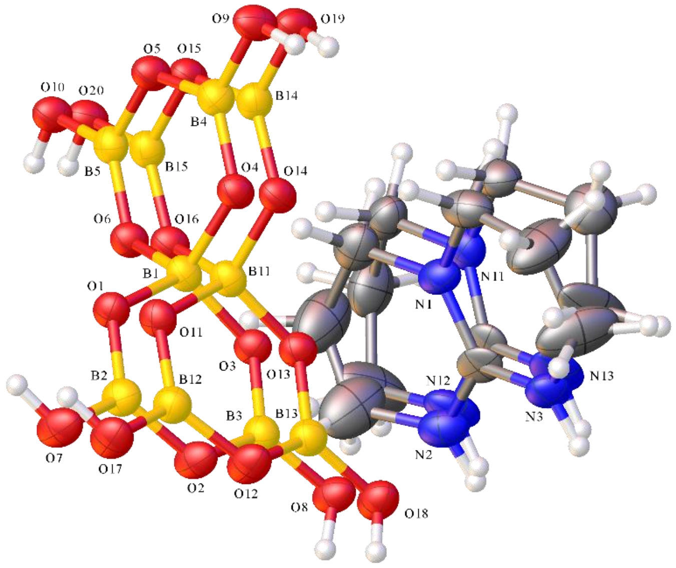

Experimental. Single colourless plate-shaped crystals of **2019ncs0282** were crystallised. A suitable crystal with dimensions 0.21 × 0.11 × 0.02 mm³ was selected and mounted on a Rigaku 007HF equipped with HF Varimax confocal mirrors and an AFC11 goniometer and HyPix 6000HE detector diffractometer. The crystal was kept at a steady T = 99.9(7) K during data collection. The structure was solved with the **ShelXT** 2018/2 (Sheldrick, 2018) solution program using iterative methods and by using **Olex2** 1.5 (Dolomanov et al., 2009) as the graphical interface. The model was refined with **ShelXL** 2018/3 (Sheldrick, 2015) using full matrix least squares minimisation on $\boldsymbol{F^2}$.

Crystal Data. $C_7H_{18}B_5N_3O_{10}$, M_r = 358.29, triclinic, P-1 (No. 2), a = 9.3096(6) Å, b = 9.3175(3) Å, c = 9.3733(6) Å, α = 76.598(5)°, β = 85.611(5)°, γ = 79.947(4)°, V = 778.22(8) Å³, T = 99.9(7) K, Z = 2, Z' = 1, μ(Cu K_α) = 1.133 mm^{-1}, 13382 reflections measured, 2836 unique (R_{int} = 0.0443) which were used in all calculations. The final wR_2 was 0.1747 (all data) and R_1 was 0.0561 (I≥2 σ(I)).

**Compound**	**2019ncs0282**
Formula	$C_7H_{18}B_5N_3O_{10}$
$D_{calc.}$/ g cm^{-3}	1.529
μ/mm^{-1}	1.133
Formula Weight	358.29
Colour	colourless
Shape	plate-shaped
Size/mm³	0.21×0.11×0.02
T/K	99.9(7)
Crystal System	triclinic
Space Group	P-1
a/Å	9.3096(6)
b/Å	9.3175(3)
c/Å	9.3733(6)
α/°	76.598(5)
β/°	85.611(5)
γ/°	79.947(4)
V/Å³	778.22(8)
Z	2
Z'	1
Wavelength/Å	1.54178
Radiation type	Cu K_α
Θ_{min}/°	4.828
Θ_{max}/°	68.221
Measured Refl's.	13382
Indep't Refl's	2836
Refl's I≥2 σ(I)	2429
R_{int}	0.0443
Parameters	459
Restraints	1234
Largest Peak	0.349
Deepest Hole	-0.307
GooF	1.076
wR_2 (all data)	0.1747
wR_2	0.1677
R_1 (all data)	0.0616
R_1	0.0561

Structure Quality Indicators

**Reflections:**	d min (Cu\a) 2Θ=136.4° 0.83	I/σ(I) 33.2	Rint m=4.72 4.43%	Full 135.4° 99.3
**Refinement:**	Shift 0.000	Max Peak 0.3	Min Peak -0.3	GooF 1.076

A colourless plate-shaped-shaped crystal with dimensions 0.21 × 0.11 × 0.02 mm^3 was mounted. Data were collected using a Rigaku 007HF equipped with HF Varimax confocal mirrors and an AFC11 goniometer and HyPix 6000HE detector diffractometer operating at T = 99.9(7) K.

Data were measured using profile data from ω-scans with Cu K_α radiation. The diffraction pattern was indexed and the total number of runs and images was based on the strategy calculation from the program CrysAlisPro 1.171.40.45a (Rigaku OD, 2019). The maximum resolution that was achieved was Θ = 68.221° (0.83 Å).

The unit cell was refined using CrysAlisPro 1.171.40.45a (Rigaku OD, 2019) on 6687 reflections, 50% of the observed reflections.

Data reduction, scaling and absorption corrections were performed using CrysAlisPro 1.171.40.45a (Rigaku OD, 2019). The final completeness is 99.30 % out to 68.221° in Θ. A multi-scan absorption correction was performed using CrysAlisPro 1.171.40.45a (Rigaku Oxford Diffraction, 2019) Empirical absorption correction using spherical harmonics, implemented in SCALE3 ABSPACK scaling algorithm.. The absorption coefficient μ of this material is 1.133 mm^{-1} at this wavelength (λ = 1.54178Å) and the minimum and maximum transmissions are 0.803 and 1.000.

The structure was solved and the space group P-1 (# 2) determined by the ShelXT 2018/2 (Sheldrick, 2018) structure solution program using iterative methods and refined by full matrix least squares minimisation on $\boldsymbol{F^2}$ using version 2018/3 of **ShelXL** 2018/3 (Sheldrick, 2015). All non-hydrogen atoms were refined anisotropically. Hydrogen atom positions were calculated geometrically and refined using the riding model.

_*refine_special_details*: All crystals attempted were modulated. This was not taken into consideration during the integration which just used the sub-cell. The structure can be reasonably modelled as two components of whole molecule disorder using both geometrical (SADI, DFIX, FLAT) and displacement (RIGU, SIMU) restraints.

_*exptl_absorpt_process_details*: CrysAlisPro 1.171.40.45a (Rigaku Oxford Diffraction, 2019) using spherical harmonics as implemented in SCALE3 ABSPACK.

There is a single molecule in the asymmetric unit, which is represented by the reported sum formula. In other words: Z is 2 and Z' is 1.

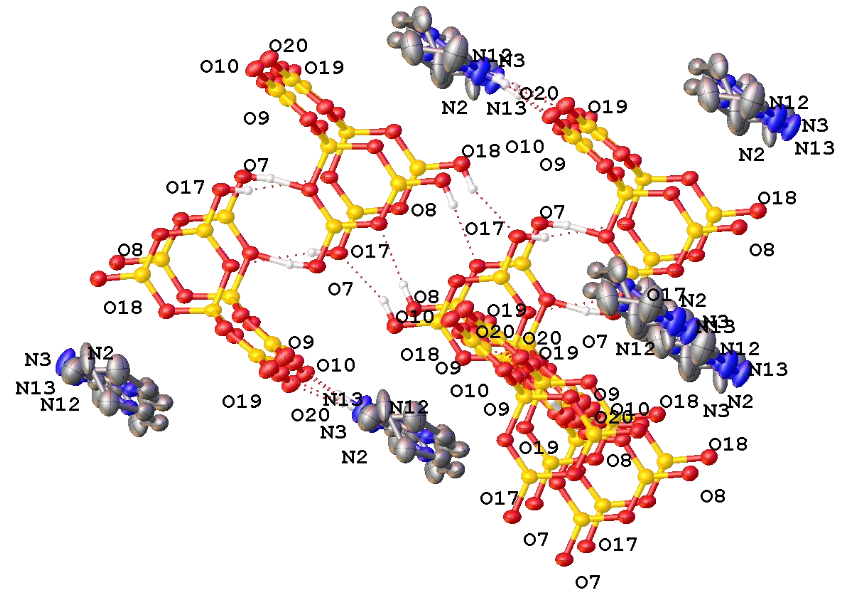

Figure S10: The following hydrogen bonding interactions with a maximum D-D distance of 2.9 Å and a minimum angle of 120 ˚ are present in **2019ncs0282**: O7–O1_1: 2.094 Å, O8–O2_5: 2.876 Å, O9–O4_2: 2.965 Å, O10–O6_3: 2.828 Å, N12–O20_4: 3.315 Å, N13–O15_4: 3.221 Å, O17–O11_1: 3.368 Å, O18–O17_5: 2.873 Å, O19–O14_2: 2.653 Å, O20–O16_3: 2.787 Å, N2–O10_4: 2.853 Å, N3–O5_4: 2.775 Å.

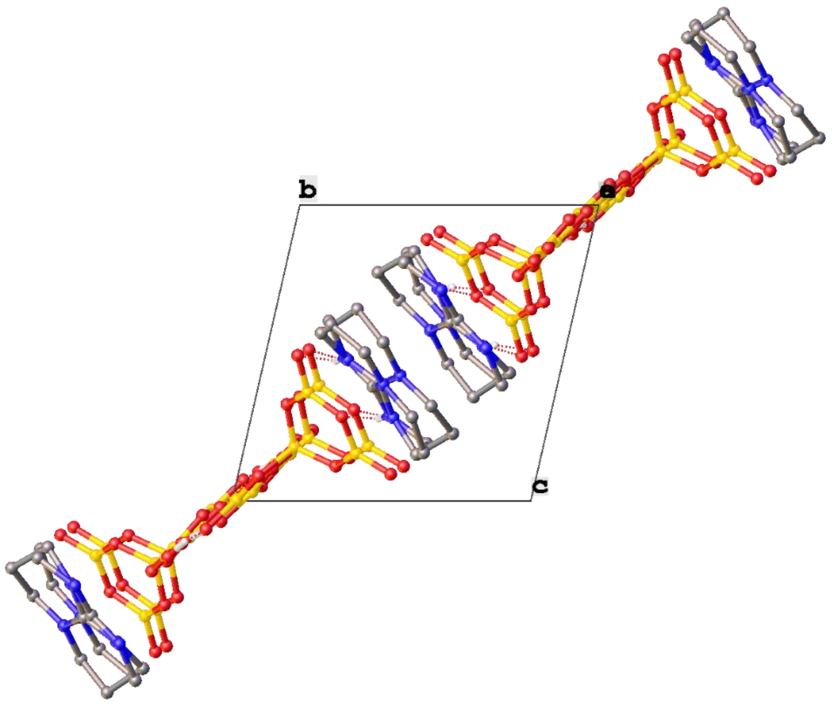

Figure S11: Packing diagram of 2019ncs0282.

Table S36: Fractional Atomic Coordinates (×10⁴) and Equivalent Isotropic Displacement Parameters (Å²×10³) for **2019ncs0282**. U_{eq} is defined as 1/3 of the trace of the orthogonalised U_{ij}.

**Atom**	**x**	**y**	**z**	U_{eq}
O1	4376(3)	9307(3)	9166(3)	28.6(6)
O2	1823(4)	9649(3)	9775(3)	33.6(6)
O3	2723(3)	8275(3)	7953(3)	28.0(5)
O4	4957(3)	6800(3)	8826(3)	28.1(6)
O5	6996(4)	6973(4)	7150(4)	30.1(8)
O6	4952(3)	8908(3)	6739(3)	28.2(6)
O7	3374(3)	10660(4)	10966(3)	38.8(6)
O8	221(4)	8637(3)	8552(3)	33.9(6)
O9	7042(4)	4898(4)	9101(4)	35.0(8)
O10	7029(5)	8917(5)	5082(5)	31.1(9)
B1	4216(5)	8327(5)	8173(5)	27.2(9)
B2	3222(5)	9861(6)	9969(6)	29.9(8)
B3	1591(5)	8847(6)	8770(7)	28.8(8)
B4	6296(5)	6216(6)	8390(5)	29.0(10)
B5	6297(6)	8298(6)	6325(6)	28.0(10)
N11	2207(4)	5331(4)	5768(4)	33.4(8)
N12	222(12)	7212(10)	5228(10)	42.3(17)
N13	85(12)	5248(10)	7188(9)	44.8(18)
C11	848(10)	5937(11)	6052(10)	37.6(18)
C12	1070(8)	8305(10)	4325(8)	73(2)
C13	2213(5)	7283(6)	3512(5)	55.3(10)
C14	3122(5)	6160(5)	4634(5)	41.3(9)
C15	742(6)	4186(9)	8430(6)	63.3(18)
C16	1946(4)	3133(5)	7724(5)	51.7(9)
C17	2991(4)	4065(5)	6793(5)	40.7(9)
O11	3892(3)	8904(3)	8896(3)	28.1(6)
O12	1345(3)	9123(4)	9508(4)	37.5(7)
O13	2311(3)	7848(3)	7616(3)	28.7(6)
O14	4566(3)	6407(3)	8544(3)	28.0(6)
O15	6667(4)	6618(4)	6923(4)	29.9(7)
O16	4566(3)	8533(3)	6480(3)	27.1(6)
O17	2826(3)	10216(3)	10701(4)	36.9(6)
O18	-219(3)	8274(3)	8102(3)	36.1(6)
O19	6672(4)	4525(4)	8837(4)	34.6(8)
O20	6701(5)	8588(5)	4891(6)	32.7(9)
B11	3794(6)	7921(5)	7881(4)	26.4(9)
B12	2714(8)	9404(5)	9693(5)	29.7(8)
B13	1155(7)	8416(5)	8412(5)	29.4(8)
B14	5937(5)	5861(5)	8147(5)	27.6(9)
B15	5950(6)	7969(6)	6101(6)	27.5(10)
N1	2066(5)	5835(4)	6067(4)	34.8(9)
N2	-58(12)	7323(12)	5153(12)	55(3)
N3	-192(12)	5382(11)	7119(11)	52(2)
C1	623(9)	6165(10)	6127(9)	33.3(18)
C2	725(8)	8136(11)	3921(11)	90(3)
C3	2195(7)	8050(8)	4033(7)	77.3(16)
C4	3005(5)	6699(6)	4988(5)	43.9(10)
C5	415(7)	3934(8)	8064(8)	65.0(16)
C6	1865(6)	3959(7)	8373(6)	69.8(13)
C7	2850(5)	4622(6)	7167(5)	44.0(10)

Table S37: Anisotropic Displacement Parameters (×10[4]) for **2019ncs0282.** The anisotropic displacement factor exponent takes the form: $-2\pi^2[h^2a^{*2} \times U_{11} + ... + 2hka^* \times b^* \times U_{12}]$

**Atom**	U_{11}	U_{22}	U_{33}	U_{23}	U_{13}	U_{12}
O1	31.2(14)	30.9(13)	26.3(12)	-11.9(10)	2.9(9)	-6.4(10)
O2	30.2(18)	43.6(15)	31.9(13)	-20.7(11)	0.2(11)	-3.0(14)
O3	28.9(16)	30.1(13)	26.0(12)	-10.0(10)	2.8(10)	-3.6(11)
O4	30.0(14)	25.8(14)	26.7(13)	-5.8(10)	3.9(9)	-1.8(10)
O5	31.8(18)	27.6(18)	27.2(15)	-4.2(12)	5.3(12)	-0.6(12)
O6	29.7(15)	28.8(14)	24.7(13)	-6.8(10)	4.5(10)	-2.8(10)
O7	32.8(14)	53.8(16)	37.9(15)	-28.9(12)	2.6(10)	-5.7(12)
O8	26.9(16)	43.0(15)	35.4(14)	-18.7(11)	0.7(11)	-3.0(12)
O9	34.1(18)	30.4(17)	32.0(17)	1.5(12)	6.7(12)	2.9(12)
O10	32(2)	29(2)	28.1(16)	-3.1(13)	6.8(13)	-1.4(13)
B1	33(2)	25(2)	22(2)	-3.2(16)	0.8(16)	-5.0(16)
B2	32(2)	34(2)	25(2)	-11.4(17)	-1.0(17)	-4.1(18)
B3	30(2)	30(2)	26(2)	-7.2(18)	-1.6(18)	-4.3(17)
B4	32(2)	30(2)	25(2)	-7.2(16)	-1.3(16)	-3.5(16)
B5	33(3)	29(3)	24(2)	-8.5(16)	-1.1(16)	-6.0(16)
N11	31.9(17)	38(2)	34.1(19)	-16.4(15)	3.7(13)	-6.6(15)
N12	38(4)	48(3)	42(3)	-18(3)	-2(2)	1(2)
N13	41(4)	51(3)	47(3)	-26(3)	13(2)	-5(2)
C11	35(3)	43(3)	39(3)	-17(2)	2(2)	-7(2)
C12	43(3)	86(4)	70(4)	39(3)	-33(2)	-22(3)
C13	54(2)	65(3)	48(2)	2(2)	2.5(17)	-33(2)
C14	38(2)	49(2)	44(2)	-22.4(19)	9.1(16)	-14.4(18)
C15	43(3)	110(4)	35(3)	13(2)	-10.0(19)	-45(3)
C16	49(2)	55(2)	44(2)	7.2(18)	-9.1(16)	-11.0(17)
C17	34.7(19)	42(2)	46(2)	-15.6(18)	-5.6(16)	1.6(17)
O11	33.8(15)	28.4(13)	24.6(13)	-12.2(11)	3.6(11)	-5.0(10)
O12	32.6(16)	48.8(17)	39.4(17)	-28.6(14)	8.7(13)	-8.3(11)
O13	28.9(14)	29.0(12)	28.0(12)	-8.5(10)	3.5(9)	-3.4(9)
O14	31.6(15)	25.4(13)	25.6(13)	-5.3(10)	4.3(10)	-3.7(10)
O15	33.1(18)	25.1(17)	28.4(16)	-4.3(11)	6.4(12)	-1.9(11)
O16	29.9(15)	27.5(14)	21.9(13)	-4.1(10)	3.8(10)	-3.4(10)
O17	36.1(15)	44.0(15)	38.4(15)	-25.9(12)	7.5(13)	-9.2(11)
O18	31.1(14)	41.7(14)	40.6(14)	-19.6(11)	5.0(10)	-8.6(10)
O19	34.0(18)	29.0(17)	34.8(18)	-1.8(11)	8.6(12)	-0.6(11)
O20	34(2)	29(2)	30.5(18)	-1.8(13)	6.2(14)	-1.3(13)
B11	32(3)	26(2)	21.8(18)	-8.4(15)	2.8(15)	-2.5(17)
B12	31(3)	30(2)	29(2)	-11.2(16)	2.5(18)	-4(2)
B13	29(3)	30(2)	28(2)	-6.6(16)	1.1(17)	-3.4(18)
B14	32(2)	29(2)	23(2)	-9.3(16)	1.6(16)	-5.4(16)
B15	33(3)	27(2)	24(2)	-8.6(16)	1.8(17)	-4.2(17)
N1	31.0(19)	41(2)	35.1(19)	-17.4(15)	0.1(13)	-0.5(16)
N2	30(3)	66(4)	54(4)	12(3)	-5(2)	2(2)
N3	29(3)	58(3)	55(3)	9(2)	9.9(19)	-2(2)
C1	37(3)	43(3)	24(3)	-20(2)	4(2)	-2(2)
C2	58(4)	73(4)	110(7)	30(4)	-3(4)	-4(3)
C3	74(4)	83(4)	70(3)	19(3)	-23(3)	-37(3)
C4	45(2)	53(3)	41(2)	-23.0(19)	10.7(16)	-16(2)
C5	54(3)	67(3)	66(4)	-5(3)	0(3)	-3(3)
C6	61(3)	71(3)	68(3)	11(3)	-24(2)	-12(2)
C7	39(2)	47(3)	46(2)	-19.2(18)	-6.7(16)	5.7(18)

Table S38: Bond Lengths in Å for **2019ncs0282**.

Atom	Atom	Length/Å
O1	B1	1.478(6)
O1	B2	1.360(6)
O2	B2	1.383(5)
O2	B3	1.381(8)
O3	B1	1.432(5)
O3	B3	1.360(7)
O4	B1	1.481(5)
O4	B4	1.344(6)
O5	B4	1.392(6)
O5	B5	1.378(6)
O6	B1	1.491(5)
O6	B5	1.349(6)
O7	B2	1.352(7)
O8	B3	1.361(6)
O9	B4	1.352(6)
O10	B5	1.364(7)
N11	C11	1.327(10)
N11	C14	1.470(6)
N11	C17	1.459(6)
N12	C11	1.323(11)
N12	C12	1.468(11)
N13	C11	1.333(12)
N13	C15	1.440(10)
C12	C13	1.574(9)
C13	C14	1.495(6)
C15	C16	1.568(8)
C16	C17	1.509(6)

Atom	Atom	Length/Å
O11	B11	1.483(5)
O11	B12	1.354(6)
O12	B12	1.376(8)
O12	B13	1.379(5)
O13	B11	1.438(6)
O13	B13	1.362(6)
O14	B11	1.484(5)
O14	B14	1.348(5)
O15	B14	1.396(6)
O15	B15	1.399(6)
O16	B11	1.488(5)
O16	B15	1.358(6)
O17	B12	1.360(6)
O18	B13	1.368(7)
O19	B14	1.354(6)
O20	B15	1.350(7)
N1	C1	1.324(9)
N1	C4	1.472(6)
N1	C7	1.470(6)
N2	C1	1.339(11)
N2	C2	1.448(12)
N3	C1	1.320(11)
N3	C5	1.478(10)
C2	C3	1.368(7)
C3	C4	1.485(7)
C5	C6	1.407(6)
C6	C7	1.488(7)

Table S39: Bond Angles in ° for **2019ncs0282**.

Atom	Atom	Atom	Angle/°
B2	O1	B1	121.8(4)
B3	O2	B2	119.8(4)
B3	O3	B1	123.0(5)
B4	O4	B1	123.6(3)
B5	O5	B4	120.2(4)
B5	O6	B1	123.2(3)
O1	B1	O4	108.0(3)
O1	B1	O6	107.6(4)
O3	B1	O1	112.8(3)
O3	B1	O4	109.0(4)
O3	B1	O6	109.6(3)
O4	B1	O6	109.7(3)
O1	B2	O2	120.8(6)
O7	B2	O1	122.3(4)
O7	B2	O2	116.9(4)
O3	B3	O2	120.7(4)
O3	B3	O8	118.5(7)
O8	B3	O2	120.8(5)
O4	B4	O5	120.3(4)
O4	B4	O9	123.5(4)
O9	B4	O5	116.2(4)
O6	B5	O5	120.6(4)
O6	B5	O10	123.1(5)
O10	B5	O5	116.2(5)

Atom	Atom	Atom	Angle/°
C11	N11	C14	120.3(6)
C11	N11	C17	122.0(6)
C17	N11	C14	115.5(4)
C11	N12	C12	122.4(9)
C11	N13	C15	123.7(9)
N11	C11	N13	119.6(9)
N12	C11	N11	121.5(9)
N12	C11	N13	118.9(9)
N12	C12	C13	101.6(7)
C14	C13	C12	108.6(4)
N11	C14	C13	111.4(4)
N13	C15	C16	104.0(5)
C17	C16	C15	108.7(4)
N11	C17	C16	111.0(3)
B12	O11	B11	121.8(4)
B12	O12	B13	119.1(4)
B13	O13	B11	122.4(4)
B14	O14	B11	123.8(3)
B14	O15	B15	118.9(4)
B15	O16	B11	123.6(3)
O11	B11	O14	107.6(3)
O11	B11	O16	107.1(4)
O13	B11	O11	112.5(3)
O13	B11	O14	109.9(4)

Atom	Atom	Atom	Angle/°
O13	B11	O16	110.2(3)
O14	B11	O16	109.5(3)
O11	B12	O12	121.3(4)
O11	B12	O17	121.7(6)
O17	B12	O12	117.0(5)
O13	B13	O12	121.5(6)
O13	B13	O18	118.5(5)
O18	B13	O12	120.0(4)
O14	B14	O15	121.2(4)
O14	B14	O19	123.3(4)
O19	B14	O15	115.3(4)
O16	B15	O15	120.7(4)
O20	B15	O15	115.1(5)
O20	B15	O16	124.0(5)

Atom	Atom	Atom	Angle/°
C1	N1	C4	123.6(6)
C1	N1	C7	121.2(6)
C7	N1	C4	115.0(4)
C1	N2	C2	121.7(9)
C1	N3	C5	122.0(9)
N1	C1	N2	119.9(9)
N3	C1	N1	122.4(9)
N3	C1	N2	117.7(8)
C3	C2	N2	117.4(8)
C2	C3	C4	118.5(6)
N1	C4	C3	113.8(4)
C6	C5	N3	109.4(7)
C5	C6	C7	118.9(5)
N1	C7	C6	112.7(4)

Table S40: Torsion Angles in ° for **2019ncs0282**.

Atom	Atom	Atom	Atom	Angle/°
B1	O1	B2	O2	-6.5(5)
B1	O1	B2	O7	174.7(3)
B1	O3	B3	O2	6.4(5)
B1	O3	B3	O8	-174.9(3)
B1	O4	B4	O5	-8.2(7)
B1	O4	B4	O9	171.7(4)
B1	O6	B5	O5	8.0(7)
B1	O6	B5	O10	-173.2(4)
B2	O1	B1	O3	11.0(4)
B2	O1	B1	O4	-109.5(4)
B2	O1	B1	O6	132.1(3)
B2	O2	B3	O3	-0.7(5)
B2	O2	B3	O8	-179.4(3)
B3	O2	B2	O1	0.9(5)
B3	O2	B2	O7	179.8(3)
B3	O3	B1	O1	-11.0(4)
B3	O3	B1	O4	109.0(4)
B3	O3	B1	O6	-130.9(4)
B4	O4	B1	O1	-100.5(4)
B4	O4	B1	O3	136.6(4)
B4	O4	B1	O6	16.5(6)
B4	O5	B5	O6	2.6(7)
B4	O5	B5	O10	-176.3(4)
B5	O5	B4	O4	-2.6(7)
B5	O5	B4	O9	177.6(4)
B5	O6	B1	O1	100.8(4)
B5	O6	B1	O3	-136.1(4)
B5	O6	B1	O4	-16.4(6)
N12	C12	C13	C14	-61.9(6)
N13	C15	C16	C17	58.5(6)
C11	N11	C14	C13	-25.3(6)
C11	N11	C17	C16	27.4(6)
C11	N12	C12	C13	48.7(7)
C11	N13	C15	C16	-46.1(7)
C12	N12	C11	N11	-22.9(7)
C12	N12	C11	N13	157.4(7)
C12	C13	C14	N11	53.6(6)
C14	N11	C11	N12	7.7(5)
C14	N11	C11	N13	-172.6(4)

Atom	Atom	Atom	Atom	Angle/˚
C14	N11	C17	C16	-169.6(4)
C15	N13	C11	N11	22.5(7)
C15	N13	C11	N12	-157.8(7)
C15	C16	C17	N11	-51.7(5)
C17	N11	C11	N12	169.9(5)
C17	N11	C11	N13	-10.4(5)
C17	N11	C14	C13	171.5(4)
B11	O11	B12	O12	-5.4(5)
B11	O11	B12	O17	175.2(3)
B11	O13	B13	O12	1.2(5)
B11	O13	B13	O18	-178.9(3)
B11	O14	B14	O15	-9.1(7)
B11	O14	B14	O19	174.7(4)
B11	O16	B15	O15	9.6(7)
B11	O16	B15	O20	-174.4(4)
B12	O11	B11	O13	12.5(4)
B12	O11	B11	O14	-108.6(4)
B12	O11	B11	O16	133.7(3)
B12	O12	B13	O13	7.1(5)
B12	O12	B13	O18	-172.7(3)
B13	O12	B12	O11	-4.9(5)
B13	O12	B12	O17	174.5(3)
B13	O13	B11	O11	-10.4(4)
B13	O13	B11	O14	109.4(4)
B13	O13	B11	O16	-129.8(3)
B14	O14	B11	O11	-99.9(4)
B14	O14	B11	O13	137.3(4)
B14	O14	B11	O16	16.1(6)
B14	O15	B15	O16	-0.3(6)
B14	O15	B15	O20	-176.5(4)
B15	O15	B14	O14	0.0(6)
B15	O15	B14	O19	176.5(4)
B15	O16	B11	O11	100.0(4)
B15	O16	B11	O13	-137.4(4)
B15	O16	B11	O14	-16.4(6)
N2	C2	C3	C4	27.2(14)
N3	C5	C6	C7	-42.9(9)
C1	N1	C4	C3	4.2(7)
C1	N1	C7	C6	-5.6(6)
C1	N2	C2	C3	-22.0(15)
C1	N3	C5	C6	33.5(10)
C2	N2	C1	N1	7.1(10)
C2	N2	C1	N3	-172.5(10)
C2	C3	C4	N1	-18.8(10)
C4	N1	C1	N2	1.3(6)
C4	N1	C1	N3	-179.1(5)
C4	N1	C7	C6	170.1(4)
C5	N3	C1	N1	-11.0(9)
C5	N3	C1	N2	168.6(9)
C5	C6	C7	N1	31.0(8)
C7	N1	C1	N2	176.7(5)
C7	N1	C1	N3	-3.8(6)
C7	N1	C4	C3	-171.4(4)

Table S41: Hydrogen Fractional Atomic Coordinates (×10⁴) and Equivalent Isotropic Displacement Parameters (Å²×10³) for **2019ncs0282**. U_{eq} is defined as 1/3 of the trace of the orthogonalised U_{ij}.

**Atom**	**x**	**y**	**z**	U_{eq}
H7	4253.05	10758.92	10973	58
H8	-394.88	9217.73	8941.43	51
H9	6545.73	4535.72	9840.89	52
H10	6510.69	9701.69	4640.91	47
H12	-736.72	7412.54	5220.16	51
H13	-873.99	5454.67	7178.59	54
H12A	1556.88	8796.04	4938.35	87
H12B	448.87	9077.1	3621.07	87
H13A	1699.7	6766.24	2944.55	66
H13B	2842.33	7899.83	2821.6	66
H14A	3780.71	5449.06	4146.38	50
H14B	3733.11	6678.27	5100.92	50
H15A	13.59	3615.96	9019.79	76
H15B	1180.74	4693.17	9065.21	76
H16A	2478.13	2366.05	8503.92	62
H16B	1487.62	2617.29	7111.1	62
H17A	3567.21	4435.8	7435.02	49
H17B	3676.94	3437.14	6238.53	49
H17	3663.19	10454.1	10625.49	55
H18	-837.2	8719.8	8619.19	54
H19	6217.35	4198.81	9620.8	52
H20	6166.81	9334.98	4407.2	49
H2	-1005.37	7598.21	5265.39	66
H3	-1125.8	5734.64	7219.5	62
H2A	569.09	7773.4	3040.78	108
H2B	276.98	9203.24	3746.35	108
H3A	2351.08	8929.96	4388.48	93
H3B	2648.61	8143.83	3032.23	93
H4A	3539.6	6043.34	4362.44	53
H4B	3735.7	7007.92	5518.88	53
H5A	-179.35	3748.58	8990.11	78
H5B	388.1	3115.83	7559.55	78
H6A	2324.49	2915.78	8774.62	84
H6B	1832.28	4508.73	9164.14	84
H7A	3600.82	5014.75	7590.78	53
H7B	3355.92	3828.2	6679.26	53

Table S42: Hydrogen Bond information for **2019ncs0282**.

**D**	**H**	**A**	**d(D-H)/Å**	**d(H-A)/Å**	**d(D-A)/Å**	**D-H-A/deg**
O7	H7	O1[1]	0.84	1.27	2.094(4)	168.0
O8	H8	O2[2]	0.84	2.05	2.876(4)	169.8
O9	H9	O4[3]	0.84	2.16	2.965(4)	159.2
O10	H10	O6[4]	0.84	2.02	2.828(6)	160.9
N12	H12	O20[5]	0.88	2.45	3.315(12)	165.9
N13	H13	O15[5]	0.88	2.36	3.221(11)	165.8
O17	H17	O11[1]	0.84	2.55	3.368(4)	165.3
O18	H18	O17[2]	0.84	2.07	2.873(4)	159.4
O19	H19	O14[3]	0.84	1.82	2.653(5)	171.3
O20	H20	O16[4]	0.84	1.99	2.787(6)	158.8
N2	H2	O10[5]	0.88	2.01	2.853(12)	159.2
N3	H3	O5[5]	0.88	1.92	2.775(11)	165.1

----

[1]1-x,2-y,2-z; [2]-x,2-y,2-z; [3]1-x,1-y,2-z; [4]1-x,2-y,1-z; [5]-1+x,+y,+z

Table S43: Atomic Occupancies for all atoms that are not fully occupied in **2019ncs0282**.

Atom	Occupancy
O1	0.5
O2	0.5
O3	0.5
O4	0.5
O5	0.5
O6	0.5
O7	0.5
H7	0.5
O8	0.5
H8	0.5
O9	0.5
H9	0.5
O10	0.5
H10	0.5
B1	0.5
B2	0.5
B3	0.5
B4	0.5
B5	0.5
N11	0.5
N12	0.5
H12	0.5
N13	0.5
H13	0.5
C11	0.5
C12	0.5
H12A	0.5
H12B	0.5
C13	0.5
H13A	0.5
H13B	0.5
C14	0.5
H14A	0.5
H14B	0.5
C15	0.5
H15A	0.5
H15B	0.5
C16	0.5
H16A	0.5
H16B	0.5
C17	0.5
H17A	0.5
H17B	0.5
O11	0.5
O12	0.5
O13	0.5
O14	0.5
O15	0.5
O16	0.5
O17	0.5
H17	0.5
O18	0.5
H18	0.5
O19	0.5
H19	0.5
O20	0.5
H20	0.5
B11	0.5
B12	0.5
B13	0.5
B14	0.5
B15	0.5
N1	0.5
N2	0.5
H2	0.5
N3	0.5
H3	0.5
C1	0.5
C2	0.5
H2A	0.5
H2B	0.5
C3	0.5
H3A	0.5
H3B	0.5
C4	0.5
H4A	0.5
H4B	0.5
C5	0.5
H5A	0.5
H5B	0.5
C6	0.5
H6A	0.5
H6B	0.5
C7	0.5
H7A	0.5
H7B	0.5

Citations

CrysAlisPro (Rigaku, V1.171.40.45a, 2019)

O.V. Dolomanov and L.J. Bourhis and R.J. Gildea and J.A.K. Howard and H. Puschmann, Olex2: A complete structure solution, refinement and analysis program, *J. Appl. Cryst.*, (2009), **42**, 339-341.

Sheldrick, G.M., Crystal structure refinement with ShelXL, *Acta Cryst.*, (2015), **C71**, 3-8.

Sheldrick, G.M., ShelXT-Integrated space-group and crystal-structure determination, *Acta Cryst.*, (2015), **A71**, 3-8.
